# Microbiology Galaxy Lab: a community-driven gateway for reproducible and FAIR analysis of microbiological data

**DOI:** 10.1038/s42003-026-10750-7

**Published:** 2026-10-29

**Authors:** Engy M. T. Nasr, Nikolaos Pechlivanis, Nikolaos Strepis, Pierre Amato, Matthias Bernt, Anshu Bhardwaj, Daniel Blankenberg, Daniela Brites, Fabio Cumbo, Katherine T. Do, Emanuele Ferrari, Timothy J. Griffin, Björn A. Grüning, Saskia Hiltemann, Cameron J. Hyde, Pratik D. Jagtap, Ashwani Kumar, Muneer A. Malla, Subina Mehta, Kimberly L. Métris, Saim Momin, Tiffanie Nelson, Asime J. Oba, Christina Pavloudi, Raphaëlle Péguilhan, Gareth R. Price, Fotis Psomopoulos, Nedeljka Rosic, Michael C. Schatz, Valerie Claudia Schiml, Cléa Siguret, Nicola Soranzo, Andrew P. Stubbs, Peter van Heusden, Mustafa Vohra, Paul F. Zierep, Bérénice Batut

**Affiliations:** 1 Bioinformatics Group, Department of Computer Science, University of Freiburg, Freiburg, Germany; 2 Institute of Applied Biosciences, Centre for Research and Technology Hellas, Thessaloniki, Greece; 3 Department of Pathology and Clinical Bioinformatics, Erasmus MC Cancer Institute, Rotterdam, Netherlands; 4 Université Clermont Auvergne, CNRS, Laboratoire Microorganismes: Génomes et Environnement (LMGE), Clermont-Ferrand, France; 5 Department of Computational Biology and Chemistry, Helmholtz Centre for Environmental Research - UFZ, Leipzig, Germany; 6 Bioinformatics Centre, CSIR-Institute of Microbial Technology (IMTECH), Chandigarh, India; 7 Center for Computational Life Sciences, Cleveland Clinic Research, Cleveland Clinic, Cleveland, OH USA; 8 Swiss Tropical and Public Health Institute, University of Basel, Basel, Switzerland; 9 University of Minnesota, Minneapolis, MN USA; 10 National Research Council of Italy – Water Research Institute (CNR-IRSA) Molecular Ecology Group (MEG), Verbania, Italy; 11 University of Freiburg, Faculty of Chemistry and Pharmacy, Freiburg, Germany; 12 Queensland Cyber Infrastructure Foundation, Brisbane, QLD Australia; 13 Metagenomics and Secretomics Research Laboratory, Department of Botany, University of Allahabad (A Central University), Prayagraj, Uttar Pradesh India; 14 German Centre for Integrative Biodiversity Research (iDiv), Leipzig, Germany; 15 Department of Genetics and Biochemistry, Clemson University, Clemson, SC USA; 16 School of Health Research, Clemson University, Clemson South Carolina, SC USA; 17 Australian BioCommons, The University of Melbourne, Melbourne, VIC Australia; 18 University of Maiduguri, Maiduguri, Nigeria; 19 European Marine Biological Resource Centre (EMBRC-ERIC), Paris, France; 20 Department of Chemical and Biochemical Engineering, Technical University of Denmark, Kgs. Lyngby, Denmark; 21 The University of Queensland, Brisbane, QLD Australia; 22 Faculty of Health, Southern Cross University, Gold Coast, QLD Australia; 23 Department of Computer Science, Johns Hopkins University, Baltimore, MD USA; 24 The Protein Engineering and Proteomics Group (PEP), Faculty of Chemistry, Biotechnology and Food Science, Norwegian University of Life Sciences, Ås, Norway; 25 IFB-core, Institut Français de Bioinformatique (IFB), CNRS, INSERM, INRAE, CEA, Villejuif, France; 26 Plateforme AuBi, Mésocentre Clermont-Auvergne, Université Clermont, Aubière, Auvergne France; 27 Earlham Institute, Norwich, UK; 28 South African National Bioinformatics Institute, University of the Western Cape, Cape Town, South Africa; 29 Department of Medical Laboratory Science, Lovely Professional University, Punjab India; 30 Department of Microbiology, Shri Vinoba Bhave Civil Hospital, Silvassa, India

**Keywords:** Microbiology, Computational platforms and environments

## Abstract

The explosion of microbial omics data has outpaced the ability of many researchers to analyse it, with complex tools and limited computational resources creating barriers to discovery. To address this gap, we present the Microbiology Galaxy Lab (MGL): a free, globally accessible, community-supported platform that combines state-of-the-art analytical power with user-friendly accessibility. Supported by the Galaxy and global microbiology communities, this platform integrates over 315 tool suites and 115 curated workflows, enabling comprehensive metabarcoding, (meta)genomic, (meta)transcriptomic, and (meta)proteomic data analysis within a FAIR-aligned environment. It also supports research in the health and infectious disease sectors, as well as in environmental microbiology. The platform’s utility is exemplified through various use cases, including antimicrobial resistance tracking, biomarker prediction, microbiome classification, and functional annotation of key microbes. Built on reproducibility and community engagement, it supports the creation, sharing, and updating of best-practice workflows. Over 35 tutorials and learning paths empower scientists, fostering an ecosystem that keeps resources at the forefront of microbiological data analysis. The MGL enables collective analysis, democratising research, thereby accelerating discovery across the global microbiology community (microbiology.usegalaxy.org, .eu, .org.au, .fr).

**Figure:**
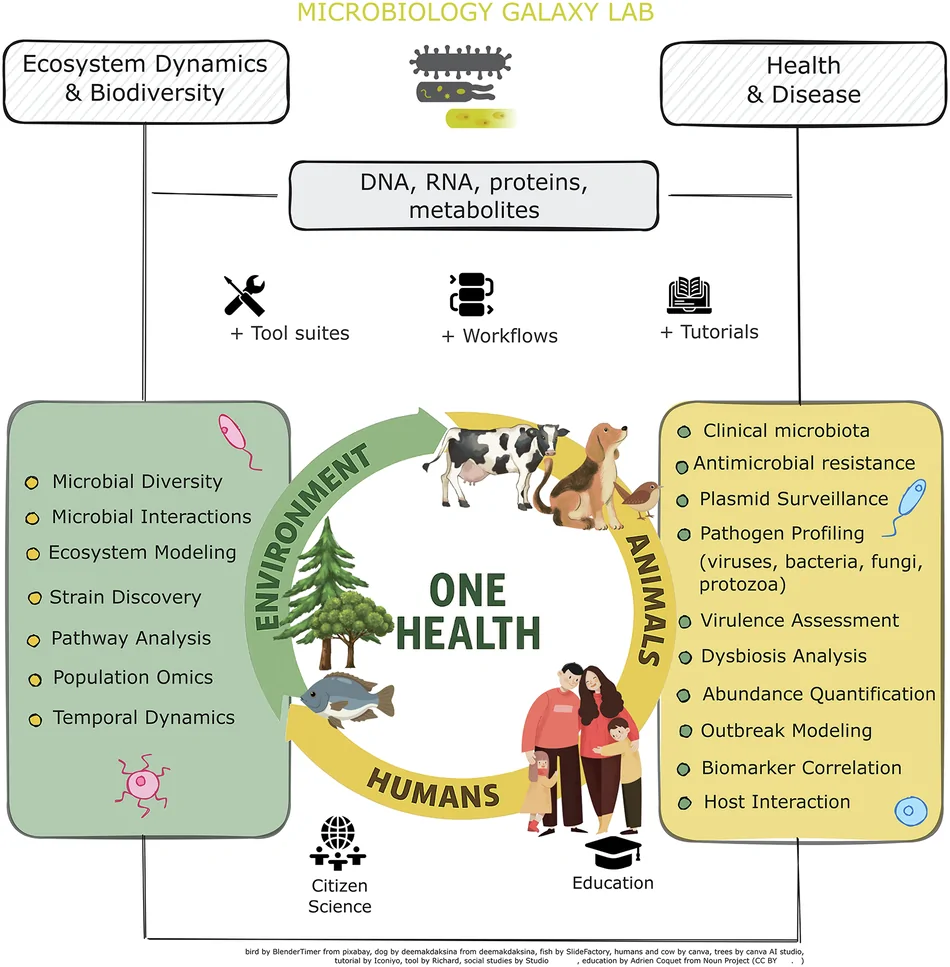

## Abstract (web summary)

Microbiology Galaxy Lab provides a community-driven, democratized ecosystem of tools, workflows, training, and computational infrastructure enabling accessible and reproducible microbial research across health, environmental, agricultural, food, and One Health domains.

## Introduction

Over the past two decades, advances in molecular biology and high-throughput sequencing technologies have transformed microbiology^1,2^, enabling enhanced microbial analysis in health and ecosystem contexts. To extract meaningful insights, such as microbiome profiles or microbial pathogens, scalable bioinformatics tools are essential for processing diverse, large-scale datasets. However, a critical bottleneck persists: many microbiologists lack the expertise for these complex analyses, a challenge exacerbated by the need for appropriate bioinformatics tools, computational resources, and reference databases^3,4^. Closing the bioinformatics knowledge gap and making powerful analytical pipelines widely available is therefore essential, and the microbiology field is increasingly focusing on open, standardised computing environments^5^. Effective solutions must be robust, accurate, reproducible, and adhere to the Findable, Accessible, Interoperable, Reusable (**FAIR**) principles^6^ (bold abbreviation explained in Table 1) to reduce complexity in data organisation, annotation, and tool compatibility. Healthcare and research centres generate vast microbiological datasets, providing a crucial foundation for understanding pathogen spread and working towards pandemic preparedness^7^. While whole genome sequencing (**WGS**) has been integrated into routine diagnostic or surveillance studies^7^, its full potential is often unrealized due to limitations in bioinformatics expertise and infrastructure^8^. Global disparities in methodologies and computational access further hinder researchers’ ability to perform large-scale analyses^3^. Key steps, such as quality assessment, genome/plasmid/metagenome assembly, and resistance/virulence trait detection, are essential for understanding complex infections and pathogen spread. When combined with other omics (e.g., proteomics), these analyses can reveal functional pathogen mechanisms for clinical applications. Given the pathogen–host interactions complexity, a systems‑biology approach integrating multi-omics layers is necessary^9,10^ to capture taxonomic, functional, and expression‑level insights. Reproducible, scalable workflows are also critical for unlocking microbial biodiversity and ecosystem function insights. Environmental microbiology increasingly leverages multi-omic technologies (e.g., (meta)genomics, (meta)transcriptomics, (meta)proteomics, metabolomics) to explore community composition, functional traits, and responses to stressors like pollution and climate change^11–13^. These approaches advance our understanding of microbial roles in nutrient cycling, carbon dynamics, or environmental resilience^12^, while data integration from diverse environments (e.g., wastewater, plant, atmosphere) further clarifies disease and infection emergence mechanisms^14,15^. Interdisciplinary frameworks like One Health^16^ benefit from such integrated data analysis, supporting research across public health, agriculture, and environmental systems. To meet the demand for flexible, accessible microbial bioinformatics, several platforms have emerged (Supplementary Data 1). While these platforms excel in specific domains, many are limited to specific omics modalities, lack interoperability, or operate as “black boxes”, reducing transparency and user control^17^. Additionally, computational expertise remains a barrier to adoption, highlighting the need for open, integrated, user-friendly solutions.

**Table 1:** Glossary of abbreviations and terms

*AI*	Artificial intelligence, is a branch of computer science focused on building computational systems capable of performing tasks that typically require human intellect
*AMR*	Antimicrobial Resistance^105^, the ability of microorganisms to resist the effects of antimicrobial agents
*API*	Application Programming Interface^106^, a defined set of rules enabling software systems to communicate and exchange data
*AST*	Antimicrobial Susceptibility Testing^107^, a laboratory method used to determine microbial sensitivity to antimicrobial compounds
*Biocontainers*	A community-driven container framework for bioinformatics software environments^91^
*Bioconda*	Bioinformatics Conda-based distribution channel for bioinformatics software packages^90^
*CI*	Continuous Integration^108^, an automated process for testing and validating software changes
*CoDex*	Galaxy Communities Dock^95^, a curated catalogue of Galaxy resources (tool suites, trainings, and workflows)
*Dockstore*	A free and open-source workflow registry for sharing and publishing reusable and scalable analytical workflows^31^
*DOI*	Digital Object Identifier, a string of numbers, letters, and symbols used to permanently identify an article or document and link to it on the web
*DRS*	Data Repository Service, a GA4GH standard API for accessing and exchanging genomic data (https://www.ga4gh.org/product/data-repository-service-drs/)
*EDAM*	An ontology for bioinformatics describing operations, data types, topics, and formats^72^
*ELIXIR*	European life-sciences research infrastructure for biological information, coordinating life-science data resources and services^68^
*EMBL-EBI*	European Molecular Biology Laboratory – European Bioinformatics Institute^109^, a major European centre for bioinformatics research and data services
*ENA*	European Nucleotide Archive^21^, a public repository for nucleotide sequencing data
*FAIR*	Findable, Accessible, Interoperable, and Reusable^6^, principles for scientific data management and stewardship
*GA4GH*	Global Alliance for Genomics and Health^110^, an international consortium developing standards for genomic data sharing
*GTN*	Galaxy Training Network^64^, a community-driven platform providing Galaxy training materials and tutorials
*IUC*	Intergalactic Utilities Commission, a Galaxy governance group that reviews and maintains tools to ensure quality and sustainability within the Galaxy ecosystem (https://galaxyproject.org/iuc/)
*IWC*	Intergalactic Workflow Commission, a Galaxy governance group that reviews, curates, and maintains workflows to ensure quality, reproducibility, and best practices (https://iwc.galaxyproject.org/)
*LIMS*	Laboratory Information Management System^111^, software for managing laboratory workflows and data
*MAGs*	Metagenome-Assembled Genomes^112^, genomes reconstructed from metagenomic sequencing data
*MGE*	Mobile Genetic Element^113^, genetic material capable of moving within or between genomes
*MGL*	Microbiology Galaxy Lab, a Galaxy-based platform providing microbiology-focused tools, workflows, and training available at microbiology.usegalaxy.org, .eu, .org.au, .fr
*NCBI*	National Center for Biotechnology Information^20^, a U.S. institution providing biomedical databases and resources
*PIDs*	Persistent Identifiers, stable and long-lasting identifiers for digital resources
*RIR*	Recommended Interoperability Resource^114^, an ELIXIR designation for standards-compliant data services
*RO-Crate*	Research Object Crate, a standardised packaging format for research data and metadata
*SDK*	Software Development Kit, a collection of software development tools and libraries in one installable package
*SIG*	Special Interest Group, a community subgroup focused on a specific topic within Galaxy
*SRA*	Sequence Read Archive, a public repository for raw sequencing data (https://www.ncbi.nlm.nih.gov/sra)
*TeSS*	Training eSupport System^115^, an ELIXIR registry for life-science training resources
*TES*	Task Execution Service^116^, a GA4GH standard API for standardised computational task execution
*TRS*	Tool Registry Service, a GA4GH standard API for sharing and retrieving workflows and tools (https://www.ga4gh.org/product/tool-registry-service-trs/)
*UCSC*	University of California Santa Cruz^19^, Genomics Institute hosting the UCSC Genome Browser and genomics resources
*WGS*	Whole Genome Sequencing^117^, sequencing of an organism’s complete genomic DNA
*WorkflowHub*	An open registry for sharing, publishing, and citing computational workflows^30^
*Zenodo*	An open-access research repository for datasets, software, and publications^118^

**Fig. 1:**
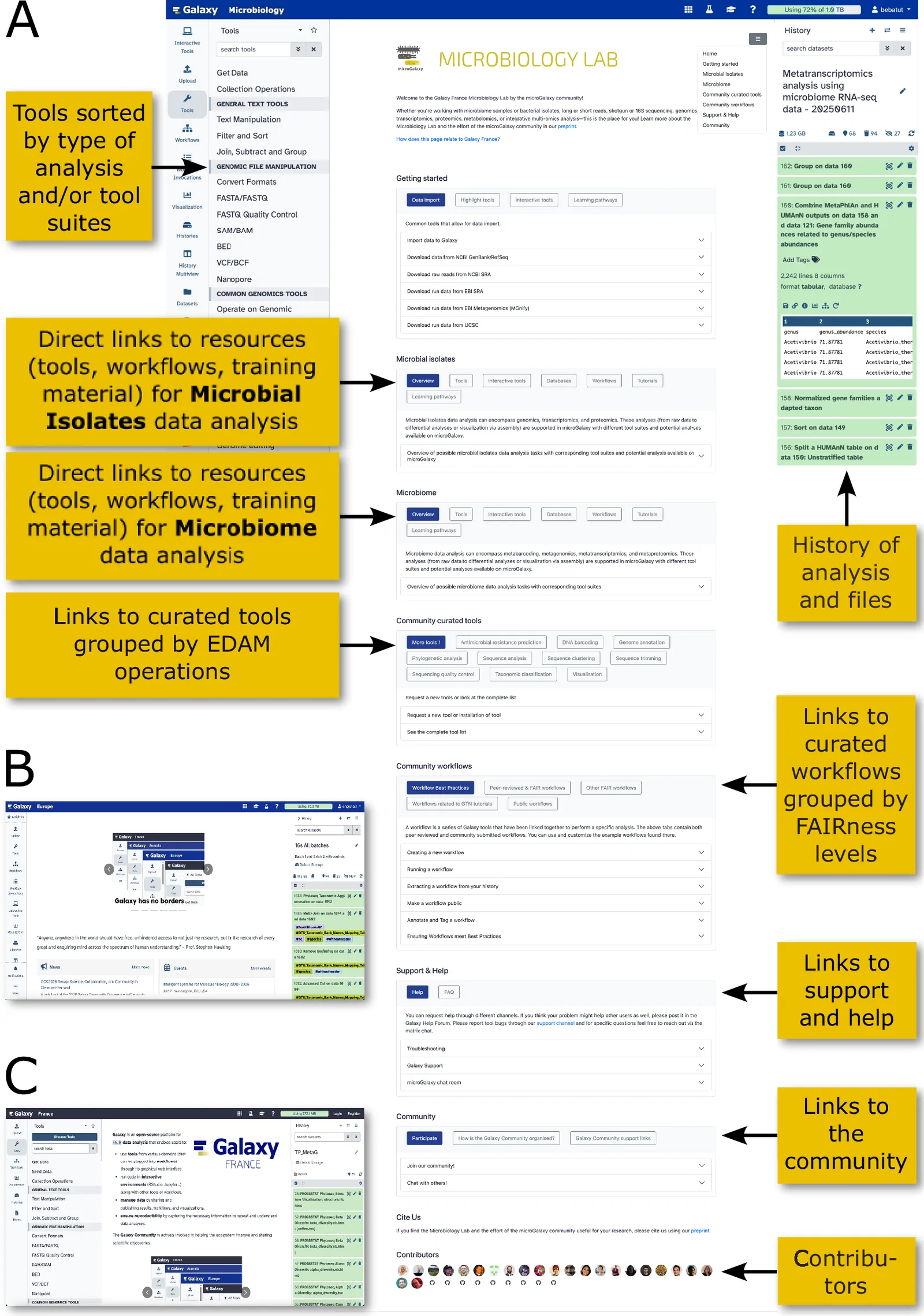
Microbiology Galaxy Lab (MGL) interface in comparison with default Galaxy interfaces on the UseGalaxy server. **A**) The MGL interface is composed of a tool panel on the left-hand side, where tools are organised by type of analysis and tool suites. The main panel, in the middle, includes structured entry points for different data types (Microbial Isolates and Microbiome), offering grouped direct access to tools, workflows, and training material. Community-curated tools and workflows are also accessible, organised by EDAM topics/operations for the tools and FAIRness levels for the workflows. The main panel also includes quick access to help, support, and the microGalaxy community. The history panel, on the right-hand side, displays analysis steps and datasets in chronological order. **B**, **C** The default Galaxy home page is used in Galaxy Europe and Galaxy France, respectively.

In response, the Galaxy platform^18^, an open, web-based, widely adopted, FAIR-compliant platform for reproducible research, was developed to address data-intensive biomedical analysis needs. To tailor Galaxy for microbial research, the microGalaxy community (https://galaxyproject.org/community/sig/microbial) was formed, a Special Interest Group (**SIG**) comprising microbiological research practitioners, including microbiologists and bioinformaticians. This community surveyed the microbiology community (Galaxy users and beyond), revealing primary use of the platform in bacterial genomics and metagenomics, with growing interest in functional analysis, gene identification, and genome assembly (Supplementary Data 2). The survey identified emerging needs in metaproteomics and antimicrobial resistance profiling, alongside challenges like limited user experience and technical hurdles. The findings led to the Microbiology Galaxy Lab (**MGL**) creation, an open-source platform unifying community-curated tools, workflows, and training for reproducible microbiological data analysis. Freely accessible on multiple public and free Galaxy servers (microbiology.usegalaxy.org, .eu, .org.au, .fr), the MGL offers a dedicated interface where highlighted resources integrate seamlessly with Galaxy for direct execution (Fig. 1).

The MGL builds on Galaxy to address the microbial bioinformatics challenges by providing: (i) access to numerous community-curated software tools and workflows, (ii) a user-friendly interface, (iii) a powerful workflow management system including a graphical editor, (iv) free access to public computational resources, (v) integration with training resources for all expertise levels users, and (vi) automation, integration, and advanced data management via command-line tools and a comprehensive Application Programming Interface (**API**). By leveraging these features, the MGL supports microbiologists, bioinformaticians, and practitioners in overcoming data and computational barriers. The MGL enhances health/disease and ecosystem/biodiversity research by integrating microbial and phenotypic data analyses (e.g., antibiotic resistance profiles, ecological traits). This fosters interdisciplinary research and standardised, reproducible approaches, advancing microbiological data analysis and our understanding of microbial and ecosystem diversity in health and global environmental change.

## Results

### Galaxy adoption for microbial research: community trends and insights

**Fig. 2:**
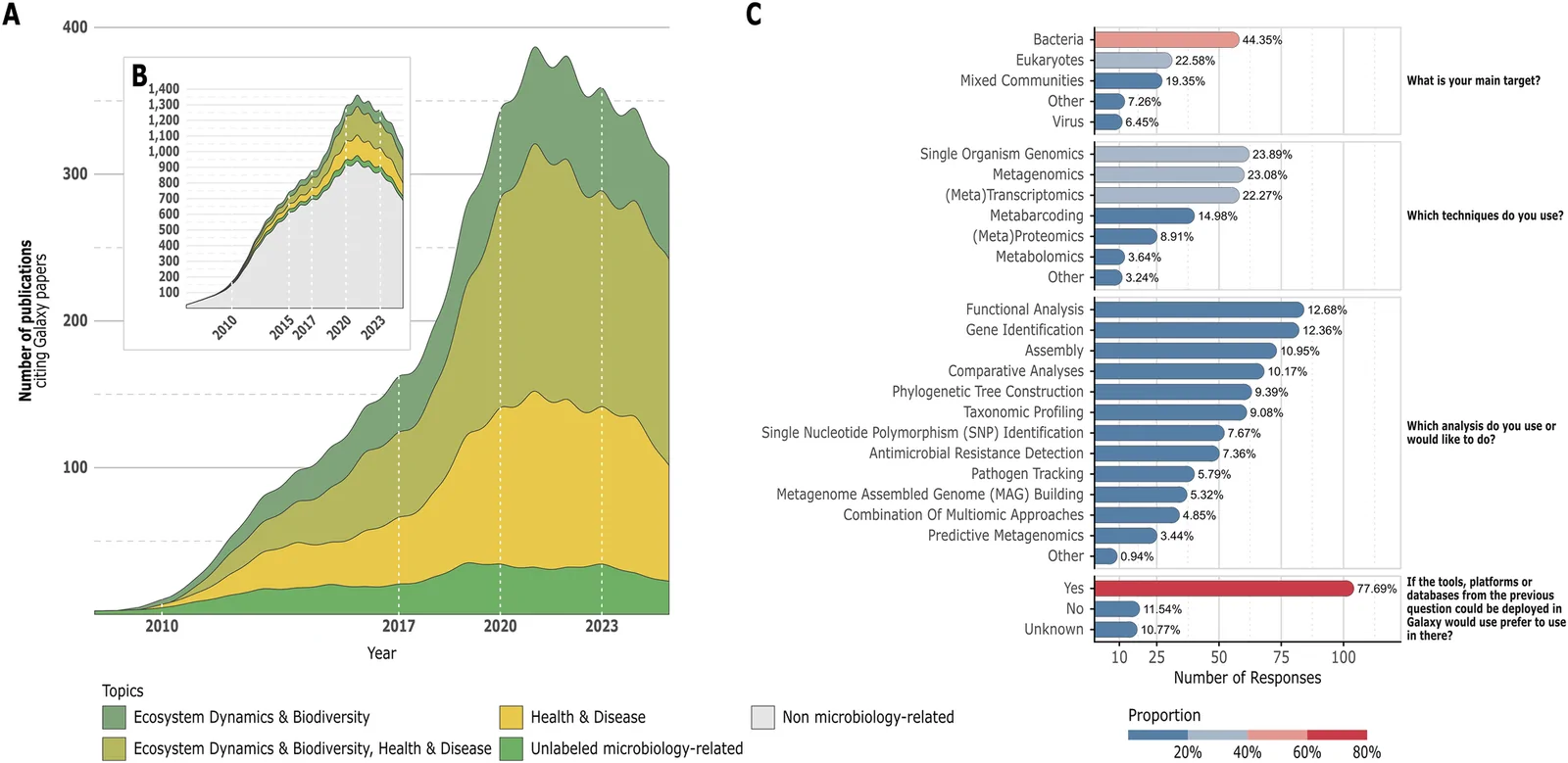
Citation trends in Galaxy publications and survey results from the microbiology research community. (March–September 2023). **A** Annual publication trends show the total number of microbiology-related citations divided into health and disease (yellow), ecosystem dynamics and biodiversity (green), both (olive yellow), and unlabelled (light green). **B** Annual publication trends show the total number of non-microbiology-related citations (grey) alongside those specific to microbial research, shown in (**A**). **C** Anonymous responses highlighting the main research targets, techniques, and analyses used or desired by microbiology researchers. The figure also reports challenges faced by users who rarely or never use Galaxy and preferences regarding tool deployment in Galaxy. Percentages were computed independently for each question based on the total number of responses available for that question. Additionally, some questions, such as “*Which analyses do you use or would you like to do?*” allowed multiple responses, meaning percentages may sum to more than 100%.

To demonstrate the value of user-friendly implementations for complex computational workflows, a dual-pronged analysis was conducted: a text-mining review of published literature using Galaxy for microbiology and a survey of microbiology data analysis practitioners, including researchers/scientists and bioinformaticians. Our analysis reveals a consistent upward trend in Galaxy utilisation from 2006 to 2025, with a pronounced increase in microbiology applications. In total, 10,991 publications relied on the Galaxy platform for their analysis (Supplementary Data 3), with 3,067 focusing on microbiology (Fig. 2A, Supplementary Data 4). Among these, 883 studies (28.8%) addressed health and disease applications, 617 (20.1%) focused on ecosystems and biodiversity, and 1,184 publications (38.6%) spanned both domains, highlighting the interdisciplinary nature of microbial research (Supplementary Fig. 1). The most prevalent applications were (meta)genomics, followed by metabarcoding and metatranscriptomics, reflecting the complexity and diversity of microbiological data. Key analytical functions included taxonomic classification, functional analysis, and antimicrobial resistance (**AMR**) gene profiling, with target taxonomic groups ranging from bacteria and pathogens to microbiomes and viruses (Supplementary Fig. 1). While manual validation confirmed the overall trends, the Technical Targets classification showed lower agreement (F1 = 33.8%, recall = 23.4%, Supplementary Note 1), so interpretations involving this dimension should be treated with caution.

The survey (Supplementary Method 1) aimed to assess current microbiology applications and identify areas for improvement. It provides a detailed view of the priorities and practices of microbial bioinformaticians and researchers. Results show a clear focus on multidisciplinary methodologies, with bacteria as the main target organism for 44.4% of respondents (Fig. 2C, Supplementary Data 2). The top three techniques were single-organism genomics (23.9%), metagenomics (23.1%), and (meta)transcriptomics (22.3%), indicating diverse yet clustered popular approaches. For computational tasks, functional analysis (12.7%) was the most frequently used or desired, followed by gene identification (12.4%) and genome assembly (11.0%).

The most significant finding regarding microbiology computational analysis was that the primary barrier to its adoption was a lack of experience (51.9%) and technical difficulties (25.9%), rather than a lack of utility (Supplementary Data 2). This is reinforced by the fact that 77.7% of respondents would prefer Galaxy if it integrated the tools and databases they needed (Fig. 2C). This presents a clear opportunity for a focused platform leveraging microbiology tools to enhance the user experience. Additionally, the survey revealed that Galaxy’s user base is currently concentrated in Europe (31.7%) and Asia (27.9%, Supplementary Data 2**)**, suggesting its greatest transformative potential lies in serving researchers in developing countries. A microbiology-focused Galaxy platform, like the Microbiology Galaxy Lab (MGL), would enable scientists to analyse their datasets using computationally complex methods, overcoming local infrastructure limitations and barriers to promote scientific self-reliance.

### A Microbiology-focused Galaxy Lab for FAIR and reproducible analyses

The Microbiology Galaxy Lab (MGL) is an extension of existing web-accessible Galaxy servers, providing a custom landing page and a tailored toolbox accessible through a subdomain **(**Fig. 1). This enables users to access domain-specific tools, workflows, and resources within the Galaxy environment^18^. The MGL features a curated interface for microbiology research, allowing users to run individual tools or build complex workflows while maintaining full provenance tracking to ensure reproducibility. Galaxy is a platform with representation across multiple countries and regions, including Europe, America, and Australia. The MGL is hosted on equivalent Galaxy servers in globally distinct regions (e.g., microbiology.usegalaxy.org, .eu, .org.au, .fr), offering free access to storage and computational resources up to server-specific limits. Additionally, local deployment of the MGL is possible via a Docker container (https://github.com/usegalaxy-eu/docker-galaxy-microbiology-lab). This setup allows users worldwide to perform sophisticated analyses regardless of local infrastructure. Users can upload data through local files, bulk data transfer, or commercial clouds, or fetch it automatically from sources such as **UCSC** Genome Browser database^19^, **NCBI** Sequence Read Archive (**SRA**)^20^, **EMBL-EBI** European Nucleotide Archive (**ENA**)^21^, and MGnify^22^. With automatic dataset retrieval and reuse, combined with Galaxy’s capability to export workflows as **RO‑Crate**^23^ packages, the MGL ensures analysis reproducibility and machine-readability in line with FAIR guidelines. By building on Galaxy’s foundation, the MGL supports FAIR and reproducible microbiological data analyses (Table 2).

**Table 2:** Support for FAIR and reproducible microbiological data analyses in the Microbiology Galaxy Lab (MGL)

FAIR Principles	Implementation in *Galaxy* and MGL	
**Findable**	Metadata and Tagging	*Galaxy allows users to annotate datasets, workflows, and analyses with rich metadata (e.g., tags, help markdown, annotations, descriptions)*. In MGL, community-curated tools, workflows, and training materials are aggregated inside the Galaxy Codex^95^ and annotated using the EDAM ontology^72^ by the microGalaxy community (implemented and visible in tools and workflow metadata).
	Persistent Identifiers (**PIDs**)	Community-curated MGL workflows are associated with **DOI**s (Digital Object Identifiers) via *Galaxy integration with WorkflowHub*^30^, ensuring they can be uniquely identified and cited. MGL *(as any Galaxy) users can also export histories and entire data analysis provenance to Zenodo*^118^ *and obtain a DOI to store them as a reference for other users*.
	Public Repositories	Community-curated MGL workflows are published on WorkflowHub and Dockstore platforms, and the tutorials on ELIXIR **TeSS**^115^, making them indexed and searchable, increasing their visibility. *Galaxy can interface with* ***LIMS**** and Research Data Management repositories (e.g., RSpace*^119^ *or InvenioRDM*^120^*), Dataverse (e.g., Harvard Dataverse Repository), and many more data repositories, easing data access*.
**Accessible**	Open Access	*Galaxy is open-source and freely available, allowing anyone to install and use it. Public Galaxy servers (e.g., usegalaxy.org, usegalaxy.eu) provide free access to computational resources for researchers worldwide*.
		Tools, community-curated Workflows, and tutorials in MGL, as well as sources for the MGL interface, the catalogue, and the Docker image, are open-sourced and freely available. MGL can be easily installed as a subdomain of any Galaxy server or deployed on its own using the dedicated Docker image.
	Data Sharing	*Galaxy enables the sharing of datasets, workflows, histories, visualisations, and pages via shareable links or by publishing them to public repositories. Access permissions can be finely controlled (e.g., private, shared with collaborators, or fully public)*. These sharing mechanisms are fully enabled and used in MGL.
	Cloud and Containerisation	*Galaxy supports deployment in cloud environments (e.g., Kubernetes) and containerisation (e.g., Docker, Singularity), making it accessible across diverse infrastructures*. A Docker image with the MGL is available for deployment.
**Interoperable**	Adherence to community standards	*Galaxy is an ELIXIR Recommended Interoperability Resource (****RIR****)*^114^ *and actively supports Global Alliance for Genomics and Health (****GA4GH****) standards and APIs (*https://galaxyproject.org/ga4gh*)*. The MGL inherits and uses these standards through Galaxy, without custom reimplementation.
	Standardised Formats	*Galaxy supports and enforces the usage of a wide range of standard data formats (e.g., FASTQ, BAM, VCF, Tabular) and encourages the use of community standards (e.g., GA4GH for genomics)*. The MGL relies on these standard formats, ensuring compatibility with external tools and platforms.
	APIs and Interface	*Galaxy provides REST APIs (OpenAPI annotated) and integrates with GA4GH APIs, including: (i) Data Repository Service (****DRS****) API as both a producer and consumer, facilitating easy exchange of genomic data with other DRS-compliant systems; (ii) Beacon API, enabling researchers to discover and query genomic variants across datasets; (iii) Task Execution Service (****TES****) API leveraged by Galaxy’s Pulsar for large-scale genomic analyses, ensuring standardised and interoperable task execution; (iv) Tool Registry Service (****TRS****) API to access and share workflows via platforms like Dockstore and WorkflowHub, making it easy for users to find, import, and run community-developed workflows*. The MGL benefits from all these functionalities.
	Integration with other tools and Platforms	*Galaxy supports integration with popular data science environments such as Jupyter Notebooks, RStudio, and R-Shiny apps*. On top of these functionalities, MGL provides microbiome-specific interactive tools, including phyloseq^28^, Pavian^26^, and Phinch^27^, for exploration and visualisation of microbiome and metagenomics data. MGL also supports integration with MGnify^22^, enabling seamless data exchange and interoperability with external systems.
	Workflow Standard	*Galaxy workflows can be exported in RO-Crate format. Galaxy also integrates with Dockstore and WorkflowHub, allowing users to search, import, and publish workflows directly from the Galaxy interface*.
**Reusable**	Reproducibility	*Galaxy captures the entire provenance (tools, versions, parameters)*. This functionality is fully available and used in MGL, enabling exact re-execution of microbiological analyses.
	Versioning	Tools, community-curated workflows, and tutorials in MGL are stored on public git repositories and versioned, allowing users to track changes and revert to previous versions if needed.
	Documentation	*Galaxy encourages detailed documentation for workflows and tools, including step-by-step guides, a best practice wizard, and tutorials via the Galaxy Training Network (GTN)*^64^. The MGL offers 39 tutorials associated with 117 workflows.
	Community and Collaboration	*The Galaxy community thrives on collaboration, actively sharing best practices, workflows, and tools, fostering reuse and collaboration. Each resource (tools, workflows, tutorials) prominently lists its contributors, ensuring transparency and proper attribution. The GTN offers dedicated contributor pages, where individuals and organisations are recognised for their contributions. These pages highlight the collective effort behind Galaxy’s growth and serve as a testament to the community’s collaborative spirit. Platforms like Galaxy Europe and Galaxy Australia provide additional support and resources*. Within this ecosystem, the MGL community actively curates, maintains, and extends microbiology-focused tools, workflows, and training materials, contributing domain expertise while benefiting from and reinforcing the broader Galaxy community. Contributors to the MGL are cited at the bottom.

Features in *Italic* are provided by the core Galaxy platform and are directly available within MGL; non-italic features correspond to MGL-specific curation or community-driven implementations.

### Community-curated and ready-to-use tools and workflows on MGL

**Fig. 3:**
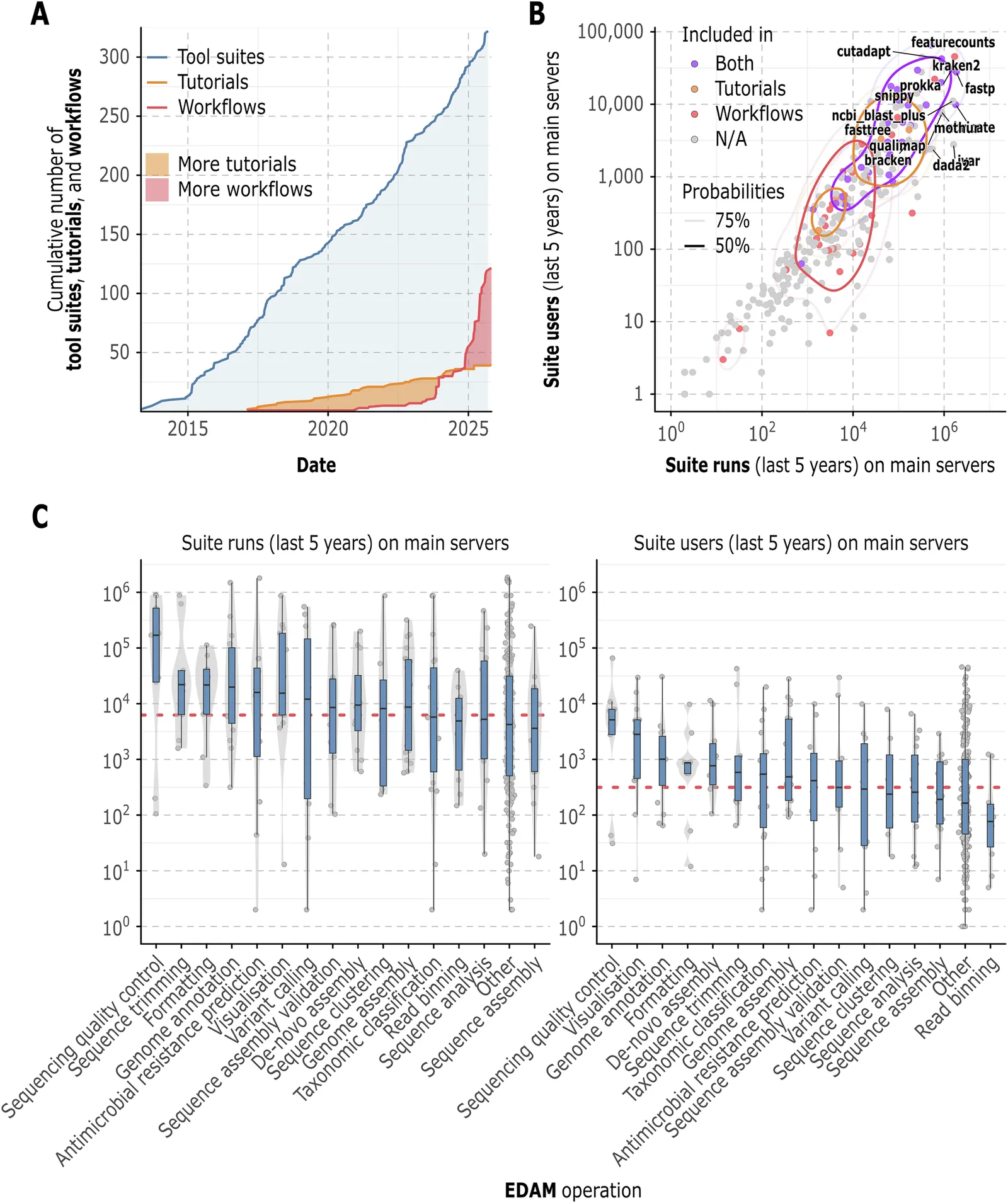
Growth and usability of microbiology-related tool suites, training materials/tutorials, and workflows within the Galaxy ecosystem that are included in the MGL. **A** Cumulative number of microbiology-related tool suites (blue), tutorials (orange), and workflows (red) added to Galaxy over time, based on the date of the first commit. **B** Scatter plot showing the usage (number of runs and number of users) of microbiology-related tool suites over the past five years across all Galaxy main servers. Tool suites are depicted with different colours depending on whether they are included in tutorials (orange), workflows (red), or both (purple). Distribution across suite runs and suite users was displayed in 2D-density estimation lines at 50% (darker colour) and 75% (lighter colour) probabilities. **C** Box plot showing the usage of microbiology-related tool suites over the past five years across all Galaxy main servers, categorised by their EDAM operations. The red dashed line depicts the average number of suite runs and users for each panel, respectively.

Over the past five years, tools included in the MGL have supported over 25 million analyses, either as standalone tool execution or within workflows, highlighting their broad utility in microbial research (Fig. 3A, Supplementary Data 5). Metrics on tool usage show that tools incorporated into workflows and tutorials are used more frequently by the community (Fig. 3B), emphasising the importance of workflow integration and training-based documentation for improving accessibility and adoption. The assignment of tools to EDAM, an ontology defining core concepts in scientific data analysis and management, demonstrates that the toolset covers a wide range of key computational functions. Some of these functions have been executed hundreds of thousands of times by several thousand users (Fig. 3C). The high number of tool runs often results from their integration into workflows, which are executed by automated pipelines or used for high-throughput analyses across various Galaxy servers.

**Fig. 4:**
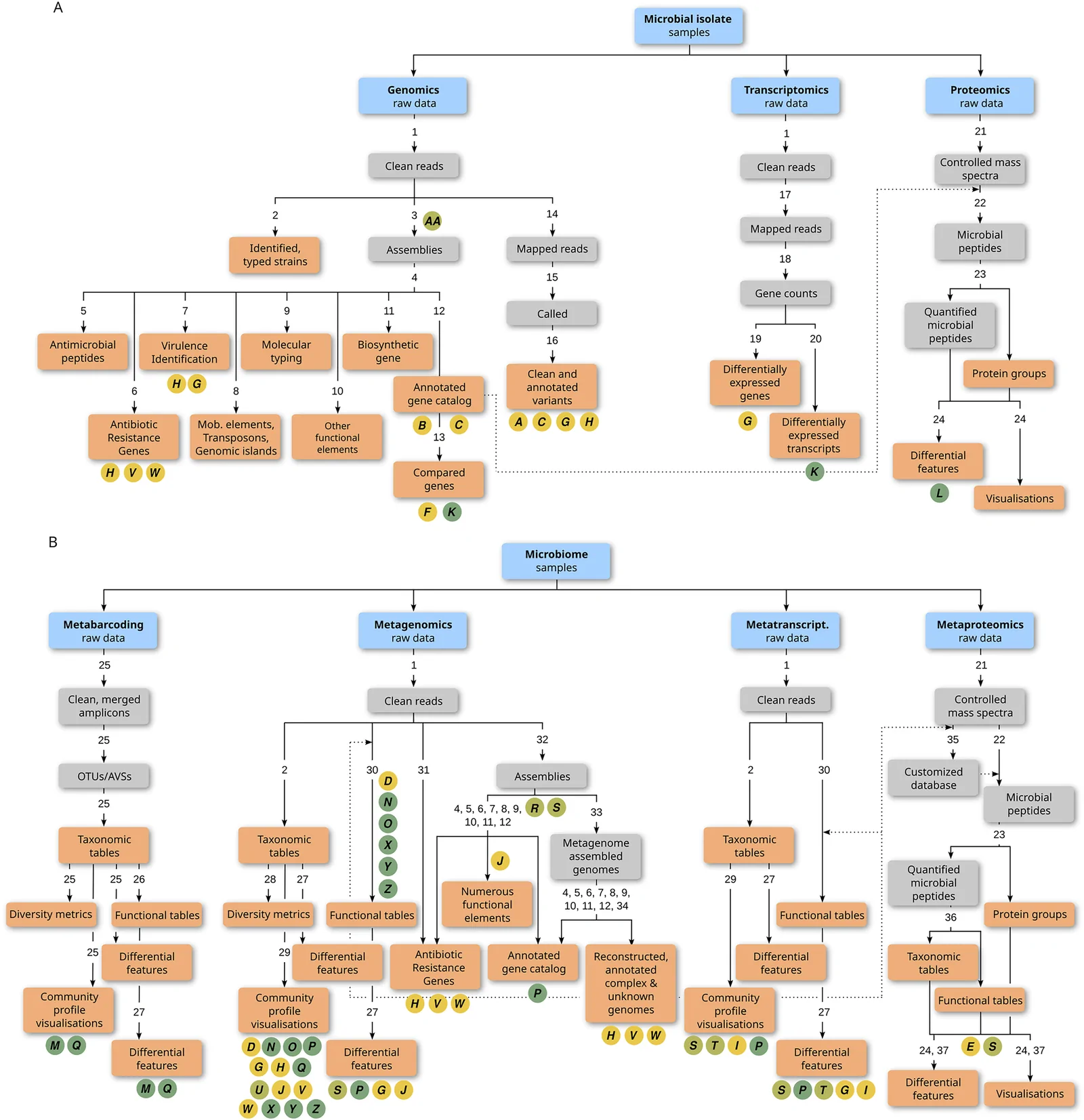
Overview of microbiological data analysis tasks with corresponding tool suites and potential analyses available on the MGL. Analysis tasks, categorised by data type, for **A** microbial isolate samples, including genomics, transcriptomics, and proteomics, and **B** microbiome samples, encompassing metabarcoding, metagenomics, metatranscriptomics, and metaproteomics. Each task progresses from raw data (blue) to intermediate (grey) and final outputs (coral). Numbers on the branches correspond to tool suites that can be used for generating the outputs below, detailed in Supplementary Data 6. If numbers appear before a node, all outputs can be generated by the corresponding tool suites. Circled letters indicate use cases, as listed in Supplementary Data 8, colour-coded by domain: health and disease (yellow), ecosystem dynamics and biodiversity (green), and for both (olive yellow).

The MGL offers a powerful and diverse collection of tool suites designed for large-scale microbiological data analysis. It includes 884 community-curated tools sourced from 319 tool suites within the broader Galaxy ecosystem, tailored for a wide range of microbiology applications (Fig. 4, Supplementary Data 5, Supplementary Data 6, Supplementary Fig. 2). Some tool suites even include complete frameworks, such as QIIME 2^24^, whose community selected Galaxy as the platform to provide front-end access to their resources. Through these tools, users can access over 170 integrated reference genomes and 11 terabytes of reference data, shared and delivered via CernVM-FS^25^, a distributed network file system optimised for scientific software and data. These reference data support essential microbial bioinformatics tasks like taxonomic classification and functional annotation. The platform also supports interactive tools that enable user-driven microbiological data exploration. Examples include Pavian^26^, Phinch^27^, Shiny Phyloseq^28^, and Apollo^29^, which facilitate real-time inspection and curation within the platform. Additionally, general-purpose tools such as Jupyter Notebooks (RRID:SCR_018315) and RStudio (RRID:SCR_000432) enhance interactivity and reproducibility, supporting modular and shareable analyses. Complementing its toolset, the MGL hosts 117 curated, ready-to-use workflows, whose counts have dramatically increased in the past 2 years **(**Fig. 3A, Supplementary Data 7, Supplementary Fig. 3), streamlining complex analyses into reproducible, user-friendly processes. These workflows reflect a broad consensus within the microGalaxy community and are developed by domain experts in microbiology and bioinformatics. Among these, 42 workflows are published in public repositories like **WorkflowHub**^30^ and **Dockstore**^31^ and can be directly executed within the MGL or installed on any Galaxy instance. The rest are shared across Galaxy servers under the *microgalaxy* tag, allowing direct discovery and reuse. To maximise the use of these workflows and facilitate customisation, the platform’s graphical interface is a key strength, enabling interactive creation, customisation, and management of workflows. This significantly lowers the technical barrier for designing and executing complex analyses. The integration of the MGnify amplicon pipeline exemplifies the MGL’s ability to support reproducible yet customisable analyses, allowing anyone to reproduce MGnify complex analyses while retaining the flexibility to modify tool and database versions, a feature absent in the original implementation. Although the Galaxy MGnify pipeline may show slight result variations because of the non-deterministic nature of tools like MAPseq^32^, users benefit from the platform’s broader ecosystem and integrations. This example illustrates workflow portability and reproducibility within the Galaxy environment while preserving flexibility for user-driven adaptation.

To support users with diverse bioinformatics expertise, the microbiology workflows provided in MGL are utilised and interpreted in training materials and tutorials, as detailed in the following subsection (*Resources backed by comprehensive training support*). The tools, workflows, and associated training materials are continuously maintained, regularly updated, and expanded to meet emerging needs in microbial research (Fig. 3A).

### Examples of MGL applications in health, disease, and ecosystem research

**Fig. 5:**
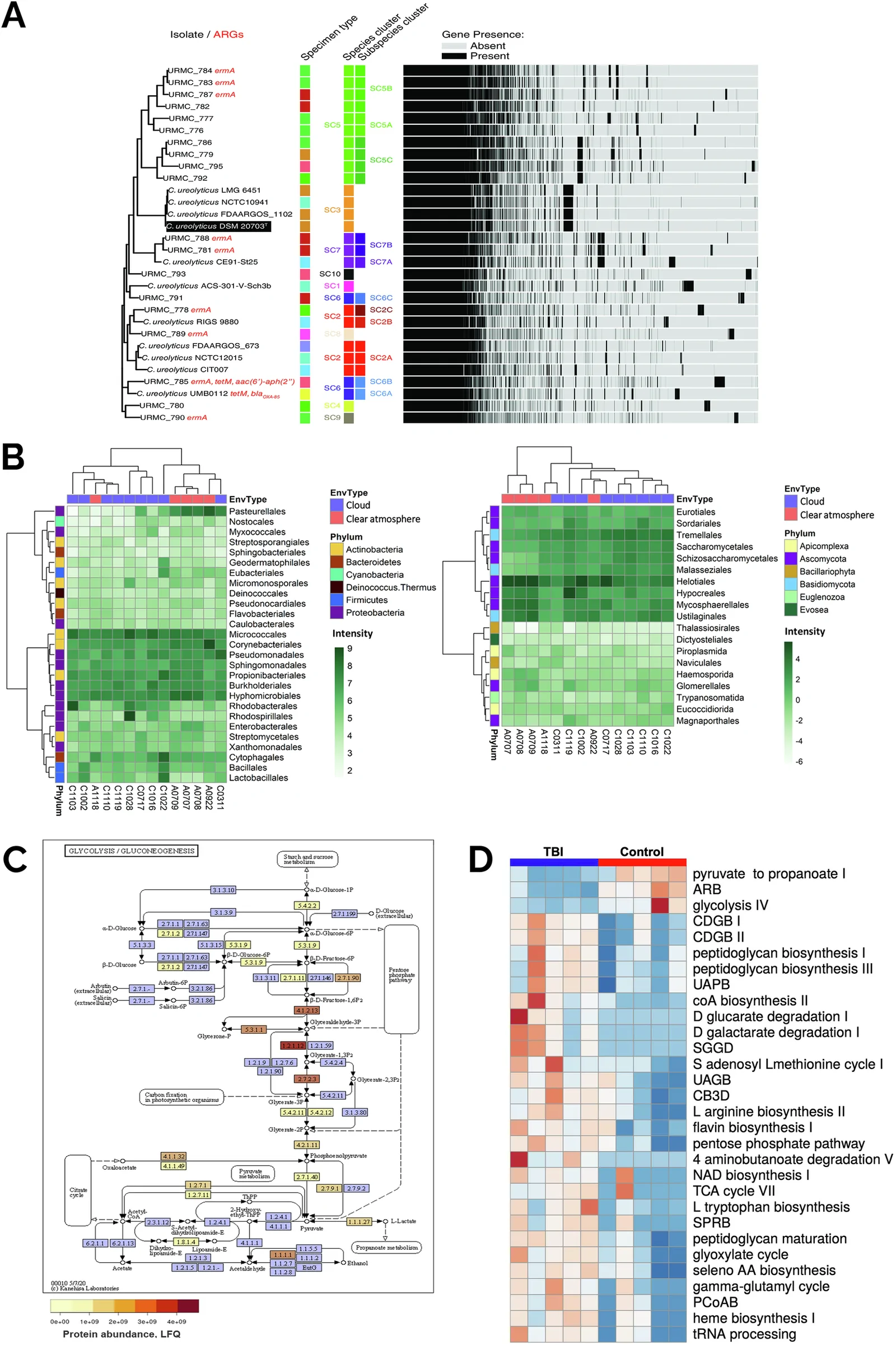
Applications of MGL tools and workflows in health, disease, and ecosystem research. **A** Core-genome phylogeny of *Campylobacter ureolyticus* isolates calculated from a concatenated core-genome alignment of clinical isolates and publicly available genomes (BV-BRC database), generated with Roary^96^ from Prokka^97^-annotated genomes. Antimicrobial resistance genes and plasmid markers were identified using ABRicate^98^ and StarAMR^99^ on Galaxy. The phylogenetic tree, annotated with species/subspecies clusters, specimen type, antimicrobial resistance profiles, and gene presence–absence patterns, was visualised in R. The analysis combines Galaxy-based genomic annotation and resistance profiling with downstream statistical visualisation, which can be fully reproduced within Galaxy environments (Derived from Fig. 1C in Maki et al., 2023^100^, CC BY 4.0 license). **B** Comparative distribution of dominant bacterial and eukaryotic orders in cloudy versus clear-atmosphere metagenomes, with hierarchical clustering (Derived from Fig. 1 in Péguilhan et al., 2025^58^, CC BY 4.0 license). The figure corresponds to clustered heatmaps generated using Galaxy statistical tools or integrated R/Jupyter environments (e.g., ComplexHeatmap^101^). **C** Glycolysis/Gluconeogenesis pathway of a *Tissierellia*-class bacterium, built as a Metagenomics Assembled Genome from metagenomics data and annotated with metaproteomic abundance (Derived from Fig. 3 in Schiml et al., 2023^46^, CC BY 4.0 license). A similar figure can be created using pathway analysis tools available in Galaxy (e.g., KEGG/Pathview^102^ integration). **D** Heatmap of 30 differentially expressed biochemical pathways in traumatic brain injury (TBI) versus control faecal metatranscriptomes (Derived from Fig. 3 in Pyles et al., 2024^103^, CC BY 4.0 license). The figure represents differential abundance visualisation generated using Galaxy statistical workflows (e.g., DESeq2^104^) and built-in heatmap modules.

Tools and workflows in the MGL offer an extensive suite of diverse methodologies **(**Fig. 4) to support a wide range of applications in health, disease, and ecosystem research (Fig. 5, Supplementary Data 8**)**.

### Health and disease

In the health and disease context, bioinformatics workflows are essential for elucidating microbial pathogenesis and host-microbe interaction mechanisms. A key application involves analysing how pathogens alter their gene composition, expression, and protein production to survive within the host environment, resist the immune system, or develop antibiotic resistance^33^. Workflows, such as metagenomic assembly and functional annotation, are critical for studying biofilm formation and cell sensing, contributing to bacterial persistence, immune evasion, and resistance to antimicrobial treatment^34^. These workflows also facilitate tracking microbial community dynamics, AMR dissemination, and the movement of mobile genetic elements (MGE) across hosts and environments, effectively supporting the One Health concept. Such computational microbiology analyses, along with use cases presented below, rely on consistent tools and standardised processing provided by the MGL to ensure that results from different patient samples remain comparable. AMR genes pose a significant threat to healthcare^33^, making comprehensive analysis of their presence and sequences in both clinical and environmental samples vital for controlling their spread and treating severe infections. With the growing volume of clinical sequencing for routine diagnostics, there is a critical need for intuitive analysis platforms accessible to non-specialists that support AMR analysis, including genotype identification and its correlation with plasmid carriage. ABRomics (https://www.abromics.fr/), a platform for AMR research and surveillance, is built on MGL with workflows developed in collaboration with the microGalaxy community. These workflows cover stages from sample sequencing quality control to taxonomic assignment, genome assembly and annotation, and AMR gene identification. Since AMR genes often spread through MGE, workflows were developed using tools like PlasmidFinder, geNomad, and plasmidSPAdes to screen genomes and identify plasmids, integrons, and insertion sequence elements. Tools and workflows in ABRomics represent options for defining AMR genotypes. However, these methodologies can generate diverse results due to varying reference databases, algorithms, or parameters. To address this, BenchAMRking^35^ was developed on top of Galaxy, including an ISO-certified AMR identification^36^ tool that generates a clinical report, making it suitable for implementation in healthcare settings. BenchAMRking highlights the need for greater standardisation in AMR research. Additionally, Galaxy-ASIST^37^ bridges the gap between genotype and phenotype by integrating antimicrobial susceptibility testing (**AST**) data with genomic AMR profiles, enabling harmonised interpretation and clinical reporting in line with global standards. The versatility of MGL also enables the study of diverse microbial taxa and resistance mechanisms. For example, Merdan et al., 2022^38^ identified in *Candida glabrata* drug-resistance mutations conferring resistance to azole and amphotericin B, while Arcari et al., 2023^39^ tracked the genotypic evolution of a *Klebsiella pneumoniae* strain under different drug treatments. AMR can also be studied in microbiome data, but this requires substantial computational power and specialised expertise to extract AMR genes and link them to their microbial hosts. To tackle this, dedicated workflows are being developed, particularly by the ABRomics team.

Another health-related challenge is pathogen detection, such as for food safety or outbreak tracking. PathoGFAIR^40^ offers end-to-end, curated Galaxy workflows for quality control, taxonomic profiling, and AMR and virulence factor identification to detect and track pathogens from high-throughput sequencing data. By providing standardised workflows that support reproducibility, PathoGFAIR facilitates interoperable analyses and contributes to harmonisation efforts in pathogen surveillance and outbreak investigations. In clinical metaproteomics, a powerful approach in microbiome research to offer insights into host-microbe interactions underlying human disease, challenges persist in characterising low-abundance microbial proteins amid host proteins. Researchers also face difficulties in handling large protein sequence databases for peptide and protein identification from mass spectrometry data, as well as in performing taxonomic and functional assignments and statistical analysis to identify differentially abundant peptides. Several Galaxy workflows tackle these challenges, supporting microbiome-based studies on COVID-19 (e.g., nasopharyngeal swab^41^ or wastewater surveillance), chronic respiratory conditions like cystic fibrosis^42^, and biomarker discovery in diseases such as ovarian cancer^43^.

The MGL supports the One Health framework through specialised tools like Kraken^44^, pipelines like PathoGFAIR, or platforms built on top of Galaxy like ABRomics and BenchAMRking, enabling researchers to track pathogen spread and AMR dissemination. Real-world efficacy of MGL in One Health is demonstrated by its application in wastewater-based viral surveillance to identify cryptic lineages^45^, standardised AMR monitoring across clinical and environmental sectors^37^, and integrative meta-omic ecosystem analyses^46^. The application of machine learning and artificial intelligence (**AI**) to microbiology research is a growing area of interest, successfully integrated into Galaxy. For example, Cumbo et al., 2025^47^ used AI-driven tools to identify potential microbial biomarkers for colorectal cancer by analysing public metagenomic samples from patients in a case/control scenario. To classify unknown microbes, especially viruses, the MetaSBT framework^48^ uses Sequence Bloom Trees, identifying multitudes of novel viral species clusters. The public availability via MGL ensures broad accessibility, supporting both research and routine public health applications within a One Health paradigm.

### Ecosystem dynamics and biodiversity

Understanding microbial ecosystem dynamics is fundamental to the planet’s survival, as it drives critical processes such as nutrient cycling, carbon sequestration, and decomposition^49,50^. Microbial taxonomic classification is primarily achieved through metabarcoding, metagenomics, and whole-genome sequencing of isolated strains. However, this presents major challenges, including the large volume of shotgun sequencing data, assembling genomes from mixed-organism read pools, computational complexity, and the need for deep, accurate data to resolve strain-level differences^51,52^. Overcoming these hurdles requires integrated sequencing analysis platforms with high computational capacity for large-scale data analysis. Such infrastructure serves as a powerful tool, helping researchers achieve their research objectives efficiently and easily.

The MGL includes workflows for short and/or long-read sequencing to classify ecosystem members and decipher community dynamics. Long-read sequencing combined with short reads was employed to identify *Trichoderma* species strains, proving suitable for hybrid assemblies and correct classification even at the strain level^53^. Microbiome studies often depend on 16S rRNA amplicon sequencing. For instance, amplicon data were used to explore root microbiomes, and species classification tools available in the MGL allowed for the identification of bacterial species essential for promoting plant growth^54^. Via the MGL, scientists can classify taxonomic species using state-of-the-art tools either as standalone or inside predefined but customisable workflows. For example, they can use the MGnify^22^ amplicon analysis pipeline adapted to Galaxy in collaboration with EMBL-EBI.

MGL not only makes complex analyses accessible to researchers but also to citizen scientists, supporting participatory microbiome classification and education. For example, the BeerDEcoded project by the Street Science Community (https://streetscience.community/) used MGL’s user-friendly interface to engage diverse participants, including high school students, in analysing and exploring the classification of beer microorganisms while learning about open science principles. Similarly, the BioDIGS project, part of the Genomic Data Science Community Network (GDSCN)^55^, empowers scientists from underserved institutions across the United States to investigate microbial life in their local environments^56^. Additionally, the non-profit organisation, SPUN, leverages MGL to map mycorrhizal fungal communities on a global scale.

To understand the functioning of microbiomes, functional annotation aims to define the role of constituent genes. However, accurate functional assignment remains a major challenge. Standardised workflows create comprehensive functional profiles by automating gene prediction searching against multiple reference databases, enabling trait discovery that pinpoints novel microbial enzymes and metabolic pathways^57^.

To confirm predicted functions and traits, genetic potential (metagenomics) must be linked to real-time activity (metatranscriptomics, metaproteomics). The MGL provides workflows and tools for such multi-omics integration. A compelling example of this is the work by Péguilhan et al., 2025^58^, who combined metagenomics and metatranscriptomic data to reveal microbes functioning in different atmospheric compartments (Fig. 4B). By building and using an annotated gene catalogue to control for variability, a differential expression analysis revealed key metabolic and stress responses triggered by the cloud environment, which affects both atmospheric chemistry and ecosystems receiving precipitation. Another study of the North Atlantic Ocean microbiome^59^ underscored the importance of robust and reproducible workflows in environmental metaproteomics for accurate taxonomic and functional analyses of mass spectrometry data. In this context, the resources in the MGL provide clear guidelines and workflows for proteomics. Schiml et al., 2023^46^ successfully demonstrated a comprehensive assessment of a community’s functional potential through the seamless multi-omic integration of metagenomic, metatranscriptomic, and metaproteomic data (Fig. 4C). Their sophisticated strategy involved first recovering high-quality metagenome-assembled genomes (**MAGs**) and then using the predicted genes to construct custom databases for the subsequent protein and mRNA identification. Finally, metabolomics can complement these analyses by reflecting real-time cell function. The platform ecosystem is fully equipped to support such integrated analyses^60–62^.

### Resources backed by comprehensive training support

MGL enables users to execute complex analyses through precise self-guided training, fostering understanding, learning, and improved data interpretation. It offers 39 tutorials, 17 videos (16 hours total), and 4 structured learning pathways for microbiology analyses hosted on the Galaxy Training Network (**GTN**)^63,64^ (Supplementary Fig. 4, Supplementary Data 9). These resources cover various topics ranging from basic sequence analysis to advanced metaproteomics or metagenomics assembly (Supplementary Fig. 4) and are regularly updated to align with the latest tools and methodologies. Since 2016, there has been a steady cumulative increase in GTN tutorials with a microbiology focus, with ongoing growth of available materials (Fig. 3A). Integrating these materials into the MGL enhances user accessibility, experience, and confidence in conducting complex microbiology analyses.

Beyond self-guided resources, training events play a critical role in skill development. The microGalaxy community, supporting the MGL, actively supports and leads diverse training efforts. Over the past five years, it has conducted 40 training events (Supplementary Data 10), often supported by the Galaxy Training Infrastructure as a Service (TIaaS) framework^65^, which provides dedicated computing resources for efficient data processing during training sessions. For example, the 2021 *Analysis of Functions Expressed by Microbiomes* workshop^66^, co-hosted by CSIR-IMTech (Chandigarh, India) and the Galaxy-P team (MN, USA), introduced and trained 37 participants in microbiome functional analysis while encouraging collaboration and knowledge sharing to promote microbial research impact.

The microGalaxy community also actively supports the Galaxy Training Academy (formerly Galaxy Smörgåsbord), a large-scale global online learning event organised by the Galaxy community. Since 2021, this annual event has attracted over 10,000 participants, offering remote, asynchronous, video-based training with community support on Slack. In 2024, the microGalaxy community offered two dedicated tracks in the annual Training Academy event: *Microbiome* and *Bacterial Genomics*, where 66.7% of over 3000 registrants selected at least one of these tracks. In 2025, the *Microbiome* track was offered again, attracting 56.3% of a similarly large registrant base. This model has also been used for specialised training, such as *Mycobacterium tuberculosis* genomic analysis^67^, reaching broad participation from countries in the Global South, where tuberculosis is a major health concern. These global events establish a strong foundation for correct workflow implementation, tool selection, and parameter tuning in the platform.

Beyond training, MGL users benefit from strong support via the Galaxy Help forum (https://help.galaxyproject.org/) and a dedicated channel (https://matrix.to/#/#galaxyproject_microGalaxy:gitter.im) on the open communication network Matrix.

### Robust community engagement

The microGalaxy community, which developed the MGL, was launched in 2021 as a Special Interest Group (**SIG**) within the broader Galaxy community (https://galaxyproject.org/community/sig/). SIGs are formed by dedicated individuals across laboratories and institutes to collaborate, share resources, support one another, and advocate around a common theme. The microGalaxy community fosters a collaborative atmosphere and welcomes anyone interested in Galaxy-based microbiological research. No formal membership in the microGalaxy community is required to contribute to or benefit from the open Galaxy infrastructure for microbiological research. It is currently coordinated by Bérénice Batut and Paul Zierep, who organise regular meetings, working groups, and events to coordinate efforts. Hackathons, workshops, and meetings drive workflow development, tool improvements, and knowledge sharing. The community has led two **ELIXIR**^68^ BioHackathon projects^69^ and a dedicated microGalaxy hackathon^70^ to build the Galaxy **CoDex**, the catalogue of Galaxy tools, workflows, and tutorials with their metadata^71^. These efforts enhanced tool and tutorial annotations using controlled vocabularies like the EDAM Ontology^72^ (see *Resource aggregation and annotation* Section in Materials and Methods). In 2025, another hackathon focused on integrating workflows into the Intergalactic Workflow Commission (**IWC**) (https://github.com/galaxyproject/iwc) to further expand platform capabilities.

## Discussion

The Microbiology Galaxy Lab (MGL) is a structured, domain-specific layer built on the Galaxy platform, leveraging its mature infrastructure for workflow orchestration, provenance tracking, interoperability, and scalability, while addressing microbiology-specific challenges. The MGL emphasises state-of-the-art, version-pinned, and review-based workflows to ensure reproducibility and sustainability over time. While the Galaxy CoDex serves as the central catalogue for community-curated resources, the Galaxy Labs Engine ensures their consistent rendering across Galaxy servers where the MGL is hosted. These frameworks guarantee continuous updates and maintainability.

Supported by the microGalaxy community, the MGL provides a comprehensive, freely available platform (microbiology.usegalaxy.org, .eu, .org.au, .fr) for FAIR microbiology analysis. To facilitate advanced research, the MGL integrates 319 analytical tool suites and 117 computational workflows, with access to both public and local reference databases. The platform is supported by a rich repository of training resources, including 39 tutorials, 17 videos, and 4 learning pathways, designed to support widespread adoption. As an open-source solution within the Galaxy ecosystem, the MGL provides extensive capabilities for microbiological data analysis, including a workflow manager, an accessible interface, a comprehensive suite of curated analytical and visualisation tools, and significant computational resources. This empowers scientists to perform complex analyses that might otherwise exceed their expertise or local computational capacity.

Over the past five years, the tools and workflows hosted in the MGL have been used in over 25 million analyses, demonstrating their widespread acceptance and global reach. The platform’s robust infrastructure supports analyses ranging from short-read metabarcoding, genome assembly, and taxonomic classification to complex metaproteomics, MAGs building, and AMR surveillance workflows. This extensive usage highlights the platform’s critical role in supporting both routine and advanced microbiological research.

A key feature of the MGL is its ability to respond to the evolving landscape of microbiological data-intensive research. As microbial research evolves, so do the resources for data analysis. The MGL adapts by expanding its capabilities, improving multi-omics integration, and addressing emerging challenges. Platform adaptability and sustainability are ensured through community-driven contributions and semi-automated update infrastructure. The global Galaxy community supports tool version upgrades, submission of new microbiology tools, and ongoing curation, while the microGalaxy community manages the selection, review, and deprecation of tools and workflows. Dedicated initiatives, such as community hackathons, workshops, and collaborations with infrastructure partners like ELIXIR and the Galaxy Training Network, ensure the platform continues to evolve in line with scientific and technical developments.

While platforms like MG-RAST^73^, KBase^74^, BV-BRC^75^, MGnify^22^ provide essential services, the MGL offers distinct advantages in flexibility and integration (Supplementary Data 1). MG-RAST focuses on automated annotation and comparison of genomes, but lacks modular flexibility, whereas KBase allows the addition of “apps” but has a complex interface and limited accommodation for community-contributed tools. Similarly, BV-BRC focuses on clinical pathogenomics and MGnify on large-scale microbiome archiving, but both function with predetermined, non-adaptable frameworks. The MGL stands out by offering a fully transparent, user-friendly ecosystem where tools and workflows can be easily integrated and customised. As a customisable approach, it can also include functionalities from KBase, MG-RAST, BV-BRC, or MGnify.

The MGL provides an ideal foundation for addressing critical challenges in health and disease by integrating complex, multi-step bioinformatic processes into accessible, end-to-end pipelines. It hosts complete workflows, such as AMR gene identification, guiding analysis from raw patient sequence data to final results without requiring command-line expertise. By including steps like quality control, genome assembly, and plasmid screening in a user-friendly interface, the MGL enables clinicians and public health scientists to perform vital analyses for diagnostics and research without specialised bioinformatics expertise.

The platform supports a range of One Health-focused applications, including AMR gene detection in diverse samples, plasmid transfer tracking, pathogen detection, virus characterisation, epidemiological surveillance, and dysbiosis assessment in clinically relevant environments. Its workflows are interoperable, reusable, and implementable across different laboratories, addressing the interoperability challenges posed by heterogeneous, non-standardised software and data systems between infrastructures^76^. The MGL promotes standardised and robust workflow analysis, generating data shareable between infrastructures, providing a centralised environment for One Health investigations. By integrating taxonomic profiling, pathogen detection, AMR screening, and MGE characterisation into structured workflows, it streamlines the transition from raw multi-sectoral data to comprehensive One Health insights.

Ecosystem and biodiversity research, particularly in monitoring microbial communities in soils, oceans, and other ecosystems, can benefit from integrating microbiological and ecological data analyses. The joined forces between the Galaxy-Ecology and microGalaxy communities provide a guidance framework for best practices in microbial ecological data analysis^77^, promoting interdisciplinary research. These efforts are particularly valuable for long-term environmental monitoring projects, enabling researchers to track microbial shifts in diverse ecosystems such as aeromicrobiomes, root-associated plant environments, and fungal populations like *Trichoderma* species in soil^53^. By enabling microbiological and environmental data integration, the platform enables researchers to apply standardised and reproducible approaches to linking microbial insights with ecological ones.

A central question for integrated analysis platforms is not whether comparable tools or workflows outperform each other, but what becomes feasible, reproducible, and accessible within a shared infrastructure. The tools integrated into the MGL are generally independently benchmarked and widely used in microbiology research. Rather than re-benchmarking or replacing optimised local implementations, MGL focuses on accessibility, interoperability, and reproducibility. By lowering technical barriers, providing curated workflows with transparent provenance, and integrating heterogeneous tools and data, MGL enables analyses that would otherwise require substantial local setup, maintenance, and expertise. Choosing MGL therefore represents a trade-off: prioritising practical, reproducible, and collaborative workflows over maximal performance tuning, particularly where rapid onboarding, method standardisation, and transparent reuse are critical.

Despite its strengths, the MGL faces technical, conceptual, and operational limitations. It inherits constraints from host Galaxy infrastructures, including finite compute, storage, and concurrency on public instances, which can limit large-scale or highly parallel workloads. However, Galaxy’s ecosystem and the possibility of extending its external computational and storage resources or deploying the MGL on institutional infrastructure provide clear pathways for scaling beyond public resources. Data privacy represents a related constraint; sensitive human datasets are generally unsuitable for analysis on public servers. Whereas Galaxy can support secure analyses in local or controlled environments, such deployments, however, require additional administrative expertise and operational overhead compared to the “click-to-use” experience of online instances. MGL’s tool diversity is both a major strength and a usability and maintenance challenge. A large and rapidly expanding microbiology data analysis landscape can be overwhelming for users and difficult to sustain over time. While MGL mitigates this through structured curation and semantic organisation (e.g., EDAM-based annotation), the long tail of tools remains inherently dynamic. Similarly, MGL workflows span different types and maturity or quality levels, which support diverse user needs but can introduce variability in reproducibility guarantees across workflow tiers. This is partly addressed through explicit best-practice requirements and stronger validation for higher-level workflows. Because MGL is community-driven, its long-term sustainability depends on continued community engagement, a global challenge for open research infrastructures, and continued investment in maintenance. In addition, keeping reference datasets and databases current and well governed is operationally demanding. Unlike many comparable resources that rely on dedicated data managers or centrally controlled reference repositories, the MGL leverages community processes and automation where possible, but reference data stewardship remains a recurring effort. Finally, several frequently cited concerns about Galaxy, such as scalability, security suitability, or software quality, have been argued to often stem from persistent misconceptions rather than intrinsic platform limitations, and are increasingly addressed through ongoing technical and governance developments^78^.

Looking ahead, a key direction for microbial research is extending omics integration, for example, with (meta)pangenomics or (meta)genome-scale metabolic modelling. Supporting such analyses within the MGL would enable system-level insights by inferring microbial genes, functions, interactions, and metabolic fluxes in complex ecosystems. This aligns with the microGalaxy roadmap’s core principles (Supplementary Note 2): consolidating state-of-the-art tools, adapting to scientific advances, prioritising reference data management, fostering community-driven innovation, strengthening multi-omics integration and developing tailored workflows for emerging research areas, including host-microbe interactions and environmental microbiome monitoring. Such integrations can also support holo-omic approaches, enabling a more comprehensive understanding of host-microbiome interactions, and enhancing our ability to study ecosystem services including carbon cycling, pollutant degradation, and climate resilience, and microbial responses to global changes. To further enhance collaborative and in-depth microbial community analysis, MGL will integrate resources like Anvi’o^79^ and Nextstrain^80^, expanding microbial data analysis beyond bacteria to include viruses, archaea, and eukaryotes, e.g., by building on existing efforts SARS-CoV-2^81^ and Mpox. Another priority is federated data analysis, through collaborations with EMBL-EBI and the MGnify team to integrate all MGnify pipelines into MGL and enable data submission to MGnify from the MGL. This will scale computational capabilities beyond individual facilities and across public Galaxy servers, addressing the roadmap’s goal to improve scalability and computational efficiency. As microbiological data generation accelerates, efficient resource allocation becomes increasingly important. The MGL relies on dynamic computational resource allocation based on the Galaxy’s Total Respective Vortex (https://github.com/galaxyproject/total-perspective-vortex). To ensure that users can run large-scale analyses without overloading resources, this will be further improved through the development of predictive tools allowing for estimating resource requirements from input dataset properties such as k-mers for assembly and binning. All these efforts will be supported by (i) reference data management initiatives to automate reference data integration, curate “reference data packs” for common use cases (e.g., AMR genes, 16S rRNA, viral genomes), and integrate datasets from external resources like AllTheBacteria^82^, Logan^83^, or MGnify; and (ii) enhanced training and community engagement, including annual hackathons, partnerships with ELIXIR and Australian BioCommons, and continued contributions to the Galaxy Training Academy, ensuring the platform remains accessible and responsive to the evolving needs of the global microbial research community.

By providing a comprehensive suite of workflows, tools, and training resources representing the almost-full spectrum of diverse and complex microbiome analyses, the MGL marks a fundamental shift from static repositories to a dynamic, community-driven ecosystem. It connects powerful technologies within an accessible and collaborative framework, removing technical barriers and encouraging researchers to pursue questions previously beyond their reach. As a central platform for discovery, MGL is set to not only accelerate research pace but also to fundamentally deepen our collective understanding of the microbial world. Thus, the MGL is emerging as a microbiology cornerstone by serving hundreds of thousands of users and by setting a standard for collaborative research platforms across all scientific disciplines.

## Materials and methods

### Survey and use cases

To assess the needs, preferences, and challenges of the microbiology research community, a survey was conducted between March and September 2023. The survey aimed to gather insights into how researchers interact with the Galaxy platform for microbiology research and identify areas for improvement. The survey was developed by the microGalaxy community and was distributed via a number of mechanisms such as the Galaxy Project website, mailing lists (e.g., ELIXIR, Australian BioCommons), microbioinformatics Slack space, and social media channels. It consisted of 16 questions (Supplementary Method 1) that covered several categories to understand end users, including (i) research focus and community demographics, (ii) tools and workflows, (iii) training and support, and (iv) future developments. The survey included multiple-choice, Likert scale, and open-ended questions to allow the collection of both quantitative and qualitative information^84^.

Participation was voluntary, and participants were invited to respond after an introduction to the survey goals. A total of 130 respondents (Supplementary Data 2) participated in the survey, representing a diverse range of geographical locations, institutions, and research domains. The survey exhibits bias due to its distribution via Galaxy-related channels, most notably the limited participation from the Global South. It nonetheless yields valuable direct feedback that could not be reliably collected using alternative methods. Quantitative responses were analysed using descriptive statistics to summarise trends and identify prevalent themes. Qualitative responses to open-ended questions were coded and analysed thematically to extract insights into specific challenges and recommendations.

Survey participants who indicated a willingness to be contacted were sent a follow-up invitation, including a structured document (Supplementary Method 2) to collect detailed information about their use cases. This document included (i) research objectives and questions, (ii) methods employed, including experimental techniques, data generation approaches, and analysis pipelines, and (iii) tools and workflows used within and outside Galaxy. The 21 use cases were collected and anonymised (Supplementary Data 8). Selected use cases were elaborated upon in the main text of this study, and all use case respondents were invited to contribute to the manuscript preparation. Survey participation was anonymous and voluntary. Results did not contain personal data, and participants gave informed consent by voluntarily completing and submitting the survey after they were clarified of its purpose. No human research participant ethics were violated.

### Citation extraction and annotation

To analyse the impact of Galaxy on microbiology research, citations of the major Galaxy papers were extracted as described in Supplementary Fig. 5 using Python Programming Language (v3.8.19, RRID:SCR_008394) within a Jupyter Notebook (v1.0.0, RRID:SCR_018315). First, the 8 major publications of the Galaxy Project were extracted from the Galaxy Project’s Google Scholar profile (https://scholar.google.com/citations?hl=en&user=3tSiRGoAAAAJ) using the scholarly package (v 1.7.11)^85^. Second, citations of these publications were retrieved on Semantic Scholar^86^ via its Application Programming Interface (API) using the Python package requests (v2.32.3). The collected data included the publication years, titles, and abstracts. Citations of the 8 major publications of the Galaxy project were annotated as microbiology-related if their titles or abstracts contained at least one of the 25 predefined keywords relevant to microbiology research. To validate the classifier’s ability to distinguish microbiology-related from non-microbiology-related citations, a manual validation was performed (Supplementary Note 1**)**. A random subset of 5% of entries from Supplementary Data 4 (microbiology-related citations) and 5% of entries from Supplementary Data 3 (all citations), excluding titles already present in Supplementary Data 4, was selected (total of 549 publications). Each publication was manually reviewed based on its title and, when available, abstract, and classified as microbiology-related or non-microbiology-related according to the same inclusion criteria used for the automated classification. The resulting manual labels were compared with the binary automated classification to calculate true positives, false positives, true negatives, and false negatives, from which precision, recall, F1-score, and accuracy were derived (Supplementary Data 11). The validation demonstrated good agreement between automated and manual classification, achieving an F1-score of 82.5%, precision of 87.6%, recall of 77.9%, and an accuracy of 89.6%.

The microbiology-related citations were then associated with topics: “Ecosystem Dynamics & Biodiversity” and/or “Health & Disease” if titles or abstracts contain defined keywords for each topic. If neither title nor abstracts contain the respective keywords, the topic is set to unlabelled. Additionally, microbiology-related citations were further categorised into three dimensions given keywords in their titles and abstracts to enable a detailed analysis of the research themes addressed in the citing papers: (i) Targeted Organisms (e.g., Bacteria, Virus), (ii) Technical Targets (e.g., Isolate, Community (taxonomy) profiling), and (iii) Methods (e.g., Metabarcoding, (Meta)genomics, (Meta)transcriptomics). To validate the automated classification, a second independent manual review was conducted (Supplementary Note 1**)**. A subset of 1% (*n* = 30 publications) of the entries in Supplementary Data 4 that includes all microbiology-related citations of the 8 major Galaxy Project publications until the end of 2025 was randomly selected. Each publication was manually classified according to the criteria outlined in Supplementary Fig. 5, encompassing the four category dimensions with their predefined keywords: (i) Targeted Organisms, (ii) Technical Targets, (iii) Methods, and (iv) Topics (Supplementary Data 12). For each publication, the manually assigned keywords and categories were compared against the automated annotations, and precision, recall, and F1-scores were calculated for each classification dimension using micro- and macro-averaging approaches. The validation showed strong agreement for Topics (F1 = 88.6%) and Methods (F1 = 84.4%), while performance was lower for some fine-grained Technical Target categories (F1 = 33.8%). Because the validation was performed on a smaller random subset (*n* = 30), category-specific precision and recall estimates should be interpreted as approximate and primarily intended to assess overall agreement patterns across classification dimensions rather than precise performance estimates.

### Galaxy framework and the Microbiology Galaxy Lab (MGL)

The Galaxy framework^18^ is a robust and widely adopted platform enabling accessible and reproducible bioinformatics analyses. The MGL extends the Galaxy framework using a domain-specific interface called a Galaxy Lab. A Galaxy Lab is built and deployed with the Galaxy framework and Galaxy Labs Engine (https://labs.usegalaxy.org.au)^87^. It provides users with a focused workspace retaining all the benefits of the Galaxy ecosystem, including accessibility, scalability, and reproducibility. With templated elements, each server can host a similar version of a Galaxy Lab, centralising community resources while allowing adaptations such as server-specific branding and support links.

The MGL is deployed as a dedicated subdomain on several public Galaxy servers usegalaxy.eu, usegalaxy.org, usegalaxy.org.au, usegalaxy.fr. This availability provides any users (from academia or not) with streamlined access to its suite of tools and workflows for microbiological data analysis. Furthermore, it leverages the robust computational resources of these host infrastructures, thereby removing the need for local software installation or specialised hardware on the part of the end-user. For additional capacity, features like Bring Your Own Compute (BYOC) and Bring Your Own Storage (BYOS) are available, enabling seamless integration of external compute or storage resources to further scale analyses.

All resources supporting the MGL, including its customised interface and lists of tools, workflows, and training materials, are curated and stored within the Galaxy CoDex GitHub repository (https://github.com/galaxyproject/galaxy_codex), ensuring the versioning and documentation of the MGL components.

To facilitate local deployment of the MGL, a Docker container is available at quay.io/galaxy/docker-galaxy-microbiology-lab. This container is built on the latest Galaxy image (quay.io/bgruening/galaxy) and extends it by including all MGL tools and the interface. The container is maintained on a dedicated GitHub repository (https://github.com/usegalaxy-eu/docker-galaxy-microbiology-lab). Unlike the public MGL servers, the container does not include the full reference data because the complete dataset exceeds 10TB. Instead, reference data are downloaded on-demand when a tool that requires them is first executed. For users needing immediate access to all reference data, we recommend using the public MGL servers.

### Community-curated tools

Focused on microbiology research, the MGL facilitates access to tool suites, workflows, and training materials essential for microbiological data analysis. Available tools are supported by the microGalaxy community, an engaged group of practitioners experienced in microbiological data analysis. Tools are sourced from the Galaxy ToolShed^88^ (https://toolshed.g2.bx.psu.edu), a central repository hosting tools wrapped and made available for deployment on the Galaxy platform, facilitating their straightforward access and installation on the different Galaxy servers. The tool wrappers provide the integration layer between the software or tool command-line and the Galaxy platform, defining inputs/outputs (including their formats) and parameters. They are developed and maintained by the global Galaxy community and groups such as the Intergalactic Utilities Commission (**IUC**) (https://galaxyproject.org/iuc/). Their source code is stored in open-source, publicly available GitHub repositories (https://github.com/galaxyproject/planemo-monitor). Galaxy tool wrapper development, testing, and validation are conducted using Planemo^89^, a command-line Software Development Kit (**SDK**). The use of Planemo promotes the creation of consistent, high-quality tool wrappers for the Galaxy platform. Following development and community approval, these tools are deposited in the Galaxy ToolShed.

Any tool dependencies are resolved using packages or containers available through **Bioconda**^90^ or **Biocontainers**^91^. When the dependencies are updated, tools maintained by the IUC or other community tool repositories undergo a semi-automated updating process. Once reviewed and approved by a community member, the updated tool becomes publicly available on the ToolShed and is automatically updated on the major Galaxy servers where it is installed. This process ensures that users have access to the latest versions of tools. At the same time, legacy versions are retained to support reproducibility and maintain standardised workflows, enabling scientists and clinical centres to repeat analyses as tools evolve.

Tools in the Galaxy ToolShed are organised into individual entries, each representing a single command, or they are grouped into tool suites, which are sets of related software commands. In the MGL, the tool suites are carefully selected based on their relevance to microbial research, their widespread use within the community, and their adherence to Galaxy’s development best practices. The selection process is guided by input from experts within the microGalaxy community, ensuring the inclusion of robust, well-maintained tools that are essential for various analyses, from genome assembly to complex tasks like MAGs building or antimicrobial resistance analysis. Curation is managed through the Galaxy CoDex GitHub repository using a semi-automated process: weekly automatic updates add new, potentially interesting tools to a review list, and microGalaxy community members regularly evaluate this list. Approved tools are then added to the community-curated tools list, maintaining an up-to-date and authoritative collection. It is the general Galaxy policy to continue hosting tools even if they are no longer supported by their developers, deprecated, or generally not used, in order to ensure the continuous reproducibility of analyses. However, the microGalaxy community focuses on providing only state-of-the-art tools. As such, tools are regularly reviewed in microGalaxy community meetings, and outdated tools may be deprecated and removed from the MGL. These deprecated tools remain accessible through public Galaxy servers, but they are no longer displayed in the MGL interfaces. While the technical maintenance of the tool suites is handled by a semi-automated update infrastructure, the selection and curation of tools for the MGL is an active, community-driven effort supported by the microGalaxy community.

### Community-curated workflows

Workflows included in the MGL are selected based on their relevance to microbiology research, adherence to Galaxy best practices, compatibility with training material or peer-reviewed studies, and microGalaxy community adoption metrics such as usage and demand. The MGL workflows exist at four levels of development, providing varying degrees of accessibility, adherence to best practices, and maintenance: (i) Public workflows: workflows in public Galaxy servers that are shared publicly and tagged by the microGalaxy community with #microGalaxy. They require minimal effort to create and, as Galaxy workflows intrinsically store the exact tool versions used at the time of workflow construction, they support reproducible execution. They are readily accessible to the research community and offer a starting point for users seeking to conduct microbiological data analysis; (ii) FAIR Workflows: workflows published in WorkflowHub^30^, a workflow registry, and annotated with metadata such as creator information and licensing. These workflows may not include test data, but they offer documented and structured analysis workflows for microbiology research; (iii) Workflows related to GTN tutorials: workflows supporting tutorials on the Galaxy Training Network (GTN)^63,64^. These workflows adhere to Galaxy’s best practice, i.e., including clear annotations, proper input and output parameter definitions, formal documentation (such as creator information and licensing), and test data; and (iv) Peer-reviewed & FAIR workflows: subset of workflows available through WorkflowHub that has been developed via the Intergalactic Workflow Commission (IWC) (https://github.com/galaxyproject/iwc) to adhere to Galaxy’s best practices, including test data, and support state-of-the-art analyses. Stored in the IWC GitHub repository, they are supported by a semi-automated updating system. When a Galaxy tool used in the workflow is updated, a continuous integration (**CI**) pipeline automatically tests the workflow against the updated tool version. Once a microGalaxy community member reviews and approves the results, the updated workflow becomes publicly available on WorkflowHub, Dockstore^31^, and the major Galaxy instances. This process ensures that MGL workflows remain up-to-date and reproducible, making them an invaluable resource for microbial research. Galaxy users can easily import workflows from IWC website, WorkflowHub, or Dockstore directly into their Galaxy server, enabling seamless integration into their analysis pipelines.

Similar to the tools, the workflow curation process for the four distinct workflow types is handled through the Galaxy CoDex GitHub repository via a semi-automated system. This process involves weekly automatic updates that introduce new, potentially valuable workflows to a review list. microGalaxy community members regularly assess this list according to explicit technical and scientific criteria to ensure reproducibility and long-term usability. Approved workflows, i.e., the community-curated workflows, are added to the Community-curated Workflows list and listed in MGL, ensuring the collection remains current and authoritative.

### Training materials

Training is critical to equipping users with the knowledge needed to fully leverage the platform’s capabilities. The global Galaxy community has developed a comprehensive suite of over 400 tutorials, which are available on the GTN. The microGalaxy community curates tutorials by tagging microbiology-focused ones with the keyword “microGalaxy” (https://training.galaxyproject.org/training-material/search2?query=microgalaxy). These tutorials cover various topics, from 16S rDNA gene amplicon analysis (https://gxy.io/GTN:T00441) to more complex analyses like metagenomics (https://gxy.io/GTN:T00385) or metaproteomics (https://gxy.io/GTN:T00221). The GTN tutorials include step-by-step instructions and test data. They are designed to support learners of varying expertise levels, from beginners to experienced researchers. Many tutorials are also supported by captioned video recordings hosted on YouTube to support flexible learning for a broader audience.

The MGL tutorials are continually updated to incorporate state-of-the-art tools, workflows, and methodologies, ensuring users can access current information. Many tutorials are structured into learning pathways, providing a step-by-step progression of topics, guiding learners through increasingly complex analyses. These learning pathways are designed to build foundational knowledge while advancing skills for handling cutting-edge microbiological data analyses. In addition to the available tutorials, MGL supports a range of training events, such as workshops, hackathons, and online seminars focusing on current topics related to microbiology. These events allow users to learn, engage directly with bioinformatics experts, share experiences, and deepen their understanding of microbiological computational analyses.

### Resource aggregation and annotation

Resource annotation and ontologies are essential for ensuring consistency and improving discoverability across the MGL. To achieve this, the MGL employs the EDAM ontology^72^. This ontology-based approach ensures consistent descriptions across the platform, streamlining resource discovery and enabling users to quickly find resources tailored to their research needs.

All MGL resources are aggregated into the Galaxy CoDex (https://github.com/galaxyproject/galaxy_codex), a comprehensive catalogue that integrates metadata from the Galaxy ecosystem, along with the international registries for bioinformatic tools, bio.tools^92,93^ (https://bio.tools/), and workflows, WorkflowHub^30^ (https://workflowhub.org/). The CoDex enables the creation of lists and widgets that can be embedded into websites, offering seamless access to up-to-date tools, workflows, and training materials.

During a resource aggregation phase, missing tool annotations were identified, particularly for tools lacking EDAM ontology terms or links to software registries such as bio.tools. To address this, an annotation process was employed by the microGalaxy community, including dedicated hackathons^69,70^. These efforts resulted in over 315 tool suites being annotated and more than 35 tutorials being enriched with relevant EDAM terms. This process improved the categorisation and discoverability of resources within the MGL and provides a model for other scientific communities to follow in organising their resources.

### Data visualisation

To analyse and visualise the summary data presented in this study, a set of scripts in RMarkdown (https://github.com/rstudio/rmarkdown) was created. These scripts streamlined the generation of figures and statistical analyses, allowing for reproducibility and updates as new data became available. Each script ingests tables generated by Galaxy CoDex, representing the MGL resources and containing metadata and EDAM annotations for tools, training materials, and workflows. This setup enables the calculation of key metrics such as total counts, distribution across EDAM terms, and user feedback statistics.

All analyses were run on R (v4.3.1, RRID:SCR_001905) with the use of *data*.table (v1.14.8, RRID:SCR_026117) and *tidyr* (v1.1.3, RRID:SCR_017102) libraries for data manipulation, and the *stringr* (v1.5.0, RRID:SCR_022813) library for text processing. Visualisations were primarily created with *ggplot2* (v3.4.4, RRID:SCR_014601), while *ggrepel* (v0.9.3, RRID:SCR_017393), *ggtext* (v0.1.2, RRID:SCR_026470), and *ggh4x* (v0.2.5) were used for labelling, rich text formatting, and facet customisation, respectively. The library *patchwork* (v1.3.2, RRID:SCR_024826), was used to compose plots into the same graphic, and the libraries *colorspace* (v2.1-2) and *paletteer* (v2.1-0) ensured accessible colour schemes, where *packcircles* (v0.3.5) handled circle-packing layouts, and *shadowtext* (v0.1.2) improved label readability. Additionally, *extrafont* (v0.18) was used to manage custom fonts.

### Reporting summary

Further information on research design is available in the Nature Portfolio Reporting Summary linked to this article.

## Supplementary information

Transparent Peer Review file

Supplementary information

Description of Additional Supplementary files

Supplementary Data 1

Supplementary Data 2

Supplementary Data 3

Supplementary Data 4

Supplementary Data 5

Supplementary Data 6

Supplementary Data 7

Supplementary Data 8

Supplementary Data 9

Supplementary Data 10

Supplementary Data 11

Supplementary Data 12

Reporting Summary

## Data Availability

All figures, tables, and methods associated with this study are available in the dedicated GitHub repository (https://github.com/usegalaxy-eu/microbiology_galaxy_lab_paper_2026). To ensure reproducibility and long-term accessibility, a versioned release of the dataset was created on **Zenodo**^94^ on July 7th, 2026. Any updates to the repository will be tracked, but the version archived on Zenodo corresponds to the data used in this study.

## Code availability

The code used to conduct the data analyses and generate the figures presented in this paper can be found on a dedicated GitHub repository (https://github.com/usegalaxy-eu/microbiology_galaxy_lab_paper_2026) and Zenodo^94^. The Microbiology Galaxy Lab (MGL) resources, including sources, tools, workflows, and related code, are integrated into the Galaxy CoDex GitHub repository (https://github.com/galaxyproject/galaxy_codex) and Zenodo^95^.

## References

[1] Di Bella, J. M., Bao, Y., Gloor, G. B., Burton, J. P. & Reid, G. High throughput sequencing methods and analysis for microbiome research. *J. Microbiol. Methods* **95**, 401–414 (2013). 10.1016/j.mimet.2013.08.011 24029734

[2] Reuter, J. A., Spacek, D. V. & Snyder, M. P. High-throughput sequencing technologies. *Mol. Cell* **58**, 586–597 (2015). 10.1016/j.molcel.2015.05.004 PMC4494749 26000844

[3] Filardo, S., Di Pietro, M. & Sessa, R. Current progresses and challenges for microbiome research in human health: a perspective. *Front. Cell. Infect. Microbiol.* **14**, 1377012 (2024). 10.3389/fcimb.2024.1377012 PMC11024239 38638832

[4] Goodswen, S. J. et al. Machine learning and applications in microbiology. *FEMS Microbiol. Rev.* **45**, fuab015 (2021). 10.1093/femsre/fuab015 PMC8498514 33724378

[5] Berrios, D. C., Beheshti, A. & Costes, S. V. FAIRness and usability for open-access omics data systems. *AMIA* Annual Symposium Proceedings *AMIA Symposium*. **2018**, 232–241 (2018). PMC6371294 30815061

[6] Wilkinson, M. D. et al. The FAIR Guiding Principles for scientific data management and stewardship. *Sci. Data* **3**, 160018 (2016). 10.1038/sdata.2016.18 PMC4792175 26978244

[7] Ladner, J. T., Grubaugh, N. D., Pybus, O. G. & Andersen, K. G. Precision epidemiology for infectious disease control. *Nat. Med.* **25**, 206–211 (2019). 10.1038/s41591-019-0345-2 PMC7095960 30728537

[8] Köser, C. U. et al. Whole-genome sequencing for rapid susceptibility testing of *M. tuberculosis*. *N. Engl. J. Med.* **369**, 290–292 (2013). 10.1056/NEJMc1215305 PMC3836233 23863072

[9] Khan, M. M. et al. Multi-omics strategies uncover host–pathogen interactions. *ACS Infect. Dis.* **5**, 493–505 (2019). 10.1021/acsinfecdis.9b00080 PMC13488112 30857388

[10] Deng, L., Yin, H., Tan, K. S. W. & Tsaousis, A. D. Editorial: Zoonotic diseases: epidemiology, multi-omics, and host-pathogen interactions. *Front. Microbiol.* **15**, 1454659 (2024). 10.3389/fmicb.2024.1454659 PMC11488215 39430108

[11] Romanis, C. S., Timms, V. J., Nebauer, D. J., Crosbie, N. D. & Neilan, B. A. Microbiome analysis reveals *Microcystis* blooms endogenously seeded from benthos within wastewater maturation ponds. *Appl. Environ. Microbiol.* **90**, e0158523 (2024). 10.1128/aem.01585-23 PMC10807444 38117057

[12] Shaffer, J. P. et al. Standardized multi-omics of Earth’s microbiomes reveals microbial and metabolite diversity. *Nat. Microbiol.* **7**, 2128–2150 (2022). 10.1038/s41564-022-01266-x PMC9712116 36443458

[13] Keneally, C. et al. Multi-omics reveal microbial succession and metabolomic adaptations to flood in a hypersaline coastal lagoon. *Water Res.* **280**, 123511 (2025). 10.1016/j.watres.2025.123511 40147302

[14] Prieto Riquelme, M. V. et al. Demonstrating a comprehensive wastewater-based surveillance approach that differentiates globally sourced resistomes. *Environ. Sci. Technol.* **56**, 14982–14993 (2022). 10.1021/acs.est.1c08673 PMC9631994 35759608

[15] Spänig, S. et al. A multi-omics study on quantifying antimicrobial resistance in European freshwater lakes. *Environ. Int.* **157**, 106821 (2021). 10.1016/j.envint.2021.106821 PMC8364379 34403881

[16] One Health High-Level Expert Panel (OHHLEP) et al. One Health: A new definition for a sustainable and healthy future. *PLOS Pathog*. **18**, e1010537 (2022). 10.1371/journal.ppat.1010537 PMC9223325 35737670

[17] Straub, D. et al. Interpretations of environmental microbial community studies are biased by the selected 16S rRNA (Gene) amplicon sequencing pipeline. *Front. Microbiol.* **11**, 550420 (2020). 10.3389/fmicb.2020.550420 PMC7645116 33193131

[18] The Galaxy Community The Galaxy platform for accessible, reproducible, and collaborative data analyses: 2024 update. *Nucleic Acids Res.* **52**, W83–W94 (2024). 10.1093/nar/gkae410 PMC11223835 38769056

[19] Nassar, L. R. et al. The UCSC Genome Browser database: 2023 update. *Nucleic Acids Res.* **51**, D1188–D1195 (2023). 10.1093/nar/gkac1072 PMC9825520 36420891

[20] Sayers, E. W. et al. Database resources of the National Center for Biotechnology Information. *Nucleic Acids Res.* **50**, D20–D26 (2022). 10.1093/nar/gkab1112 PMC8728269 34850941

[21] Yuan, D. et al. The European Nucleotide Archive in 2023. *Nucleic Acids Res.* **52**, D92–D97 (2024). 10.1093/nar/gkad1067 PMC10767888 37956313

[22] Richardson, L. et al. MGnify: the microbiome sequence data analysis resource in 2023. *Nucleic Acids Res.* **51**, D753–D759 (2023). 10.1093/nar/gkac1080 PMC9825492 36477304

[23] Schaaf, S., Erxleben-Eggenhofer, A. & Gruening, B. Galaxy and RDM: being more than a workflow manager: living the data life cycle. *Proc. Conf. Res. Data Infrastruct*. **1**, (2023).

[24] Bolyen, E. et al. Reproducible, interactive, scalable and extensible microbiome data science using QIIME 2. *Nat. Biotechnol.* **37**, 852–857 (2019). 10.1038/s41587-019-0209-9 PMC7015180 31341288

[25] Blomer, J., Buncic, P. & Fuhrmann, T. CernVM-FS: delivering scientific software to globally distributed computing resources. in *Proceedings of the First International Workshop on Network-aware Data Management* 49–56 (Association for Computing Machinery, New York, NY, USA, 2011). 10.1145/2110217.2110225.

[26] Breitwieser, F. P. & Salzberg, S. L. Pavian: interactive analysis of metagenomics data for microbiome studies and pathogen identification. *Bioinformatics* **36**, 1303–1304 (2020). 10.1093/bioinformatics/btz715 PMC8215911 31553437

[27] Bik, H. M. & Inc, P. I. Phinch: An interactive, exploratory data visualization framework for –Omic datasets. Preprint at: 10.1101/009944 (2014).

[28] McMurdie, P. J. & Holmes, S. Shiny-phyloseq: Web application for interactive microbiome analysis with provenance tracking. *Bioinformatics* **31**, 282–283 (2015). 10.1093/bioinformatics/btu616 PMC4287943 25262154

[29] Lewis, S. et al. Apollo: a sequence annotation editor. *Genome Biol.* **3**, research0082.1 (2002). 10.1186/gb-2002-3-12-research0082 PMC151184 12537571

[30] Gustafsson, O. J. R. et al. WorkflowHub: a registry for computational workflows. *Sci. Data* **12**, 837 (2025). 10.1038/s41597-025-04786-3 PMC12095652 40399296

[31] Yuen, D. et al. The Dockstore: enhancing a community platform for sharing reproducible and accessible computational protocols. *Nucleic Acids Res* **49**, W624–W632 (2021). 10.1093/nar/gkab346 PMC8218198 33978761

[32] Zoabi, R. MGnify amplicon pipeline v5.0 on Galaxy. *Galaxy Hub* https://galaxyproject.org/news/2024-11-15-mgnify-v5/ (2025).

[33] Walsh, T. R., Gales, A. C., Laxminarayan, R. & Dodd, P. C. Antimicrobial resistance: addressing a global threat to humanity. *PLOS Med.* **20**, e1004264 (2023). 10.1371/journal.pmed.1004264 PMC10317217 37399216

[34] Verstraete, W. The technological side of the microbiome. *Npj Biofilms Microbiomes* **1**, 15001 (2015). 10.1038/npjbiofilms.2015.1 PMC5515207 28721225

[35] Strepis, N. et al. BenchAMRking: a Galaxy-based platform for illustrating the major issues associated with current antimicrobial resistance (AMR) gene prediction workflows. *BMC Genomics* **26**, 27 (2025). 10.1186/s12864-024-11158-5 PMC11724594 39794695

[36] Sherry, N. L. et al. An ISO-certified genomics workflow for identification and surveillance of antimicrobial resistance. *Nat. Commun.* **14**, 60 (2023). 10.1038/s41467-022-35713-4 PMC9813266 36599823

[37] Sharma, T. et al. Galaxy ASIST: A web-based platform for mapping and assessment of global standards of antimicrobial susceptibility: A case study in Acinetobacter baumannii genomes. *Front. Microbiol.* **13**, 1041847 (2023). 10.3389/fmicb.2022.1041847 PMC9933921 36817105

[38] Merdan, O. et al. Investigation of the defective growth pattern and multidrug resistance in a clinical isolate of Candida glabrata using whole-genome sequencing and computational biology applications. *Microbiol. Spectr.* **10**, e00776–22 (2022). 10.1128/spectrum.00776-22 PMC9430859 35867406

[39] Arcari, G. et al. Genotypic Evolution of *Klebsiella pneumoniae* Sequence Type 512 during Ceftazidime/Avibactam, Meropenem/Vaborbactam, and Cefiderocol Treatment, Italy. *Emerg. Infect. Dis*. **29**, 2266–2274 (2023). 10.3201/eid2911.230921 PMC10617348 37877547

[40] Nasr, E., Henger, A., Grüning, B., Zierep, P. & Batut, B. PathoGFAIR: a collection of FAIR and adaptable (meta)genomics workflows for (foodborne) pathogens detection and tracking. *GigaScience* **14**, giaf017 (2025). 10.1093/gigascience/giaf017 PMC12466118 41004266

[41] Bihani, S. et al. Metaproteomic analysis of nasopharyngeal swab samples to identify microbial peptides in COVID-19 patients. *J. Proteome Res.* **22**, 2608–2619 (2023). 10.1021/acs.jproteome.3c00040 37450889

[42] Kruk, M. E. et al. An integrated metaproteomics workflow for studying host-microbe dynamics in bronchoalveolar lavage samples applied to cystic fibrosis disease. *mSystems* **9**, e00929-23 (2024). 10.1128/msystems.00929-23 PMC11264604 38934598

[43] Do, K. et al. A novel clinical metaproteomics workflow enables bioinformatic analysis of host-microbe dynamics in disease. *mSphere* **9**, e00793-23 (2024). 10.1128/msphere.00793-23 PMC11332332 38780289

[44] Wood, D. E. & Salzberg, S. L. Kraken: ultrafast metagenomic sequence classification using exact alignments. *Genome Biol.* **15**, R46 (2014). 10.1186/gb-2014-15-3-r46 PMC4053813 24580807

[45] Yousif, M. et al. SARS-CoV-2 genomic surveillance in wastewater as a model for monitoring evolution of endemic viruses. *Nat. Commun.* **14**, 6325 (2023). 10.1038/s41467-023-41369-5 PMC10564906 37816740

[46] Schiml, V. C. et al. Integrative meta-omics in Galaxy and beyond. *Environ. Microbiome* **18**, 56 (2023). 10.1186/s40793-023-00514-9 PMC10329324 37420292

[47] Cumbo, F., Truglia, S., Weitschek, E. & Blankenberg, D. Feature selection with vector-symbolic architectures: a case study on microbial profiles of shotgun metagenomic samples of colorectal cancer. *Brief. Bioinform.* **26**, bbaf177 (2025). 10.1093/bib/bbaf177 PMC12018301 40269516

[48] Cumbo, F. & Blankenberg, D. Characterization of microbial dark matter at scale with MetaSBT and taxonomy-aware Sequence Bloom Trees. Preprint at: 10.1101/2025.08.25.672238 (2025). 42595818

[49] Rousk, J. & Bengtson, P. Microbial regulation of global biogeochemical cycles. *Front. Microbiol.* **5**, 103 (2014). 10.3389/fmicb.2014.00103 PMC3954078 24672519

[50] Gougoulias, C., Clark, J. M. & Shaw, L. J. The role of soil microbes in the global carbon cycle: tracking the below-ground microbial processing of plant-derived carbon for manipulating carbon dynamics in agricultural systems. *J. Sci. Food Agric.* **94**, 2362–2371 (2014). 10.1002/jsfa.6577 PMC4283042 24425529

[51] Bharti, R. & Grimm, D. G. Current challenges and best-practice protocols for microbiome analysis. *Brief. Bioinform.* **22**, 178–193 (2021). 10.1093/bib/bbz155 PMC7820839 31848574

[52] Hu, B. et al. Challenges in bioinformatics workflows for processing microbiome Omics data at scale. *Front. Bioinforma.* **1**, 826370 (2022). 10.3389/fbinf.2021.826370 PMC9580927 36303775

[53] Rosolen, R. R. et al. Whole-genome sequencing and comparative genomic analysis of potential biotechnological strains of Trichoderma harzianum, Trichoderma atroviride, and Trichoderma reesei. *Mol. Genet. Genomics* **298**, 735–754 (2023). 10.1007/s00438-023-02013-5 37017807

[54] Kumaishi, K. et al. High throughput method of 16S rRNA gene sequencing library preparation for plant root microbial community profiling. *Sci. Rep*. **12**, 2045–2322 (2022). 10.1038/s41598-022-23943-x PMC9652414 36369356

[55] The Genomic Data Science Community Network Diversifying the genomic data science research community. *Genome Res.* **32**, 1231–1241 (2022). 10.1101/gr.276496.121 PMC9341509 35858750

[56] The BioDIGS Consortium, et al. Unearthing soil biodiversity through collaborative genomic research and education. *Nat. Genet*. **58**, 3–8 (2026). 10.1038/s41588-025-02442-5 PMC12799090 41461908

[57] Prayogo, F. A. et al. Metagenomic applications in exploration and development of novel enzymes from nature: a review. *J. Genet. Eng. Biotechnol.* **18**, 39 (2020). 10.1186/s43141-020-00043-9 PMC7403272 32749574

[58] Péguilhan, R. et al. Clouds influence the functioning of airborne microorganisms. *Biogeosciences* **22**, 1257–1275 (2025).

[59] Saito, M. A. et al. Results from a multi-laboratory ocean metaproteomic intercomparison: effects of LC-MS acquisition and data analysis procedures. *Biogeosciences* **21**, 4889–4908 (2024).

[60] Peters, K. et al. PhenoMeNal: processing and analysis of metabolomics data in the cloud. *GigaScience* **8**, giy149 (2019). 10.1093/gigascience/giy149 PMC6377398 30535405

[61] Giacomoni, F. et al. Workflow4Metabolomics: a collaborative research infrastructure for computational metabolomics. *Bioinforma. Oxf. Engl.* **31**, 1493–1495 (2015). 10.1093/bioinformatics/btu813 PMC4410648 25527831

[62] Davidson, R. L., Weber, R. J. M., Liu, H., Sharma-Oates, A. & Viant, M. R. Galaxy-M: a Galaxy workflow for processing and analyzing direct infusion and liquid chromatography mass spectrometry-based metabolomics data. *GigaScience* **5**, 10 (2016). 10.1186/s13742-016-0115-8 PMC4765054 26913198

[63] Batut, B. et al. Community-driven data analysis training for biology. *Cell Syst.* **6**, 752–758.e1 (2018). 10.1016/j.cels.2018.05.012 PMC6296361 29953864

[64] Hiltemann, S. et al. Galaxy Training: a powerful framework for teaching!. *PLOS Comput. Biol.* **19**, e1010752 (2023). 10.1371/journal.pcbi.1010752 PMC9829167 36622853

[65] Rasche, H. et al. Training infrastructure as a service. *GigaScience* **12**, giad048 (2023). 10.1093/gigascience/giad048 PMC10316688 37395629

[66] CSIR-IMTech & Galaxy-P Team. Analysis of Functions Expressed by Microbiomes. *Galaxy Hub* https://galaxyproject.org/events/2021-11-microbiomes/home/ (2021).

[67] Brites, D. et al. Mycobacterium tuberculosis complex NGS made easy. *Galaxy Training Network* https://training.galaxyproject.org/training-material/events/2024-06-10-mtb-ngs.html.

[68] Crosswell, L. C. & Thornton, J. M. ELIXIR: a distributed infrastructure for European biological data. *Trends Biotechnol.* **30**, 241–242 (2012). 10.1016/j.tibtech.2012.02.002 22417641

[69] Zierep, P. et al. *How to increase the findability, visibility, and impact of Galaxy tools* for your scientific community 10.37044/osf.io/qjbxc (2024).

[70] Batut, B. et al. How to improve the annotation of Galaxy resources? Outcomes of an online hackathon for improving the annotation of Galaxy resources for microbial data resources. Preprint at: 10.37044/osf.io/s7tru (2024).

[71] Batut, B. et al. Galaxy CoDex for finding tools, workflows, and training. in *F1000Research* **13**, 13:705 (2024).

[72] Ison, J. et al. EDAM: an ontology of bioinformatics operations, types of data and identifiers, topics and formats. *Bioinformatics* **29**, 1325–1332 (2013). 10.1093/bioinformatics/btt113 PMC3654706 23479348

[73] Meyer, F. et al. MG-RAST version 4—lessons learned from a decade of low-budget ultra-high-throughput metagenome analysis. *Brief. Bioinform.* **20**, 1151–1159 (2019). 10.1093/bib/bbx105 PMC6781595 29028869

[74] Arkin, A. P. et al. KBase: The United States Department of Energy Systems Biology Knowledgebase. *Nat. Biotechnol.* **36**, 566–569 (2018). 10.1038/nbt.4163 PMC6870991 29979655

[75] Olson, R. D. et al. Introducing the Bacterial and Viral Bioinformatics Resource Center (BV-BRC): a resource combining PATRIC, IRD and ViPR. *Nucleic Acids Res.* **51**, D678–D689 (2023). 10.1093/nar/gkac1003 PMC9825582 36350631

[76] Holt, K. E. et al. Tools and challenges in the use of routine clinical data for antimicrobial resistance surveillance. *Npj Antimicrob. Resist.* **3**, 37 (2025). 10.1038/s44259-025-00105-3 PMC12064641 40346194

[77] Royaux, C. et al. Guidance framework to apply best practices in ecological data analysis: lessons learned from building Galaxy-Ecology. *GigaScience* **14**, giae122 (2025). 10.1093/gigascience/giae122 PMC11816794 39937595

[78] Bacon, W. A. et al. Ten common misconceptions about Galaxy (and why they are wrong!). *PLOS Comput. Biol.* **22**, e1013869 (2026). 10.1371/journal.pcbi.1013869 PMC12912617 41701727

[79] Eren, A. M. et al. Community-led, integrated, reproducible multi-omics with anvi’o. *Nat. Microbiol.* **6**, 3–6 (2020). 10.1038/s41564-020-00834-3 PMC8116326 33349678

[80] Hadfield, J. et al. Nextstrain: real-time tracking of pathogen evolution. *Bioinformatics* **34**, 4121–4123 (2018). 10.1093/bioinformatics/bty407 PMC6247931 29790939

[81] Maier, W. et al. Ready-to-use public infrastructure for global SARS-CoV-2 monitoring. *Nat. Biotechnol.* **39**, 1178–1179 (2021). 10.1038/s41587-021-01069-1 PMC8845060 34588690

[82] Hunt, M. et al. AllTheBacteria – all bacterial genomes assembled, available, and searchable. Preprint at: 10.1101/2024.03.08.584059 (2025).

[83] Chikhi, R. et al. Logan: Planetary-Scale Genome Assembly Surveys Life’s Diversity. Preprint at: 10.1101/2024.07.30.605881 (2025).

[84] Ponto, J. Understanding and evaluating survey research. *J. Adv. Pract. Oncol.* **6**, 168–171 (2015). PMC4601897 26649250

[85] Cholewiak, S., Ipeirotis, P., Silva, V. & Kannawadi, A. scholarly. Zenodo 10.5281/ZENODO.7542349 (2023).

[86] Kinney, R. et al. *Semantic Scholar Open Data Platform.* 10.48550/ARXIV.2301.10140 (2023).

[87] Price, G. R. et al. Community-Curated Galaxy Interfaces with the Galaxy Labs Engine. Preprint at 10.20944/preprints202508.0199.v1 (2025). PMC13148399 41964931

[88] Blankenberg, D. et al. Dissemination of scientific software with Galaxy ToolShed. *Genome Biol.* **15**, 403 (2014). 10.1186/gb4161 PMC4038738 25001293

[89] Bray, S. et al. The Planemo toolkit for developing, deploying, and executing scientific data analyses in Galaxy and beyond. *Genome Res.* **33**, 261–268 (2023). 10.1101/gr.276963.122 PMC10069471 36828587

[90] Bioconda Team, T. he et al. Bioconda: sustainable and comprehensive software distribution for the life sciences. *Nat. Methods* **15**, 475–476 (2018). 10.1038/s41592-018-0046-7 PMC11070151 29967506

[91] Da Veiga Leprevost, F. et al. BioContainers: an open-source and community-driven framework for software standardization. *Bioinformatics* **33**, 2580–2582 (2017). 10.1093/bioinformatics/btx192 PMC5870671 28379341

[92] Ison, J. et al. Tools and data services registry: a community effort to document bioinformatics resources. *Nucleic Acids Res.* **44**, D38–D47 (2016). 10.1093/nar/gkv1116 PMC4702812 26538599

[93] Ison, J. et al. The bio.tools registry of software tools and data resources for the life sciences. *Genome Biol.* **20**, 164 (2019). 10.1186/s13059-019-1772-6 PMC6691543 31405382

[94] Engy Nasr, Nikos Pechlivanis, Bérénice Batut & Paulzierep. usegalaxy-eu/microbiology_galaxy_lab_paper_2026: v6.0.0. Zenodo 10.5281/ZENODO.21243955 (2026).

[95] Batut, B. et al. galaxyproject/galaxy_codex: Release 2026 June 03. Zenodo 10.5281/zenodo.20528018 (2026).

[96] Page, A. J. et al. Roary: rapid large-scale prokaryote pan genome analysis. *Bioinformatics* **31**, 3691–3693 (2015). 10.1093/bioinformatics/btv421 PMC4817141 26198102

[97] Seemann, T. Prokka: rapid prokaryotic genome annotation. *Bioinformatics* **30**, 2068–2069 (2014). 10.1093/bioinformatics/btu153 24642063

[98] Seemann, T. Seemann, T. Abricate: Mass Screening of Bacterial Genome Assemblies Against Curated Gene Sequence Databases. *Preprints*, 2026071215 2026 10.20944/preprints202607.1215.v1

[99] Bharat, A. et al. Correlation between phenotypic and in silico detection of antimicrobial resistance in Salmonella enterica in Canada using Staramr. *Microorganisms* **10**, 292 (2022). 10.3390/microorganisms10020292 PMC8875511 35208747

[100] Maki, J. J. et al. Species Delineation and Comparative Genomics within the *Campylobacter ureolyticus* Complex. *J. Clin. Microbiol.* **61**, e00046–23 (2023). 10.1128/jcm.00046-23 PMC10204631 37129508

[101] Gu, Z. Complex heatmap visualization. *iMeta* **1**, e43 (2022). 10.1002/imt2.43 PMC10989952 38868715

[102] Luo, W. & Brouwer, C. Pathview: an R/Bioconductor package for pathway-based data integration and visualization. *Bioinformatics* **29**, 1830–1831 (2013). 10.1093/bioinformatics/btt285 PMC3702256 23740750

[103] Pyles, R. B. et al. The altered TBI fecal microbiome is stable and functionally distinct. *Front. Mol. Neurosci*. **17**, 1341808 (2024). 10.3389/fnmol.2024.1341808 PMC10965628 38544523

[104] Love, M. I., Huber, W. & Anders, S. Moderated estimation of fold change and dispersion for RNA-seq data with DESeq2. *Genome Biol.* **15**, 550 (2014). 10.1186/s13059-014-0550-8 PMC4302049 25516281

[105] Tang, K. W. K., Millar, B. C. & Moore, J. E. Antimicrobial Resistance (AMR). *Br. J. Biomed. Sci.* **80**, 11387 (2023). 10.3389/bjbs.2023.11387 PMC10336207 37448857

[106] Park, C. R., Heo, H., Suh, C. H. & Shim, W. H. Uncover this tech term: application programming interface for large language models. *Korean J. Radiol.* **26**, 793 (2025). 10.3348/kjr.2025.0360 PMC12318651 40736411

[107] Gajic, I. et al. Antimicrobial susceptibility testing: a comprehensive review of currently used methods. *Antibiotics* **11**, 427 (2022). 10.3390/antibiotics11040427 PMC9024665 35453179

[108] Shahin, M., Ali Babar, M. & Zhu, L. Continuous integration, delivery and deployment: a systematic review on approaches, tools, challenges and practices. *IEEE Access* **5**, 3909–3943 (2017).

[109] Li, W. et al. The EMBL-EBI bioinformatics web and programmatic tools framework. *Nucleic Acids Res.* **43**, W580–W584 (2015). 10.1093/nar/gkv279 PMC4489272 25845596

[110] Rehm, H. L. et al. GA4GH: International policies and standards for data sharing across genomic research and healthcare. *Cell Genomics* **1**, 100029 (2021). 10.1016/j.xgen.2021.100029 PMC8774288 35072136

[111] Paszko, C. & Turner, E. *Laboratory Information Management Systems*. (CRC Press, 2018). 10.1201/9780203908419.

[112] Mirete, S. et al. Metagenome-Assembled Genomes (MAGs): advances, challenges, and ecological insights. *Microorganisms* **13**, 985 (2025). 10.3390/microorganisms13050985 PMC12114606 40431158

[113] Frost, L. S., Leplae, R., Summers, A. O. & Toussaint, A. Mobile genetic elements: the agents of open source evolution. *Nat. Rev. Microbiol.* **3**, 722–732 (2005). 10.1038/nrmicro1235 16138100

[114] ELIXIR Recommended Interoperability Resource (RIR) Selection | ELIXIR. https://elixir-europe.org/platforms/interoperability/rir-selection.

[115] Beard, N. et al. TeSS: a platform for discovering life-science training opportunities. *Bioinformatics* **36**, 3290–3291 (2020). 10.1093/bioinformatics/btaa047 PMC7214044 32044952

[116] Kanitz, A., McLoughlin, M. H., Beckman, L., Malladi, V. S. & Ellrott, K. The GA4GH task execution application programming interface: enabling easy multicloud task execution. *Comput. Sci. Eng.* **26**, 30–39 (2024). 10.1109/mcse.2024.3414994 PMC12336797 40792191

[117] Ng, P. C. & Kirkness, E. F. Whole Genome Sequencing. in *Genetic Variation* (eds Barnes, M. R. & Breen, G.) vol. 628 215–226 (Humana Press, Totowa, NJ, 2010). 10.1007/978-1-60327-367-1_12 20238084

[118] European Organization For Nuclear Research & OpenAIRE. Zenodo: Research. Shared. Preprint at 10.25495/7GXK-RD71 (2013).

[119] Ellis, M. M., Ivan, J. S., Tucker, J. M. & Schwartz, M. K. rSPACE: Spatially based power analysis for conservation and ecology. *Methods Ecol. Evol.* **6**, 621–625 (2015).

[120] InvenioRDM. https://inveniordm.web.cern.ch/.

