# Supplementary material for: Microbiology Galaxy Lab: a community-driven gateway for reproducible and FAIR analysis of microbiological data
Source: Commun Biol. 2026 Oct 29;9:1425. doi: 10.1038/s42003-026-10750-7 (PMC13720100; doi:10.1038/s42003-026-10750-7)
Supplement: Supplementary file 2 [file 42003_2026_10750_MOESM1_ESM.pdf]

nature portfolio

Peer Review File

Microbiology Galaxy Lab: a community-driven gateway for reproducible and FAIR analysis of microbiological data

Corresponding Author: Dr Bérénice Batut

This file contains all reviewer reports in order by version, followed by all author rebuttals in order by version.

Version 0:

Reviewer comments:

Reviewer #1

(Remarks to the Author)
This work presents a Galaxy Lab, which is an extension to existing Galaxy servers with a selection of tools and workflows and a dedicated landing page that is meant to specifically facilitate microbiology research.

This represents a useful addition to the field. Galaxy is an open source, web-based platform for data intensive biomedical research. Galaxy has enable thousands of analysis through the years and has brought (bio)informatic approaches and computational methods to the users. Through time it has incorporated a large battery of methods, workflows and approaches and has develop extensive collections. This Lab represents a dedicated tool set for microbiology that (appears to be) linked to the ELIXIR microbial community and as such incoporates additional information about the tools related to community acceptance. It also facilitates support and help and overall will lead to data management and analysis practices in microbiology that more closely follow the FAIR guidelines.
A number of aspects can be improved in the manuscript.

1. Description of the community. In the manuscript there are mentions to a community (it is even in the title) and there are mentions to the Microbial community, the microbial research community, the microGalaxy community... The work will improve in more clarity would be provided in this point: how do the authors define the community, what are the membership criteria and how do they envision the future development of such community.
2. Long term sustainability: the authors provide compelling examples of support by other communities/initiatives such as Galaxy or the ELIXIR Biohackathon. Still is not clear what are the longer term plans. For example are the included tools going to be revisited every few years? what are the plans to ensure tools in the lab remain state of the art? Galaxy has to host tools that have been deprecated and no longer in use so that analysis remain reproducible, how is this going to be combined with recommending the optimal/state of the art tools for new analysis in the microGalaxy Lab?
3. Selection of tools and survey. The information related to the survey and the respondents of the survey appears to be missing. This information seems to be in some kind of supplementary table, files that I am not able to access, as I am only getting a pdf file that contains the legend to the supplementary materials but not the tables themselves.
In any case the number of respondents that can be deduced from Figure 2 B seems to be not higher than 150... the authors should reflect on the relevance of the information gathered.

4. This work has a large amount of information available as supplementary material that is not directly accessible to this reviewer. The manuscript contains a link to a github with some code to reproduce the figures, but still I would expect the supplementary tables and extended tables to be directly accessible without needed to run any code. Please re-organize all that information in a way that is directly accessible.

5. Concepts such as ELIXIR and EDAM should be explained for the general public.

6. Table 1 explains the support for FAIR analysis within the Mirobiology Galaxy lab. Please make clear which of these are directly due to the use of Galaxy and which ones result from this particular extension.

Finally some of the characteristics described could be different for academic or commercial users. I suggest adding some information on what would be the best approach for a commercial user and add considerations of the free compute and storage they could access.

Reviewer #2

(Remarks to the Author)
Peer review report: COMMSBIO-25-11091-T
Title: Microbiology Galaxy Lab: The first community-driven gateway for reproducible and FAIR analysis of microbiological data
1. General Comments:

1.1 The manuscript COMMSBIO-25-11091-T, title “Microbiology Galaxy Lab: The first community-driven gateway for reproducible and FAIR analysis of microbiological data”, presents Microbiology Galaxy Lab (MGL) (https://microbiology.usegalaxy.org), a microbiology-focused Galaxy Lab that provides a curated gateway to tools, workflows, and training materials for microbiological data analysis. Built on top of the Galaxy framework, MGL leverages existing public Galaxy infrastructures (e.g., the UseGalaxy network) while offering a dedicated landing page, domain-specific tool organization, and community-maintained content.
The stated objective is to lower practical barriers to the use of complex microbiological bioinformatics methods and reproducible analyses with standardized workflows. A central claim is that MGL is community-driven and explicitly aligned with FAIR principles.
The reference list is strongly up to date, which helps position the work within the current state of the art.

1.2 Overall, I consider Microbiology Galaxy Lab a useful and timely resource. The manuscript provides multiple usage and adoption statistics that support the platform’s relevance, and it incorporates several features that increase its practical value for the field (e.g., curated tool workflows and training resources). The work is generally well structured and the methodological description is sound. In particular, the authors make a convincing case that this gateway can lower technical and infrastructure barriers, which is likely to be of interest to a broad microbiology audience, especially “classic” microbiologists and applied researchers who are not specialized in bioinformatics.

1.3 That said, several sections would benefit from refinement to strengthen clarity and the positioning of novelty. The manuscript currently provides limited discussion of the platform’s limitations (technical, conceptual, and operational) and offers only minimal justification of some methodological and design choices. In addition, the authors do not sufficiently contextualize the contribution relative to existing, widely used microbiology-oriented analysis platforms and portals. A more explicit comparison against resources such as MG-RAST, nephele https://nephele.niaid.nih.gov/ , PATRIC / BV-BRC (https://www.bv-brc.org/) or https://czid.org/ would help readers understand what is genuinely new here and under which use cases MGL offers clear advantages.

1.4 In both the graphical abstract and the main abstract, the authors emphasize the One Health paradigm and suggest that Microbiology Galaxy Lab is particularly relevant for One Health applications. However, this concept is not revisited in the body of the manuscript and is not meaningfully integrated into the results or discussion, which makes the One Health framing feel disconnected from the core contribution. I recommend that the authors either integrate One Health more substantively by adding a concrete example or short use case, or alternatively tone down/remove the One Health claim from the abstract/graphical abstract if it is not supported by the presented results.

2. Specific comments:
2.1. There is an inconsistency in the naming and cross-referencing of the supplementary materials between the main manuscript (file name: 44197_0_art_file_11743637_t51x3k) and the supplementary file (file name: 44197_0_supp_11743639_t57jzs). For instance, the manuscript refers to Extended Data Table 1, whereas the supplementary file refers to Supplementary Document 2.

2.2. In Table 1, several items are presented as “integrations” or capabilities (e.g., Jupyter and Docker) in the context of FAIR and reproducibility, but it is unclear whether these are features already implemented and available to users within MGL, or rather potential/possible options in the broader Galaxy ecosystem. The authors should provide concrete evidence for each claimed integration (e.g., a working example, documentation link, or a brief description of how it is enabled in MGL), and revise the table wording to clearly distinguish current, validated functionality from planned or general ecosystem capabilities, particularly for FAIR containerization claims.

2.3. Figure 2A shows that the majority of publications citing Galaxy are classified as not microbiology-related, but this dominant category visually overwhelms the plot and makes it difficult to compare the microbiology-relevant categories that are central to the manuscript. I suggest removing (or visually de-emphasizing) the “not microbiology-related” category in the figure (e.g., by excluding it from the displayed bars or moving it to an inset), while still reporting its percentage explicitly in the main text.

2.4. In Figure 4, the manuscript presents a taxonomy of microbiological analysis tasks and expected outputs, but the mapping to implementation is not consistently traceable. The figure indexes tool suites (via numerical labels) rather than individual tools, while the text reports overall counts for different entities (tools, tool suites, and curated workflows). This terminology and level-of-abstraction mismatch makes it difficult for readers to validate coverage. In addition, some items shown in the figure (e.g., antimicrobial peptides) appear without an associated tool suite/workflow reference in the figure.
I recommend the authors (i) clarify consistently throughout the manuscript the distinction between tools, tool suites, and workflows, including which quantities correspond to each; and (ii) ensure that every task/output in Figure 4 is linked to at

least one concrete, citable implementation element (tool suite/workflow ID, with a pointer to the corresponding Supplementary Table entry and/or an example workflow), so that readers can verify and reproduce the claimed capabilities.

2.5. In Figure 5, the manuscript states that the panels are reproduced from other publications. While these examples are informative, relying primarily on previously published figures limits the manuscript's originality and does not demonstrate how such visualizations are generated within MGL. Given that the authors include a Methods section on data visualization, I recommend replacing or complementing these panels with new, author-generated figures produced directly through MGL, ideally showing how the visualizations integrate into an end-to-end analysis workflow.

2.6. The overall quality and clarity of the figures could be improved. For example, in the graphical abstract the connecting lines between elements do not appear to follow a clear logic, and some conceptual items at the bottom (e.g., Citizen science and Education) look visually disconnected from the rest of the diagram, which makes the intended message harder to interpret. I recommend revising the layout and visual hierarchy.

Reviewer #3

(Remarks to the Author)
This manuscript presents Microbiology Galaxy Lab (MGL) as a community-driven, open-access gateway built on the Galaxy framework that enables reproducible, FAIR microbiological data analysis across a wide range of omics modalities. The authors claim that MGL (i) substantially lowers technical barriers to microbiological bioinformatics, (ii) supports reproducibility through workflow curation, provenance tracking, and FAIR-aligned practices, and (iii) has already achieved broad adoption and impact, as evidenced by usage metrics, surveys, and citation mining. Overall, the manuscript describes a large, mature, and well-integrated ecosystem rather than a prototype, and will be of interest to microbiologists, bioinformaticians, and infrastructure developers.
First, I want to thank the authors for putting together such a valuable tool for microbiologists. While many researchers have the opportunity to acquire in-depth training in meta(genomics) analysis, there is clearly a broad community that would benefit from an accessible platform such as MGL.
One strength is the clear need for this type of portal, which makes it a timely contribution. The MGL is free, globally accessible, and community-supported. The integration of more than 300 tools gives the MGL scale and breadth as well. There's also an emphasis on training, which has been quantified and is credible. Because it is based on Galaxy, the MGL inherits many desirable features, such as an emphasis on reproducibility and FAIR principles.
Some concerns
The impact of MGL is inferred from scale rather than from outcomes, e.g., the number of users, workflows, or tools. For example, a case study showing the reproducibility of workflows given software versions would be nice. Also, a concrete impact could be drawn from a comparative benchmark against local pipelines, showing how beneficial using the MGL really is. I bring this up because the real question behind platforms like this is what you were able to achieve with and without it. These additions would substantially strengthen the manuscript by demonstrating concrete analytical advantages rather than inferred impact.
The manuscript presents a citation-mining analysis to infer microbiological relevance and topic distribution. This approach is potentially prone to false positives and false negatives, and including a validation step would improve the results. For example, the authors could randomly select a subset of papers and manually annotate whether each paper is microbiology-related and on which topic.
The manuscript highlights many times the use of "curated" workflows, which is central to its reproducibility claim. However, an operational definition of "curated" would significantly strengthen the claims of reproducibility. For example, who curates workflows and under what criteria? Are the tools and databases versions pinned? How are updates, deprecations, and best-practice changes managed over time?
Finally, the Discussion would benefit from a clearer distinction between currently implemented features and planned developments, potentially in the form of a concise roadmap.
Minor comments
First paragraph of the Results = "microbiology" instead of "microbi".
Please clarify whether and how MGL can be deployed on local or institutional computing clusters.

Version 1:

Reviewer comments:

Reviewer #1

(Remarks to the Author)
My comments have been addressed.

Reviewer #3

(Remarks to the Author)
I thank the authors for the careful and constructive response to my previous comments. The revised manuscript is meaningfully strengthened, particularly the operational definition of "curated", the new validation of the citation-mining

analysis, the explicit 2026–2030 roadmap, and the documentation of local deployment via the dedicated Docker image. My major concerns have been addressed, and only a small number of points remain.

The authors have added a case study demonstrating the integration of the MGnify amplicon pipeline within the MGL (Results, "Community-curated and ready-to-use tools and workflows"), and have added a paragraph at the start of the Discussion that explicitly reframes MGL's scope as accessibility, interoperability, and reproducibility rather than method benchmarking. I find this reframing reasonable and well argued. However, the MGnify case study itself is presented qualitatively — the text states that "the Galaxy MGnify pipeline may show slight result variations due to the non-deterministic nature of tools like MAPseq" but does not quantify the agreement between the MGL re-implementation and the original. A brief, concrete comparison (e.g., correlation of OTU/ASV abundance tables, or taxonomic assignment concordance on a benchmark sample) would convert the current narrative argument for reproducibility into a measurable result, and would substantially reinforce the manuscript's central claim. I do not consider this essential for acceptance, but I recommend it.

Regarding the validation of the citation-mining classification, this concern has been addressed thoroughly. The authors performed (i) a binary document-level validation on a stratified 5% random sample (n = 549; precision 87.6%, recall 77.9%, F1 82.5%, accuracy 89.6%) and (ii) a multi-label keyword-level validation on a 1% sample (n = 30) across the four classification dimensions. The methodology, sampling seeds, and metrics are clearly described in Supplementary Method 4, with results in Supplementary Tables 11–12. Two points remain:

- The keyword-level validation reveals that the Technical Target dimension has notably weaker performance (F1 = 33.8%, recall = 23.4%) than Topics (F1 88.6%) or Methods (F1 84.4%). This is acknowledged in the supplement but is not mentioned in the main text where Technical Target distributions are presented and discussed (Figure 2 / Supplementary Figure 1). I recommend adding one sentence in the Results acknowledging that interpretations involving the Technical Target dimension should be considered with this caveat.
- The keyword-level validation set is small (n = 30), which yields wide confidence intervals on per-dimension precision and recall. Reporting 95% confidence intervals (or noting the sample-size limitation) would be appropriate.

The new validation analysis is methodologically appropriate. Sampling is stratified, seeds are fixed, and metrics are standard (TP/FP/FN/TN, precision/recall/F1, with both micro- and macro-averaging). Validation code, sampling tables, and downstream supplementary tables are deposited in the manuscript GitHub repository. Reproducibility documentation has improved substantially from the previous version, including direct links to all supplementary files in the repository README.

Open Access This Peer Review File is licensed under a Creative Commons Attribution 4.0 International License, which permits use, sharing, adaptation, distribution and reproduction in any medium or format, as long as you give appropriate credit to the original author(s) and the source, provide a link to the Creative Commons license, and indicate if changes were made.
In cases where reviewers are anonymous, credit should be given to 'Anonymous Referee' and the source.
The images or other third party material in this Peer Review File are included in the article’s Creative Commons license, unless indicated otherwise in a credit line to the material. If material is not included in the article’s Creative Commons license and your intended use is not permitted by statutory regulation or exceeds the permitted use, you will need to obtain permission directly from the copyright holder.
To view a copy of this license, visit https://creativecommons.org/licenses/by/4.0/

Bérénice Batut, PhD
Institut Français de Bioinformatique
Auvergne Bioinformatique, Université Clermont Auvergne
berenice.batut@gmail.com

April 2nd, 2026

Dear reviewers,

We sincerely appreciate your thoughtful and constructive feedback on our manuscript. Your insights have been invaluable in helping us improve the clarity and quality of our work. Below, you will find our detailed responses to each of your comments, along with the corresponding revisions we have made to the manuscript. All suggested changes have been carefully implemented and highlighted in red for your convenience.

In addition to addressing your specific comments, we have made the following improvements to the manuscript:

- **Supplementary Materials:** Extended tables and figures have been moved to the Supplementary Information section for better readability.
- **Organization:** All supplementary documents have been renamed to reflect their order of appearance in the manuscript.
- **Glossary:** A comprehensive glossary table has been added to the Introduction, listing key abbreviations and terms used throughout the manuscript, along with their definitions and relevant references or links for further information.

We believe these revisions have strengthened the manuscript and hope that it now meets your expectations. Thank you again for your time and expertise.

Sincerely,

On behalf of the authors,

Bérénice Batut, PhD
Institut Français de Bioinformatique
Auvergne Bioinformatique, Université Clermont-Auvergne
berenice.batut@gmail.com

Reviewer #1 (Remarks to the Author):

This work presents a Galaxy Lab, which is an extension to existing Galaxy servers with a selection of tools and workflows and a dedicated landing page that is meant to specifically facilitate microbiology research.

This represents a useful addition to the field. Galaxy is an open source, web-based platform for data intensive biomedical research. Galaxy has enable thousands of analysis through the years and has brought (bio)informatic approaches and computational methods to the users. Through time it has incorporated a large battery of methods, workflows and approaches and has develop extensive collections. This Lab represents a dedicated tool set for microbiology that (appears to be) linked to the ELIXIR microbial community and as such incoporates additional information about the tools related to community acceptance. It also facilitates support and help and overall will lead to data management and analysis practices in microbiology that more closely follow the FAIR guidelines.

A number of aspects can be improved in the manuscript.

1. Description of the community. In the manuscript there are mentions to a community (it is even in the title) and there are mentions to the Microbial community, the microbial research community, the microGalaxy community... The work will improve in more clarity would be provided in this point: how do the authors define the community, what are the membership criteria and how do they envision the future development of such community.

> We thank the reviewer for raising this important point and agree that the distinction between the various communities mentioned in the manuscript requires clarification. We have carefully revised the text to explicitly define and distinguish between the different communities referenced throughout the manuscript:

1. The **microGalaxy community** refers to the Special Interest Group (SIG) within the Galaxy ecosystem, which is responsible for developing and maintaining the Microbiology Galaxy Lab (MGL).
2. The **microbial research community** encompasses a broader group of researchers who do microbial data analysis (with or without Galaxy), including those who participated in the survey.
3. The **Galaxy community** refers to the wider group of users and developers engaged with the Galaxy platform across various research domains.

To clarify the definition of the microGalaxy community, we have expanded the "Robust community engagement" Section to provide more details about the microGalaxy community. Specifically, we clarify that there are no formal membership criteria for the microGalaxy community; it is open to anyone interested in contributing to or using the MGL. We have also updated the microGalaxy community webpage with this information.

Throughout the manuscript, we have ensured that the term "community" is used in a context-specific manner, clearly indicating which community is being referred to in each instance. We hope these revisions provide a clearer understanding of the roles and

relationships between the different communities involved in the development and use of the MGL.

2. Long term sustainability: the authors provide compelling examples of support by other communities/initiatives such as Galaxy or the ELIXIR Biohackathon. Still is not clear what are the longer term plans. For example are the included tools going to be revisited every few years? what are the plans to ensure tools in the lab remain state of the art? Galaxy has to host tools that have been deprecated and no longer in use so that analysis remain reproducible, how is this going to be combined with recommending the optimal/state of the art tools for new analysis in the microGalaxy Lab?

> We thank the reviewer for raising this important point regarding long-term sustainability. Technical sustainability is supported by Galaxy's semi-automated tool update infrastructure (see "Community-curated tools" and "Community-curated workflows" Sections in Materials and Methods). Tools and workflows included in the MGL are regularly reviewed during microGalaxy community meetings. While Galaxy keeps outdated tools for reproducibility, the MGL aims to provide up-to-date tools, so in this interface, such tools will be removed. We extended the "Community-curated tools" Materials and Methods Section to reflect on that policy.

3. Selection of tools and survey. The information related to the survey and the respondents of the survey appears to be missing. This information seems to be in some kind of supplementary table, files that I am not able to access, as I am only getting a pdf file that contains the legend to the supplementary materials but not the tables themselves.
In any case the number of respondents that can be deduced from Figure 2 B seems to be not higher than 150... the authors should reflect on the relevance of the information gathered.

> We apologize that the reviewer was unable to access the survey-related information. This information was included in our submission and is also available via the GitHub repository (https://github.com/usegalaxy-eu/microbiology_galaxy_lab_paper_2026) referenced in the Data Availability section. To improve accessibility, we have enhanced the visibility of the supplementary information within the GitHub repository. Additionally, we have added a discussion to the "Survey and use cases" Materials and Methods section to highlight the relevance of the survey.

4. This work has a large amount of information available as supplementary material that is not directly accessible to this reviewer. The manuscript contains a link to a github with some code to reproduce the figures, but still I would expect the supplementary tables and extended tables to be directly accessible without needed to run any code. Please re-organize all that information in a way that is directly accessible.

> We first would like to apologise for this accessibility issue. In addition to the scripts, all supplementary information and generated figures were also available in the provided GitHub repository. To make them easier to find, we have extended the manuscript GitHub repository README
(https://github.com/usegalaxy-eu/microbiology_galaxy_lab_paper_2026?tab=readme-ov-file#

supplementary-information-in-docssupplementary-folder) to include direct links to all supplementary information.

5. Concepts such as ELIXIR and EDAM should be explained for the general public.

> Thank you for this suggestion. We have now added clear, concise definitions of both ELIXIR and EDAM to the manuscript and have additionally created an expanded abbreviation and acronym table.

6. Table 1 explains the support for FAIR analysis within the Mirobiology Galaxy lab. Please make clear which of these are directly due to the use of Galaxy and which ones result from this particular extension.

> We thank the reviewer for this important clarification request. We have revised Table 1 and its accompanying legend to explicitly distinguish between functionality provided by the core Galaxy platform and functionality that is directly implemented, validated, and used within the Microbiology Galaxy Lab (MGL). Features inherited from Galaxy are now clearly marked (in italic font style) and described as such, while MGL-specific implementations and community-curated resources are explicitly identified (in non-italic font style). For each listed capability, we clarify whether it is currently available to MGL users or represents a broader Galaxy ecosystem capability.

Finally some of the characteristics described could be different for academic or commercial users. I suggest adding some information on what would be the best approach for a commercial user and add considerations of the free compute and storage they could access.

> Access to MGL is open and free for all users on public Galaxy servers such as usegalaxy.eu, usegalaxy.org, usegalaxy.org.au, and usegalaxy.fr. Registration to each of these servers is available to everyone, whether from academia, industry, or other sectors, without any restrictions. Additional resources (e.g., Bring Your Own Compute (BYOC) or Bring Your Own Storage (BYOS)) allow users to integrate their own cloud or on-premises infrastructure, ensuring scalability and dedicated resources for their projects. For local deployment and commercial use, a Docker instance of the MGL is also available.
We have added this information in the "Galaxy framework and the Microbiology Galaxy Lab (MGL)" section.

Reviewer #2

Peer review report: COMMSBIO-25-11091-T
Title: Microbiology Galaxy Lab: The first community-driven gateway for reproducible and FAIR analysis of microbiological data
1. General Comments:

1.1 The manuscript COMMSBIO-25-11091-T, title "Microbiology Galaxy Lab: The first community-driven gateway for reproducible and FAIR analysis of microbiological data", presents Microbiology Galaxy Lab (MGL) (https://microbiology.usegalaxy.org), a

microbiology-focused Galaxy Lab that provides a curated gateway to tools, workflows, and training materials for microbiological data analysis. Built on top of the Galaxy framework, MGL leverages existing public Galaxy infrastructures (e.g., the UseGalaxy network) while offering a dedicated landing page, domain-specific tool organization, and community-maintained content.
The stated objective is to lower practical barriers to the use of complex microbiological bioinformatics methods and reproducible analyses with standardized workflows. A central claim is that MGL is community-driven and explicitly aligned with FAIR principles.
The reference list is strongly up to date, which helps position the work within the current state of the art.

1.2 Overall, I consider Microbiology Galaxy Lab a useful and timely resource. The manuscript provides multiple usage and adoption statistics that support the platform's relevance, and it incorporates several features that increase its practical value for the field (e.g., curated tool workflows and training resources). The work is generally well structured and the methodological description is sound. In particular, the authors make a convincing case that this gateway can lower technical and infrastructure barriers, which is likely to be of interest to a broad microbiology audience, especially "classic" microbiologists and applied researchers who are not specialized in bioinformatics.

> We thank the reviewer for this analysis and hope indeed that a broad microbiology audience will benefit from the MGL.

1.3 That said, several sections would benefit from refinement to strengthen clarity and the positioning of novelty. The manuscript currently provides limited discussion of the platform's limitations (technical, conceptual, and operational) and offers only minimal justification of some methodological and design choices. In addition, the authors do not sufficiently contextualize the contribution relative to existing, widely used microbiology-oriented analysis platforms and portals. A more explicit comparison against resources such as MG-RAST, nephele https://nephele.niaid.nih.gov/ , PATRIC / BV-BRC (https://www.bv-brc.org/) or https://czid.org/ would help readers understand what is genuinely new here and under which use cases MGL offers clear advantages.

> We thank the reviewer for this comment. We have substantially expanded the Discussion section to explicitly address the platform's technical, conceptual, operational, and organisational limitations.

The revised text now discusses technical constraints related to shared server infrastructures and data privacy, conceptual trade-offs arising from the breadth of tools and workflow heterogeneity, and operational challenges, including community engagement, long-term maintenance, reference data stewardship, and governance processes. We further contextualise these limitations within the broader Galaxy ecosystem and outline how MGL mitigates them through structured curation, semantic organisation, automation, and community-driven governance.

We have also added a dedicated paragraph at the beginning of the Discussion section to justify the platform's methodological and design choices and clarify the rationale behind key architectural decisions.

In addition, we have expanded the manuscript to more explicitly contextualise MGL relative to existing microbiology-oriented platforms. A detailed comparison, including MG-RAST, Nephele, BV-BRC (formerly PATRIC), CZ ID (IDseq), and more platforms, is present in Supplementary Table 1. The revised Discussion Section now outlines key conceptual and architectural differences, clarifying the specific use cases in which MGL provides added value, particularly in terms of workflow transparency, version-controlled reproducibility, modular extensibility within the Galaxy ecosystem, and community-driven curation aligned with FAIR principles.

This expanded discussion provides a balanced and transparent assessment of MGL's limitations while clarifying how these challenges are actively managed within the platform.

1.4 In both the graphical abstract and the main abstract, the authors emphasize the One Health paradigm and suggest that Microbiology Galaxy Lab is particularly relevant for One Health applications. However, this concept is not revisited in the body of the manuscript and is not meaningfully integrated into the results or discussion, which makes the One Health framing feel disconnected from the core contribution. I recommend that the authors either integrate One Health more substantively by adding a concrete example or short use case, or alternatively tone down/remove the One Health claim from the abstract/graphical abstract if it is not supported by the presented results.

> Thank you for your feedback regarding the One Health framing in our manuscript. We agree that the initial emphasis in the abstract and graphical abstract was not adequately supported in the main text. To address this, we have expanded the "Health and disease" section to explicitly show how MGL's workflows and tools operationalise the One Health concept. We now highlight specific examples, such as tracking microbial community dynamics, AMR dissemination, and mobile genetic element movement across hosts and environments using tools and workflows such as Kraken, PlasmidFinder, geNomad, and PathoGFAIR. We also cite real-world applications, including wastewater-based viral surveillance and integrative meta-omic analyses. We also extended the Discussion to position MGL as a centralized platform for One Health investigations, streamlining multi-sectoral data analysis into actionable insights.

2. Specific comments:
2.1. There is an inconsistency in the naming and cross-referencing of the supplementary materials between the main manuscript (file name: 44197_0_art_file_11743637_t51x3k) and the supplementary file (file name: 44197_0_supp_11743639_t57jzs). For instance, the manuscript refers to Extended Data Table 1, whereas the supplementary file refers to Supplementary Document 2.

> Thank you for the feedback, and apologies for the lack of clarity with the supplementary information. We have revised the file names in the supplementary information, manuscript, and GitHub repository to ensure they are consistent. There might have been a technical issue during submission that caused this naming problem. In addition, we have extended the

manuscript GitHub repository README (https://github.com/usegalaxy-eu/microbiology_galaxy_lab_paper_2026?tab=readme-ov-file#supplementary-information-in-docssupplementary-folder) to include direct links to all supplementary information.

2.2. In Table 1, several items are presented as "integrations" or capabilities (e.g., Jupyter and Docker) in the context of FAIR and reproducibility, but it is unclear whether these are features already implemented and available to users within MGL, or rather potential/possible options in the broader Galaxy ecosystem. The authors should provide concrete evidence for each claimed integration (e.g., a working example, documentation link, or a brief description of how it is enabled in MGL), and revise the table wording to clearly distinguish current, validated functionality from planned or general ecosystem capabilities, particularly for FAIR containerization claims.

> We appreciate the reviewer's request for clarification. In the revised manuscript, Table 1 has been restructured to clearly differentiate between (i) functionalities inherently provided by the Galaxy framework and (ii) those specifically implemented or curated within the microGalaxy (MGL) initiative. We have also refined the wording to explicitly indicate which integrations (e.g., containerized execution via Docker and interactive analysis through Jupyter environments) are currently available within the Galaxy infrastructure underlying MGL, rather than merely conceptual possibilities. Where relevant, we now provide brief descriptions in the text explaining how these integrations are enabled and how they support FAIR and reproducible analyses.

2.3. Figure 2A shows that the majority of publications citing Galaxy are classified as not microbiology-related, but this dominant category visually overwhelms the plot and makes it difficult to compare the microbiology-relevant categories that are central to the manuscript. I suggest removing (or visually de-emphasizing) the "not microbiology-related" category in the figure (e.g., by excluding it from the displayed bars or moving it to an inset), while still reporting its percentage explicitly in the main text.

> Thank you for your feedback. Figure 2 has now been revised to visually emphasize the observed trends of the microbiology-related publications. In addition, the 2025 counts have been corrected and properly depicted in the plot.

2.4. In Figure 4, the manuscript presents a taxonomy of microbiological analysis tasks and expected outputs, but the mapping to implementation is not consistently traceable. The figure indexes tool suites (via numerical labels) rather than individual tools, while the text reports overall counts for different entities (tools, tool suites, and curated workflows). This terminology and level-of-abstraction mismatch makes it difficult for readers to validate coverage. In addition, some items shown in the figure (e.g., antimicrobial peptides) appear without an associated tool suite/workflow reference in the figure.
I recommend the authors (i) clarify consistently throughout the manuscript the distinction between tools, tool suites, and workflows, including which quantities correspond to each; and (ii) ensure that every task/output in Figure 4 is linked to at least one concrete, citable implementation element (tool suite/workflow ID, with a pointer to the corresponding Supplementary Table entry and/or an example workflow), so that readers can verify and reproduce the claimed capabilities.

> We thank the reviewer for the helpful comment to clarify the manuscript. The manuscript has been revised to ensure consistency and distinction between tools, tool suites, and workflows, each along with its corresponding counts. Regarding Figure 4, the numbers on the branches correspond to tool suites detailed in Supplementary Table 6 (https://github.com/usegalaxy-eu/microbiology_galaxy_lab_paper_2026/blob/main/docs/supplementary/supplementary_table_6.tsv). The circled letters on the figure indicate use cases listed in detail in Supplementary Table 8 (https://github.com/usegalaxy-eu/microbiology_galaxy_lab_paper_2026/blob/main/docs/supplementary/supplementary_table_8.tsv). Consequently, each task is supported by at least one, and often multiple, citable tools, frequently accompanied by published use cases demonstrating their application.
With regard to the specific example of antimicrobial peptides, it is unclear to us why the impression arose that this task is not supported, as it is explicitly numbered and described in the figure. The corresponding tool suites enabling analyses of antimicrobial peptides are listed under item 5.
To further clarify and prevent potential misunderstandings, we have revised the figure legend to explicitly state that when numbers appear before a node, all associated outputs can be generated using the corresponding tool suites.

2.5. In Figure 5, the manuscript states that the panels are reproduced from other publications. While these examples are informative, relying primarily on previously published figures limits the manuscript's originality and does not demonstrate how such visualizations are generated within MGL. Given that the authors include a Methods section on data visualization, I recommend replacing or complementing these panels with new, author-generated figures produced directly through MGL, ideally showing how the visualizations integrate into an end-to-end analysis workflow.

> We thank the reviewer for this thoughtful comment. We would like to clarify that similar visualisations as shown in Figure 5 can be generated directly within the MGL/Galaxy environment using available tools and interactive analysis environments. Panel A has been updated to a phylogenomic analysis demonstrating a core-genome phylogenetic tree generated from Prokka-annotated genomes and Roary-derived alignments, with antimicrobial resistance profiling (ABRicate, StarAMR) performed within Galaxy, and visualisation carried out in R (e.g., ggtree), fully reproducible within the Galaxy environment. Panel B corresponds to clustered heatmaps generated using Galaxy statistical tools or integrated R/Jupyter environments (e.g., ComplexHeatmap). For panel C, a similar figure can be created using pathway analysis tools available in Galaxy (e.g., KEGG/Pathview integration). Panel D represents differential abundance visualisation generated using Galaxy statistical workflows (e.g., DESeq2) and built-in heatmap modules. We have highlighted these tools and interactive environments to regenerate Figure 5 visualisations in the caption of Figure 5. Importantly, the authors were directly involved in generating some of these analyses.
The underlying workflows and tools used to perform these analyses are available within MGL. In the authors' view, these figures provide the clearest illustration of MGL usage, as they have been employed in previous publications.

2.6. The overall quality and clarity of the figures could be improved. For example, in the graphical abstract the connecting lines between elements do not appear to follow a clear logic, and some conceptual items at the bottom (e.g., Citizen science and Education) look visually disconnected from the rest of the diagram, which makes the intended message harder to interpret. I recommend revising the layout and visual hierarchy.

> We thank the reviewer for this suggestion. The graphical abstract has been revised to improve visual hierarchy and logical flow. Connecting elements were reorganised, and conceptual components were repositioned to better integrate them within the overall diagram.

Reviewer #3

This manuscript presents Microbiology Galaxy Lab (MGL) as a community-driven, open-access gateway built on the Galaxy framework that enables reproducible, FAIR microbiological data analysis across a wide range of omics modalities. The authors claim that MGL (i) substantially lowers technical barriers to microbiological bioinformatics, (ii) supports reproducibility through workflow curation, provenance tracking, and FAIR-aligned practices, and (iii) has already achieved broad adoption and impact, as evidenced by usage metrics, surveys, and citation mining. Overall, the manuscript describes a large, mature, and well-integrated ecosystem rather than a prototype, and will be of interest to microbiologists, bioinformaticians, and infrastructure developers.

First, I want to thank the authors for putting together such a valuable tool for microbiologists. While many researchers have the opportunity to acquire in-depth training in meta(genomics) analysis, there is clearly a broad community that would benefit from an accessible platform such as MGL.

One strength is the clear need for this type of portal, which makes it a timely contribution. The MGL is free, globally accessible, and community-supported. The integration of more than 300 tools gives the MGL scale and breadth as well. There's also an emphasis on training, which has been quantified and is credible. Because it is based on Galaxy, the MGL inherits many desirable features, such as an emphasis on reproducibility and FAIR principles.

Some concerns

The impact of MGL is inferred from scale rather than from outcomes, e.g., the number of users, workflows, or tools. For example, a case study showing the reproducibility of workflows given software versions would be nice. Also, a concrete impact could be drawn from a comparative benchmark against local pipelines, showing how beneficial using the MGL really is. I bring this up because the real question behind platforms like this is what you were able to achieve with and without it. These additions would substantially strengthen the manuscript by demonstrating concrete analytical advantages rather than inferred impact.

> We thank the reviewer for this thoughtful and constructive comment. We agree that demonstrating concrete outcomes is an important aspect of assessing impact. To address this, we have added a case study describing the integration of the MGnify amplicon pipeline into Galaxy, which demonstrates the reproducibility of complex workflows through explicit specification of tool and database versions. This example illustrates a concrete analytical outcome enabled by the MGL, beyond scale-based metrics.
With regard to broader research impact, we would also like to emphasize that more than 3,000 microbiology-focused publications have cited analyses supported by Galaxy. While these works are not attributable exclusively to the MGL, but rather to the broader Galaxy infrastructure and community, the tools, reference datasets, workflows, and training materials underpinning such analyses were developed significantly by the microGalaxy community and are consolidated within the MGL. This aggregation reflects a substantial research capacity that we are proud to support and further expand through continued development of the MGL (https://github.com/usegalaxy-eu/microbiology_galaxy_lab_paper_2026/blob/main/docs/supplementary/supplementary_method_2.pdf).
Regarding benchmarking against local pipelines, we note that MGL's primary role is to provide standardized, reproducible access to tools and workflows rather than to evaluate or benchmark the performance of individual methods. Such benchmarking is best performed by tool developers or method-focused studies to ensure fair and context-aware comparisons. Importantly, however, Galaxy itself provides an ideal framework for performing such benchmarks in a transparent and reproducible manner, should developers or users wish to do so. We have clarified this scope in the manuscript to better distinguish between the goals of MGL and those of method evaluation studies.

The manuscript presents a citation-mining analysis to infer microbiological relevance and topic distribution. This approach is potentially prone to false positives and false negatives, and including a validation step would improve the results. For example, the authors could randomly select a subset of papers and manually annotate whether each paper is microbiology-related and on which topic.

> We thank the reviewer for this valuable suggestion. In response, we implemented two independent manual validation procedures. First, we performed a keyword-level validation on a random subset (1%) of microbiology-related references to assess agreement across the four classification dimensions (Targeted Organisms, Technical Targets, Methods, and Topics). Second, we conducted a binary document-level validation using stratified random samples (5% each) from both microbiology-related and non-microbiology-related references to evaluate overall classification accuracy. These validation analyses, including methodology and results, are now described in the manuscript and detailed in Supplementary Methods 4.

The manuscript highlights many times the use of "curated" workflows, which is central to its reproducibility claim. However, an operational definition of "curated" would significantly strengthen the claims of reproducibility. For example, who curates workflows and under what criteria? Are the tools and databases versions pinned? How are updates, deprecations, and best-practice changes managed over time?

> We thank the reviewer for this important comment. We have revised the manuscript to provide a clearer operational definition of "curated workflows" and to explicitly describe

governance, versioning, and update policies. These clarifications are now consolidated in the Sections “Community-curated tools” and “Community-curated workflows.” The revised text specifies who performs curation, the criteria used for inclusion, how tool and workflow versions are handled, and how updates and best-practice changes are managed over time. We believe these revisions substantially improve clarity and strengthen the reproducibility claims.

Finally, the Discussion would benefit from a clearer distinction between currently implemented features and planned developments, potentially in the form of a concise roadmap.

> We thank the reviewer for their valuable feedback regarding the need for a clearer distinction between currently implemented features and planned developments in the Discussion. To address this, we have revised the Discussion section to explicitly highlight the future directions, ensuring greater clarity and precision. To support this part, we have developed a detailed roadmap outlining the strategic priorities for the microGalaxy community and the MGL from 2026 to 2030, which has been included as Supplementary Methods 2. This roadmap is also available on the microGalaxy community website (https://galaxyproject.org/community/sig/microbial) to provide the broader research community with a transparent overview of our planned developments.

Minor comments

First paragraph of the Results = “microbiology” instead of “microbi”.

> Thanks for noticing, that was a typing error and has now been corrected.

Please clarify whether and how MGL can be deployed on local or institutional computing clusters.

> Thank you for the suggestion. To facilitate local deployment or deployment on institutional computing clusters, we have created a Docker container that contains the MGL. This container is a fully functional Galaxy instance, including all tools and the MGL interface.
To keep the container size manageable, tools and reference databases are installed on demand, meaning they are only downloaded and set up when the user executes specific jobs. We have also added a section on local deployment in the manuscript.

Bérénice Batut, PhD
Institut Français de Bioinformatique
LMGE, Université Clermont Auvergne
berenice.batut@gmail.com

June 30th, 2026

Dear reviewers,

We sincerely appreciate your thoughtful and constructive feedback on our manuscript. We are grateful for the time and effort invested in evaluating our work and for the positive assessment of the revisions made in the previous round.

In response to the remaining comments, we have carefully revised the manuscript and supporting materials. Specifically, we:

- Clarified the MGnify amplicon workflow case study by referencing the previously performed comparison between the original MGnify implementation and its Galaxy integration, and revised the Results section accordingly to better reflect the demonstrated reproducibility of the workflow.
- Added a statement in the Results section acknowledging the lower performance of the Technical Targets classification dimension observed during manual validation and advising caution when interpreting these specific distributions.
- Added a statement in the Materials and methods section highlighting the limited size of the keyword-level validation sample and clarifying that category-specific performance estimates should be interpreted as approximate indicators of overall agreement patterns.
- Further improved the clarity and reproducibility of the documentation associated with the citation-mining validation analyses and supplementary materials.

All changes made in response to the reviewers' comments have been highlighted in red in the revised manuscript for ease of review.

We believe these revisions have strengthened the manuscript and hope that it now meets your expectations. Thank you again for your time and expertise.

Sincerely,

On behalf of the authors,

Bérénice Batut, PhD
Institut Français de Bioinformatique
LMGE, Université Clermont-Auvergne
berenice.batut@gmail.com

Reviewer #1 (Remarks to the Author):

My comments have been addressed.

> We thank the reviewer for the careful evaluation of the manuscript and for the constructive comments provided during the review process. We appreciate the reviewer's time and feedback, which helped improve the clarity and overall quality of the manuscript.

Reviewer #3 (Remarks to the Author):

I thank the authors for the careful and constructive response to my previous comments. The revised manuscript is meaningfully strengthened, particularly the operational definition of "curated", the new validation of the citation-mining analysis, the explicit 2026–2030 roadmap, and the documentation of local deployment via the dedicated Docker image. My major concerns have been addressed, and only a small number of points remain.

> We thank the reviewer for this positive and constructive assessment and for acknowledging the improvements made in the revised manuscript.

The authors have added a case study demonstrating the integration of the MGnify amplicon pipeline within the MGL (Results, "Community-curated and ready-to-use tools and workflows"), and have added a paragraph at the start of the Discussion that explicitly reframes MGL's scope as accessibility, interoperability, and reproducibility rather than method benchmarking. I find this reframing reasonable and well argued. However, the MGnify case study itself is presented qualitatively — the text states that "the Galaxy MGnify pipeline may show slight result variations due to the non-deterministic nature of tools like MAPseq" but does not quantify the agreement between the MGL re-implementation and the original. A brief, concrete comparison (e.g., correlation of OTU/ASV abundance tables, or taxonomic assignment concordance on a benchmark sample) would convert the current narrative argument for reproducibility into a measurable result, and would substantially reinforce the manuscript's central claim. I do not consider this essential for acceptance, but I recommend it.

> We thank the reviewer for this valuable suggestion. We agree that a quantitative comparison between the original MGnify amplicon workflow and its Galaxy implementation provides a stronger demonstration of reproducibility than a purely qualitative description.

We would like to clarify that such a comparison has already been performed as part of the migration and integration of the MGnify amplicon workflow into the Galaxy ecosystem. The results of this assessment were reported by Galaxy team members and demonstrated a high level of agreement between the original MGnify implementation and the Galaxy-based workflow, with only minor differences attributable to the non-deterministic behaviour of MAPseq. Detailed results and methodology are available in the Galaxy Project blog describing the MGnify v5 integration (https://galaxyproject.org/news/2024-11-15-mgnify-v5/).

To better reflect this existing validation and clarify the purpose of the example, we have revised the corresponding text in the Results section.

Regarding the validation of the citation-mining classification, this concern has been addressed thoroughly. The authors performed (i) a binary document-level validation on a stratified 5% random sample (n = 549; precision 87.6%, recall 77.9%, F1 82.5%, accuracy 89.6%) and (ii) a multi-label keyword-level validation on a 1% sample (n = 30) across the four classification dimensions. The methodology, sampling seeds, and metrics are clearly described in Supplementary Method 4, with results in Supplementary Tables 11–12. Two points remain:

• The keyword-level validation reveals that the Technical Target dimension has notably weaker performance (F1 = 33.8%, recall = 23.4%) than Topics (F1 88.6%) or Methods (F1 84.4%). This is acknowledged in the supplement but is not mentioned in the main text where Technical Target distributions are presented and discussed (Figure 2 / Supplementary Figure 1). I recommend adding one sentence in the Results acknowledging that interpretations involving the Technical Target dimension should be considered with this caveat.

> In response to the first point, we added a sentence in the Results section acknowledging that interpretations involving the *Technical Targets* dimension should be considered with caution, as manual validation showed lower agreement for this category compared with the other classification dimensions, primarily due to reduced recall.

• The keyword-level validation set is small (n = 30), which yields wide confidence intervals on per-dimension precision and recall. Reporting 95% confidence intervals (or noting the sample-size limitation) would be appropriate.

> In response to the second point, we added a sentence in the Methods section noting that the keyword-level validation was performed on a relatively small random subset (n = 30), and that category-specific performance estimates should therefore be interpreted as approximate indicators of overall agreement patterns across classification dimensions.

The new validation analysis is methodologically appropriate. Sampling is stratified, seeds are fixed, and metrics are standard (TP/FP/FN/TN, precision/recall/F1, with both micro- and macro-averaging). Validation code, sampling tables, and downstream supplementary tables are deposited in the manuscript GitHub repository. Reproducibility documentation has improved substantially from the previous version, including direct links to all supplementary files in the repository README.

> We thank the reviewer again for these constructive suggestions, which helped improve the clarity and interpretation of the validation analysis in the manuscript.
