# Supplementary material for: Microbiology Galaxy Lab: a community-driven gateway for reproducible and FAIR analysis of microbiological data
Source: Commun Biol. 2026 Oct 29;9:1425. doi: 10.1038/s42003-026-10750-7 (PMC13720100; doi:10.1038/s42003-026-10750-7)
Supplement: Supplementary file 3 [file 42003_2026_10750_MOESM2_ESM.pdf]

Supplementary information

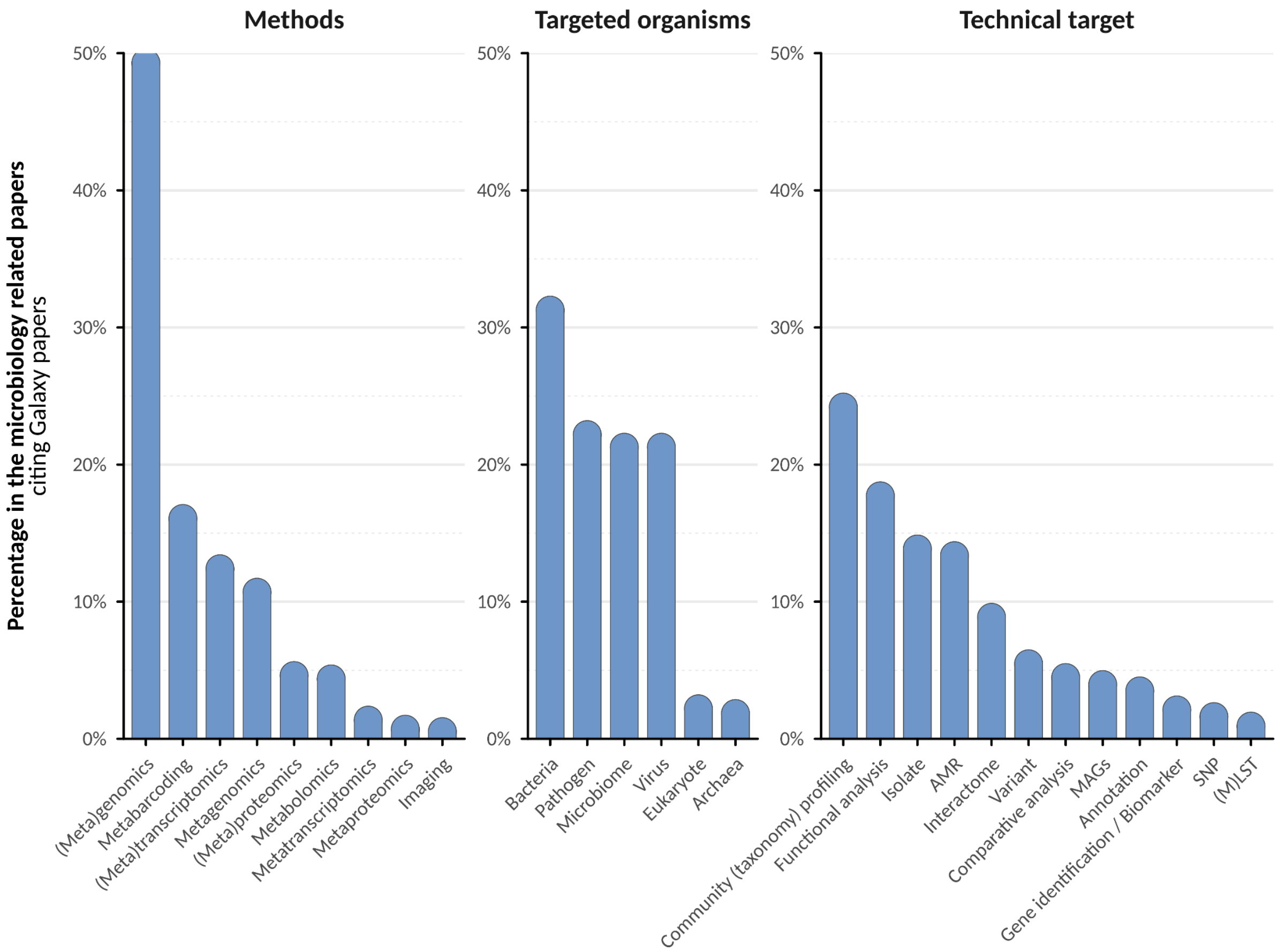

Supplementary Figure 1: Microbial Research Topics in Galaxy Publications.

Breakdown of microbiology-focused citations by Methods, Targeted Organisms, and Technical Targets. Methods described with "meta" in brackets, such as (meta)genomics, encompass both meta- and isolate-based analyses. Categories were annotated based on predefined keywords found in the title and abstract of each publication, detailed in **Supplementary Figure 5**.

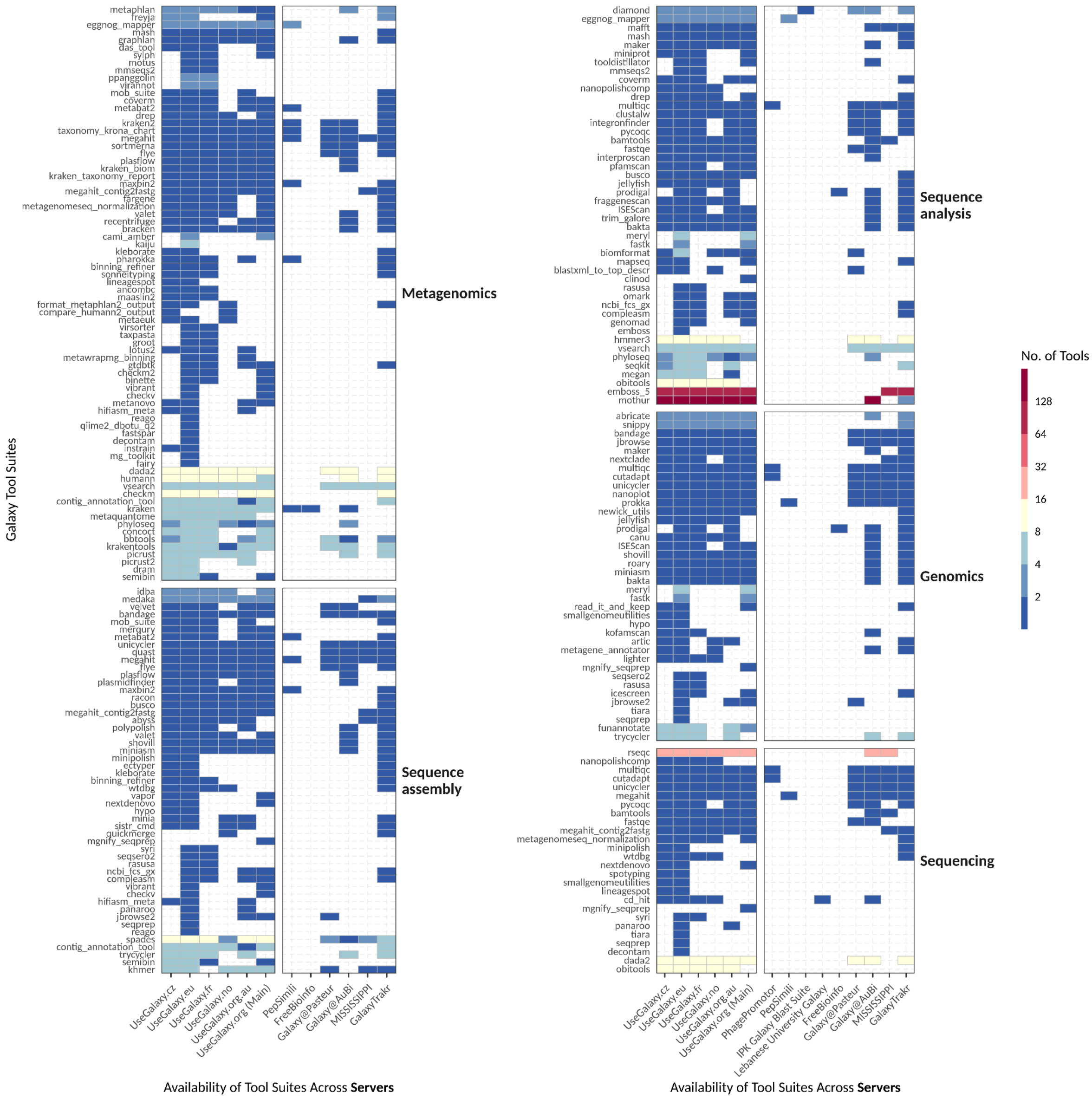

Supplementary Figure 2: Availability of microbiology-related tool suites within the Galaxy ecosystem. Heatmap illustrating the availability of microbiology-related tool suites (y-axis) across various Galaxy servers (x-axis), grouped by the most commonly annotated EDAM topics (top five EDAM topics). Tool suites may appear in multiple groups, as they can be annotated with several topics. A logarithmic scale is applied for better visualisation.

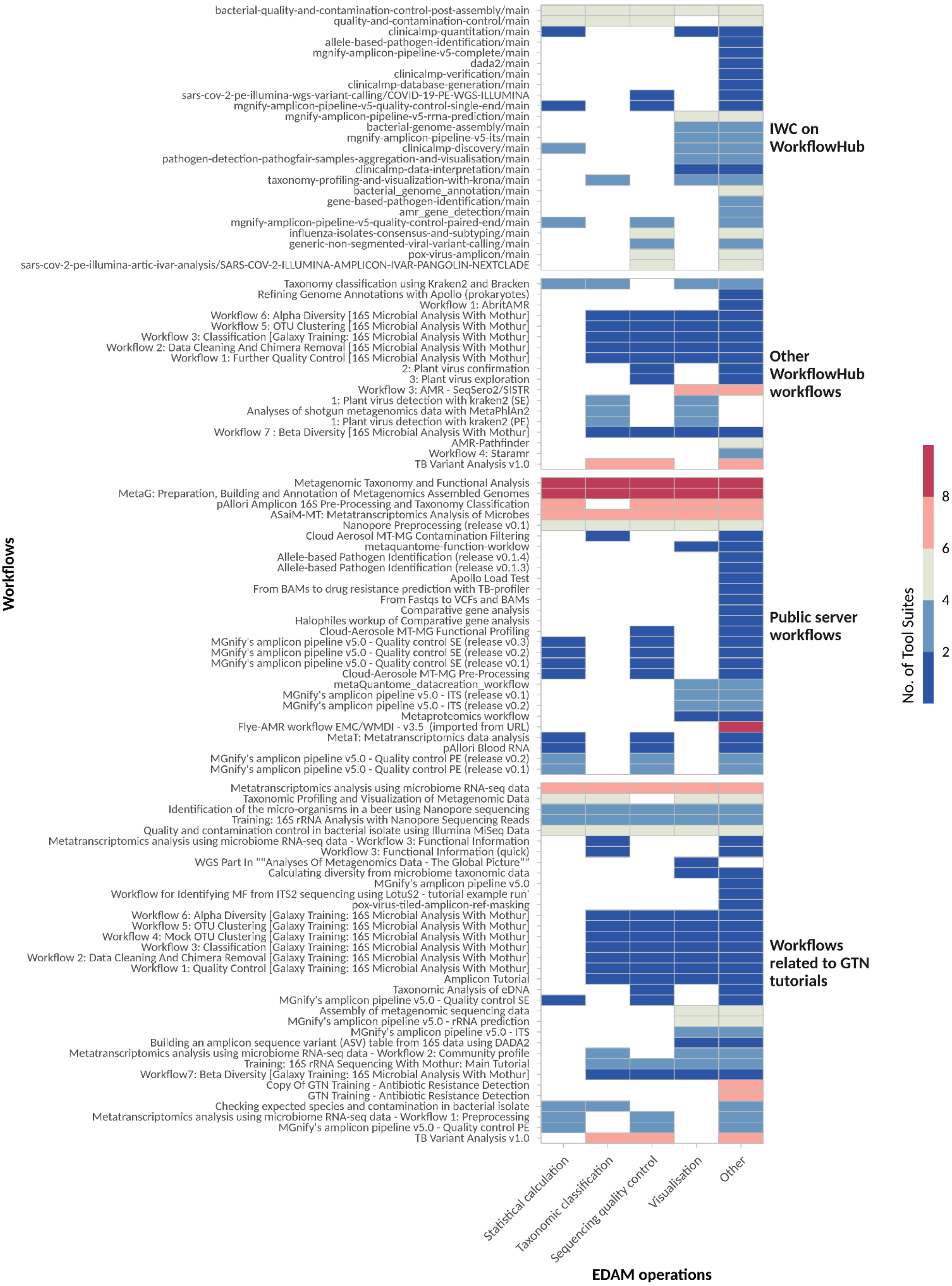

Supplementary Figure 3: Usage of microbiology-related tool suites across workflows.

Heatmap illustrating the number of microbiology-related tool suites across the available microbiology-related workflows, grouped by four levels of development. The tool suites are

organised based on their corresponding EDAM operations, highlighting the breadth of tools utilised for different workflows.

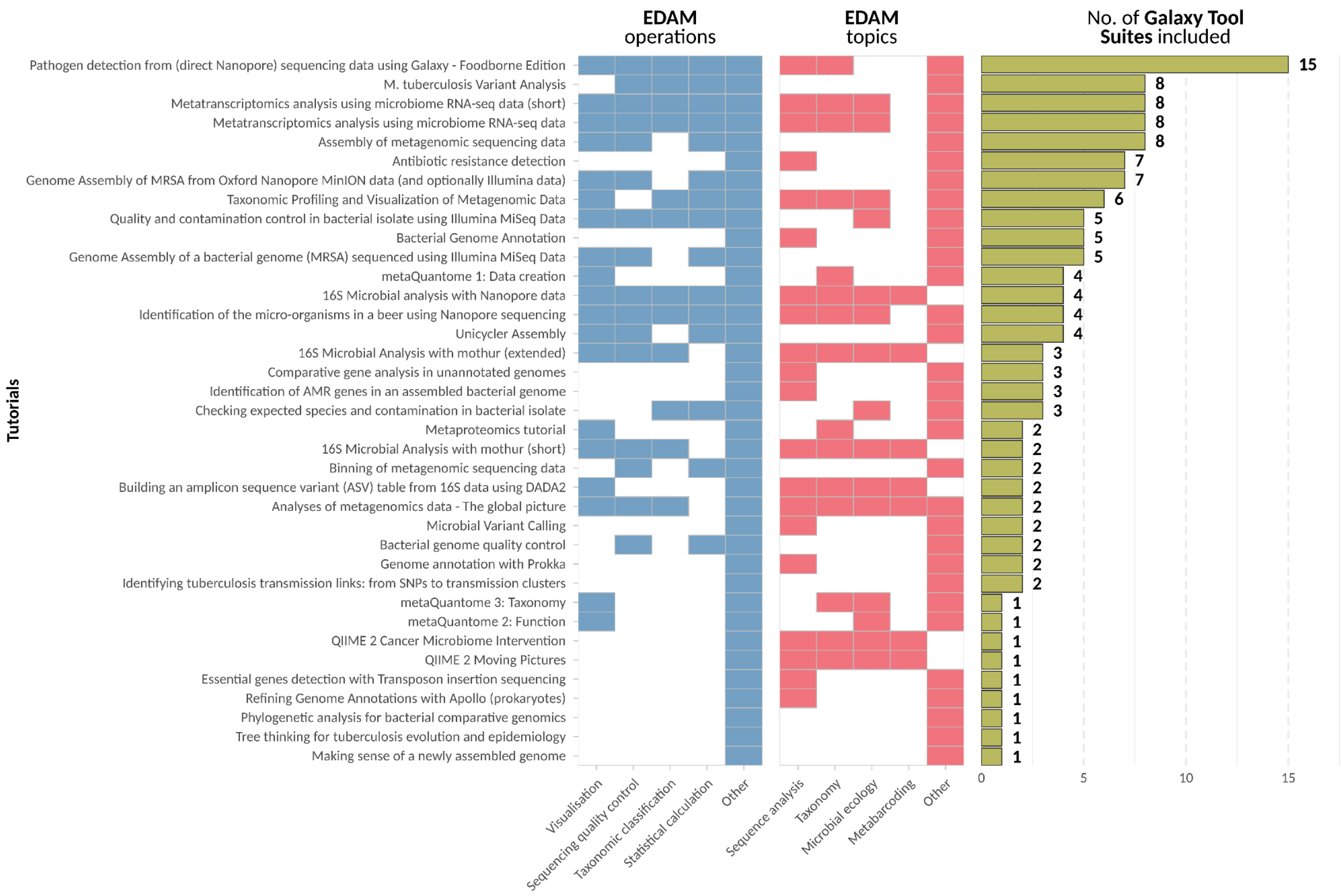

Supplementary Figure 4: Usage of microbiology-related tool suites across training materials. Heatmap illustrating the presence of microbiology-related tool suites within the available microbiology-related training materials, grouped by EDAM operations (blue), EDAM topics (red), and number of Galaxy tool suites used in the tutorials (green), highlighting the extent of tools utilised for different training contexts.

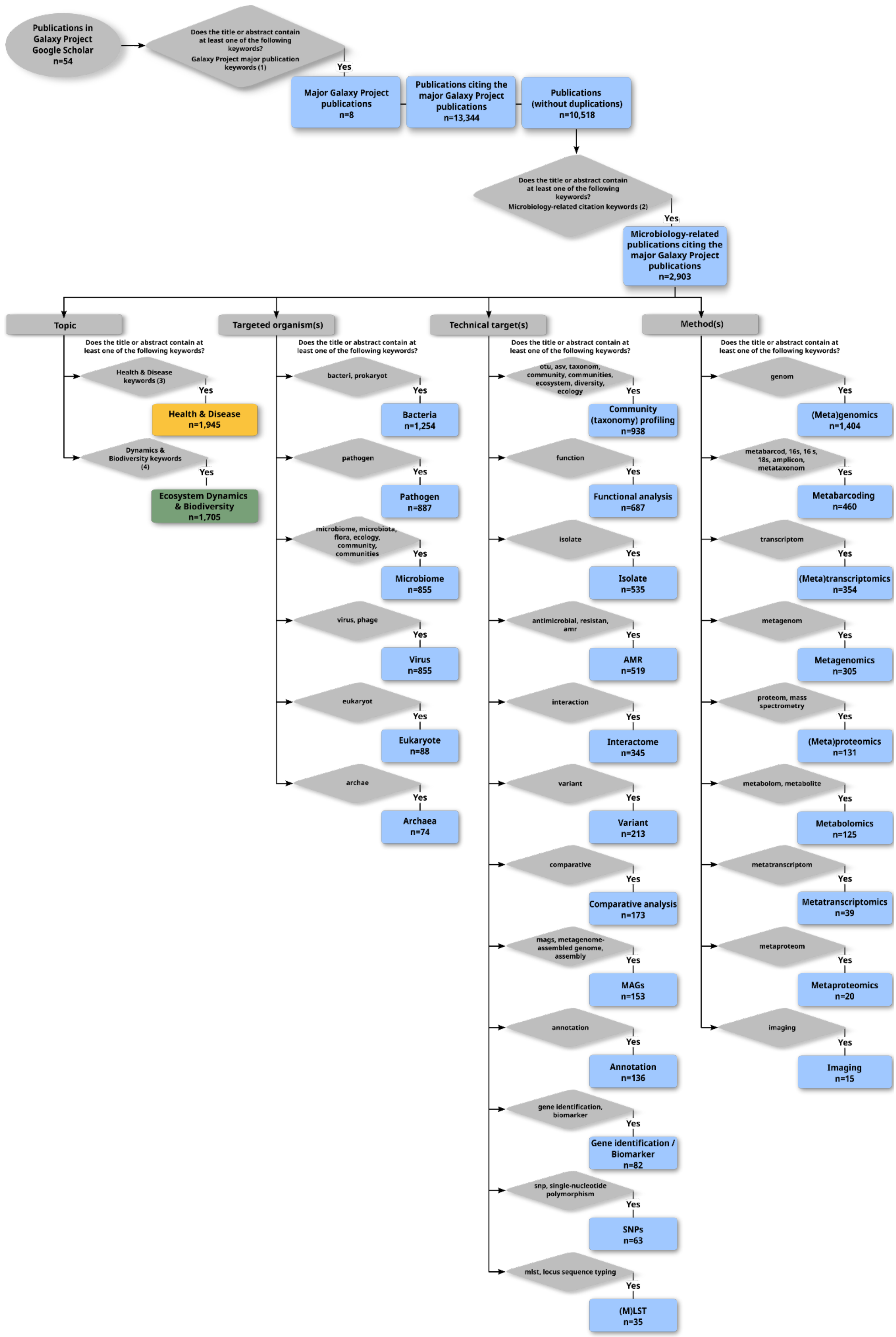

Publications in Galaxy Project Google Scholar n=54
Does the title or abstract contain at least one of the following keywords? Galaxy Project major publication keywords (1)
Yes
Major Galaxy Project publications n=8
Publications citing the major Galaxy Project publications n=13,344
Publications (without duplications) n=10,518
Does the title or abstract contain at least one of the following keywords? Microbiology-related citation keywords (2)
Yes
Microbiology-related publications citing the major Galaxy Project publications n=2,903
Topic
Targeted organism(s)
Technical target(s)
Method(s)
Does the title or abstract contain at least one of the following keywords?
Health & Disease keywords (3)
Yes
Health & Disease n=1,945
Dynamics & Biodiversity keywords (4)
Yes
Ecosystem Dynamics & Biodiversity n=1,705
Does the title or abstract contain at least one of the following keywords?
bacteri, prokaryot
Yes
Bacteria n=1,254
pathogen
Yes
Pathogen n=887
microbiome, microbiota, flora, ecology, community, communities
Yes
Microbiome n=855
virus, phage
Yes
Virus n=855
eukaryot
Yes
Eukaryote n=88
archae
Yes
Archaea n=74
Does the title or abstract contain at least one of the following keywords?
otu, asv, taxonom, community, communities, ecosystem, diversity, ecology
Yes
Community (taxonomy) profiling n=938
function
Yes
Functional analysis n=687
isolate
Yes
Isolate n=535
antimicrobial, resistan, amr
Yes
AMR n=519
interaction
Yes
Interactome n=345
variant
Yes
Variant n=213
comparative
Yes
Comparative analysis n=173
mags, metagenome-assembled genome, assembly
Yes
MAGs n=153
annotation
Yes
Annotation n=136
gene identification, biomarker
Yes
Gene identification / Biomarker n=82
snp, single-nucleotide polymorphism
Yes
SNPs n=63
mlst, locus sequence typing
Yes
(M)LST n=35
Does the title or abstract contain at least one of the following keywords?
genom
Yes
(Meta)genomics n=1,404
metabarcod, 16s, 16 s, 18s, amplicon, metataxonom
Yes
Metabarcoding n=460
transcriptom
Yes
(Meta)transcriptomics n=354
metagenom
Yes
Metagenomics n=305
proteom, mass spectrometry
Yes
(Meta)proteomics n=131
metabolom, metabolite
Yes
Metabolomics n=125
metatranscriptom
Yes
Metatranscriptomics n=39
metaproteom
Yes
Metaproteomics n=20
imaging
Yes
Imaging n=15

Supplementary Figure 5: Flowchart illustrating the process to select and annotate the microbiology-related publications citing the Galaxy Project's major publication. After choosing the major Galaxy Project publications by filtering publications present in the Galaxy Project Google Scholar based on predefined keywords (List 1 below) found in titles or abstracts, the publications citing them are extracted, and duplications are removed. The unique publications are first screened for microbial relevance based on 25 predefined keywords (List 2 below) found in titles or abstracts. Microbiology-related citations were classified into topic(s), targeted organism(s), technical target(s), and method(s) based on the presence or absence of specific thematic keywords, numbered or mentioned directly on the figure. Lists of keywords: (1) Galay Project major citations keywords: 'platform', 'a comprehensive approach', 'a web-based genome analysis tool for experimentalists'. (2) Microbiology-related citations keywords: 'bacteri', 'prokaryot', 'microb', 'pathogen', 'virus', 'phage', 'archae', 'flora', 'microecology', 'microorganism', 'micro-organism', 'microbiome', 'microbiota', 'metabarcod', '16s', '18s', 'amplicon', 'metataxonom', 'metagenom', 'metatranscriptom', 'metaproteom', 'multi-locus sequence typing', 'multilocus sequence typing', 'mlst', 'otu'. (3) Health & Disease keywords: 'health', 'disease', 'pathogen', 'antibiotic', 'epidemio', 'clinic', 'syndrom', 'antimicrob', 'patient', 'cancer', 'covid', 'hiv', 'diagnostic', 'food safety', 'toxic', 'infest', 'infect', 'prebiotic', 'probiotic', 'obesity', 'immun', 'food safety', 'gut microbiota', 'dysbiosis', 'host-microbe', 'immunomodulation', 'inflammation', 'gut-brain axis', 'metabolic syndrome', 'FMT', 'SCFA', 'healthcare', 'virulence', 'drug'. (4) Dynamics & Biodiversity keywords: 'environment', 'diversity', 'ecosystem', 'plant', 'corrosion', 'geograph', 'compos', 'fish', 'soil', 'biofilm', 'water', 'aquaculture', 'ecolog', 'ferment', 'ocean', 'marine', 'decomp', 'biofuel', 'permafrost', 'microbial mat', 'rhizosphere', 'grassland', 'biodegrada', 'biogeochem', 'succession', 'trophic', 'food web', 'community assembly', 'resilience', 'stability', 'niche', 'alpha diversity', 'beta diversity', 'symbiosis', 'competition', 'functional diversity', 'phylogen', 'river', 'crop', 'bird', 'cyanobacteri', 'auquatic'.

Supplementary Table 1: Features and capabilities of 45 existing user-friendly microbiological data analysis platforms (non-exhaustive list). The table includes columns detailing general characteristics (e.g., free usage, open source, workflow/pipeline manager), accessibility and availability (e.g., simple end-user modification, user-friendly interface, publicly available web server, automatable API, last update), user support and documentation (e.g., tutorials, documentation, user support), possible methods used to generate input data (e.g., isolate genomics, amplicon/metabarcoding, WGS/metagenomics, metatranscriptomics), and supported analyses (e.g., QC, taxonomy profiling, MAGs, comparative omics, multi-omics integration, interactive visualisations).

Supplementary Table 2: Anonymous results from the microbiology research community survey conducted between March and September 2023. The table includes aggregated and anonymised responses to survey questions, detailing researchers' demographics, tool usage, training needs, and suggestions for future improvements to the Galaxy platform.

Supplementary Table 3: Publications citing the Galaxy Project from Google Scholar. The table includes all publications citing at least one of 8 major Galaxy Project publications, with columns detailing the title and year of publication for each citing work.

Supplementary Table 4: Publications citing the Galaxy Project from Google Scholar classified as microbiology-related citations. The table includes publications from **Supplementary Table 3** that were classified as microbiology-related, following the criteria illustrated in **Supplementary Figure 5**. The table provides detailed classifications for each publication, including topic(s), targeted organism(s), technical target(s), and method(s).

Supplementary Table 5: Overview of the 319 microbiology-related tool suites available in the Microbiology Galaxy Lab (MGL). The table includes detailed metadata for each tool suite, such as suite ID, tool IDs, description, first commit date, homepage, version, conda package details, version status, and ToolShed categories. It also incorporates EDAM annotations (operations, reduced operations, topics, and reduced topics) and links to external resources like bio.tools (ID, name, and description). Usage statistics from Galaxy's

main servers over the last five years, including the number of users and tool runs, are also provided to highlight adoption and activity levels.

Supplementary Table 6: Tool suites corresponding to numbers on Figure 4. The table describes the tool suites that are presented in Figure 4 branches as numbers. The possible tool input and outputs, tools included in the suite, and corresponding RRID and biotools IDs are listed.

Supplementary Table 7: Comprehensive list of the 117 microbiology-related workflows available in the Microbiology Galaxy Lab (MGL). This table documents 117 workflows, detailing their names, sources, unique identifiers, and links to relevant repositories. Additional information includes the creators, associated tags, creation and update timestamps, latest and available versions, number of steps, and tools used. The workflows are categorised by EDAM operations and topics, and information on licensing, DOI assignments, and related projects is provided.

Supplementary Table 8: Summary of use case submissions collected from survey participants and other highlighted use cases. The table includes anonymised details of each use case, such as the research question, experimental methods, data analysis approaches, Galaxy tools and workflows used, and specific benefits of using Galaxy. It also highlights the status of the use cases (e.g., starting, ongoing, completed) and any associated publications. This summary serves as a foundation for the Results use cases subsection and the Discussion section of the manuscript.

Supplementary Table 9: Comprehensive list of tutorials from the Galaxy Training Network (GTN) dedicated to microbiological data analysis. This table documents 39 tutorials tagged with the term "microGalaxy" (https://training.galaxyproject.org/training-material/search2?query=microgalaxy), providing detailed metadata on each tutorial, including its topic, title, link, EDAM topic and operation annotations, creation and last modification dates, and version history. The table also includes information on available training materials such as slides, videos, workflows, and tool listings. Server support is detailed, distinguishing between precise tool versions and

different tool versions. User engagement metrics, including feedback count and mean rating, visitor numbers, page views, visit duration, and video views, highlight the usage and impact of these tutorials.

Supplementary Table 10: Overview of microbiology-related training events conducted between 2019 and 2025. The table provides details for 40 events, including their start and end dates, titles, covered topics, target audiences, and the specific contributions of the microGalaxy community when the event encompassed broader subjects beyond microbiological data analysis. Links to event pages and the resources utilised during the training sessions are also included.

Supplementary Table 11: Manual binary document-level validation of microbiology-related citation classification. The table contains the manually reviewed stratified validation dataset comprising 5% of citations automatically classified as microbiology-related (**Supplementary Table 4**) and 5% of citations not classified as microbiology-related (**Supplementary Table 3**, excluding overlapping titles). For each publication, the automated binary classification and the corresponding manual decision are reported, which form the basis for calculating TP, FP, FN, TN, precision, recall, F1-score, and accuracy.

Supplementary Table 12: Manual multi-label keyword-level validation of automated microbiology citation classification. The table contains the manually annotated 1% random subset of microbiology-related citations (**Supplementary Table 4**). For each publication, automated and manual assignments are reported across the four category dimensions (Targeted Organisms, Technical Targets, Methods, and Topics), enabling keyword-level comparison and computation of TP, FP, FN, TN, and category-level performance metrics.

Supplementary Method 1: List of survey questions shared with the microbiology research community via a Google Form between March and September 2023. The survey aimed to assess researchers' needs, challenges, and preferences in using microbiological data analysis tools and platforms.

Evaluation of the current usage and future needs of Galaxy for microbial data analysis

Galaxy is an **open-access, web-based platform for data-intensive computational research**. It is used in a variety of domains including **microbial data analysis**: for microbiome samples or bacterial isolates, long reads or short, shotgun or 16S, genomics, transcriptomics, or proteomics.

microGalaxy, a community of practice focused on microbial data analysis in Galaxy, would like to **learn** more about **use cases** where Galaxy was beneficial, and also the barriers **that prevent researchers from using Galaxy**. The aim is to collectively **find solutions** to improve user experience and reduce barriers**.** To this end, we are launching a **survey** to reach out to, and learn from, the microbial research community.

We encourage anyone to fill out this survey who:

- has used Galaxy for any kind of microbial focused research (e.g. metagenomics, metatranscriptomics, microbiome, bacterial isolates …), or
- conducts microbial research or is planning to do so, but has not yet used Galaxy, or is hindered by certain aspects of Galaxy from using it.

You can find more information about this survey and how we plan to use the information collected on the dedicated post here:
https://galaxyproject.org/news/2023-03-21-microbial-survey/

Thank you for your help supporting better microbial data analysis in Galaxy!

Can you give a short description of is your research area?

E.g.: I investigate the microorganisms in marine habitats to try and understand how they adapt to changing climatic conditions.)

<Free text>

What is your main target?

<One answer to select>

- Virus
- Bacteria
- Archaea
- Eukaryotes
- Mixed Communities

Which techniques do you use?

<Several answers can be selected>

- Single organism genomics
- Single organism transcriptomics
- Amplicon / metabarcoding
- Metagenomics
- Transcriptomics or metatranscriptomics
- Metabolomics
- Proteomics or metaproteomics
- Others

Which analysis do you use or would like to do?

<Several answers can be selected>

- Taxonomic profiling
- Pathogen tracking
- Assembly
- Antimicrobial resistance (AMR) detection
- Functional analysis
- Gene identification
- Single nucleotide polymorphism (SNP) identification
- Metagenome assembled genome (MAG) building
- Predictive metagenomics
- Comparative analyses
- Combination of multiomic approaches
- Phylogenetic tree construction
- Other

Which tools, platforms or databases are you using to conduct your research analyses

(e.g. Anvi'o, RStudio, nr, etc.)?

<Free text>

If the tools, platforms or databases from the previous question could be deployed in Galaxy would use prefer to use in there ?

<One answer to select>

- Yes
- No

How often do you use Galaxy?

<One answer to select>

- Never
- Less than once per year
- Several times per year
- Monthly
- Weekly
- Daily

If you never or rarely use Galaxy, what are the reasons?

<Free text>

Which Galaxy server(s) do you use?

<Several answers can be selected>

- Main, usegalaxy.org
- Galaxy Europe, usegalaxy.eu
- Galaxy Australia, usegalaxy.org.au
- Galaxy France, usegalaxy.fr
- Galaxy Norway, usegalaxy.no
- Galaxy Africa, africa.usegalaxy.eu
- Galaxy India, india.usegalaxy.eu
- Local Galaxy instance
- Other

Which tools and databases do you think are missing in Galaxy?

<Free text>

What features, tools, workflows etc. would you like to have in Galaxy that would support your research?

<Free text>

Which training resources are you using?

<Free text>

Which country are you based in?

<Free text>

What is your career stage?

<One answer to select>

- Undergraduate
- Graduate student

- PhD student
- Postdoctoral researcher
- Principal Investigator
- Staff scientist
- Other

Would you be interested in being contacted by the microGalaxy team and contributing an example use case to a manuscript?

<One answer to select>

- Yes
- No

If yes, please provide an email address

<Free text>

Do you have link(s) to publications or websites where we can read more about your research? If so, please add these below.

<Free text>

Supplementary Method 2: Template to collect detailed use cases from survey participants who expressed interest in contributing to the planned manuscript. The document includes sections for contact details, research questions, methods, tools, workflows, and results, along with prompts for providing a detailed description of the use cases. Preliminary survey results are also shared to provide context and highlight the motivation for the study.

Microbial data analysis in Galaxy

Case studies

*You filled out our form "Evaluation of uses and needs for microbial data analysis in Galaxy". Thanks to you and the community, we collected **more than 100 answers**, which has given us valuable information about the community.*

*In your survey answer, you showed interest in being contacted by the microGalaxy team and contributing an example use case to the planned manuscript. You already gave us some basic information in the form, but **we would appreciate a more detailed description of your work**. We therefore request that you fill out the fields below **before August 31st**, so that we have a good overview of your use case.*

*We plan to **review and group case studies by topics,** and then **highlight especially intriguing ones** (after discussion with the community and you) in the main text of the paper. **All submitted studies (including yours) will be included in the Supplementary material,** and we invite the authors of the selected studies to become **coauthors of the manuscript.***

Thank you for your help!

Contact

First name	Last name	Email	Affiliation	Country	ORCID

Case study

Title

Status

Possible answers: Starting / On-going / Completed / Other

Publications

Funding

Keyword
Give 3-8 keywords describing your use case

Research question

Main target
Keywords. E.g. AMR, Pathogen, Microbiome community, MAGs building, Functional analysis, Gene identification, Comparative analysis, SNP analysis, or anything else

Short description of the research questions of your case study
Images can also be included

Methods

Experimental or Measurement technique used
E.g. metagenomics, metabarcoding, amplicon, proteomics, metabolomics, transcriptomics, single organism genomics

Data generation approach
E.g. Illumina, Nanopore, LS/MS

Data analysis approach

E.g. quality control, taxonomy profiling, assembly, differential gene expression, statistical analysis

Tools, workflows or platforms outside Galaxy

Galaxy tools used

Workflow(s) used

Specific benefits of using Galaxy

E.g. access to computational power, access to databases, tool availability, easy-to-use workflows, reproducibility, bulk processing using collections, or anything else

Short description of the methods you used

Images can also be included

Interesting results

Short description of the results you got
Images can also be included

Other

Anything else you would like to mention about your use case?

References

Teaser of survey’s results

If you are interested to have a first peek at the results of the survey, here are some figures based on the first 90 answers.

The figure below shows the main techniques used by the participants.

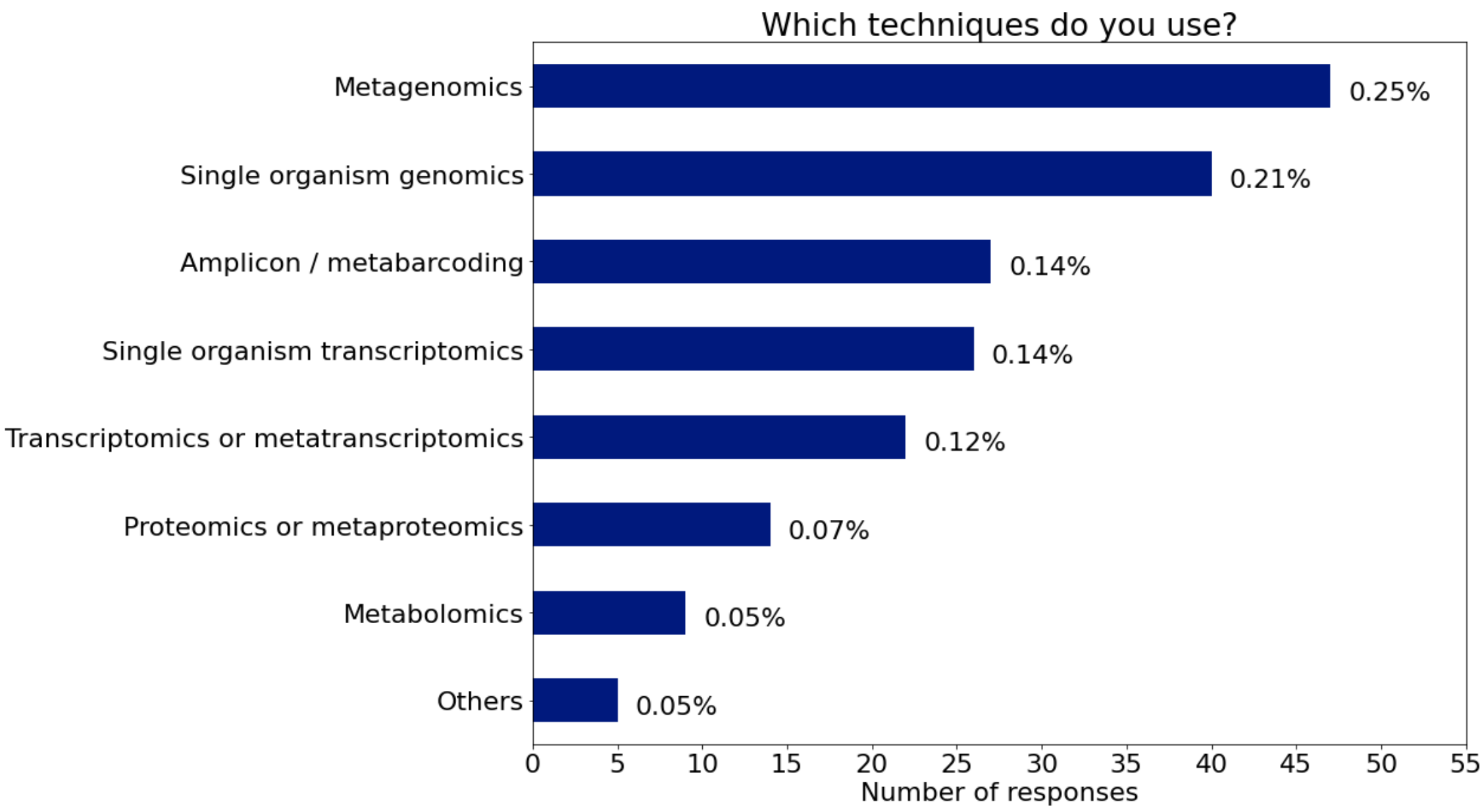

We also discovered that a large majority of the respondents would use Galaxy for their analyses if the features they require were available on the platform. This motivates us to write this paper and take the next steps towards improving Galaxy for the microbial analysis community.

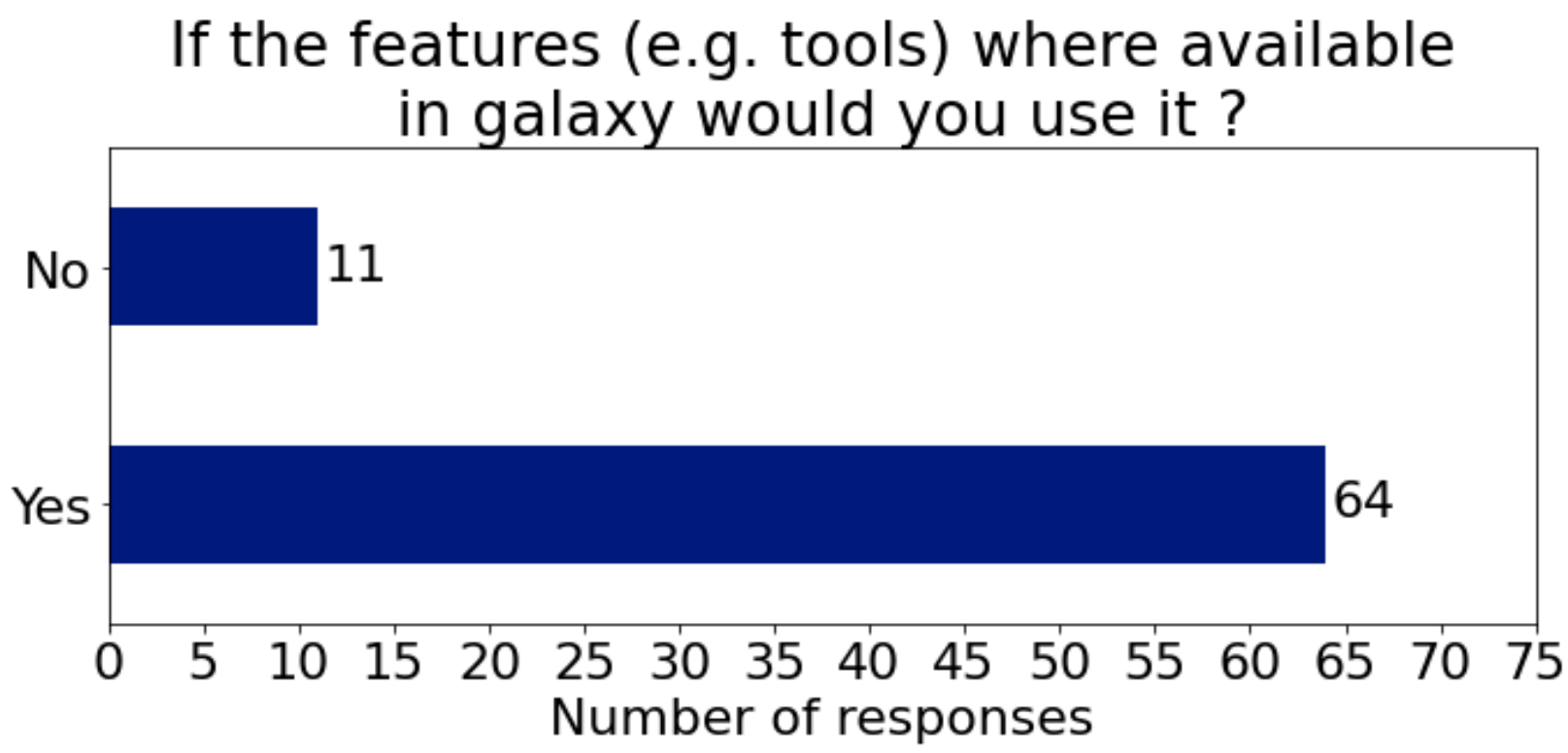

Supplementary Note 1: Manual validation framework for automated citation classification. The document describes the design, implementation, and results of the manual validation procedures conducted to assess the performance of the automated citation extraction and classification pipeline. It outlines the reproducible sampling strategy, manual annotation criteria, and statistical evaluation methods used for both keyword-level (multi-label) and document-level (binary) validation, and reports the full performance metrics underlying the results presented in the manuscript.

Validation of the Automated Citation Classification Framework

Overview

To evaluate the robustness and reliability of the automated citation-mining framework, two independent validation procedures were conducted:

1. Binary document-level validation, assessing whether publications were correctly classified as microbiology-related or non-microbiology-related.
2. Multi-label keyword-level validation, assessing the agreement between automated and manual assignments across four predefined classification dimensions.

All validation procedures were implemented in a Jupyter Notebook (**explore_publications_manual_validation.ipynb**) using fixed random seeds to ensure deterministic sampling and fully reproducible results.

Binary Document-Level Validation

To evaluate document-level classification (microbiology-related vs. non-microbiology-related)

Sampling Strategy

A validation dataset was constructed as follows:

- 5% of entries from **Supplementary Table 4** (microbiology-related citations)
- 5% of entries from **Supplementary Table 3** (all citations)

Title normalisation was used to ensure exact matching between tables. This resulted in a combined validation set of 549 publications.

Sampling and dataset construction were fully reproducible via fixed random seeds.

Evaluation Procedure

To evaluate whether the automated system correctly distinguishes microbiology-related from non-microbiology-related citations, binary classification metrics were computed.

For each manually reviewed publication:

- Positive class: microbiology-related
- Negative class: non-microbiology-related

The following were computed:

- True Positive (TP): correctly identified microbiology-related publications
- False Positive (FP): non-microbial publications incorrectly labeled as microbial
- False Negative (FN): microbial publications missed by the classifier
- True Negative (TN): correctly identified non-microbial publications

Precision, recall, F1-score, and overall accuracy were calculated.

Results

From 549 manually evaluated publications, documented in **Supplementary Table 11**:

Metric	Value
TP	134
FP	19
FN	38
TN	358
Precision	87.6%
Recall	77.9%
F1-score	82.5%
Accuracy	89.6%

The high precision indicates that when the classifier assigns microbiology-related status, it is correct in the vast majority of cases. The lower but substantial recall indicates a conservative tendency, with some microbiology-relevant publications not being detected.

False positives were limited, suggesting minimal contamination of the microbiology-related corpus, while false negatives indicate that borderline or implicitly microbiology-relevant studies may occasionally be excluded.

Multi-label Keyword-Level Validation

To evaluate keyword-level classification across four predefined classification dimensions

Sampling Strategy

A random subset of 1% of **Supplementary Table 4** (microbiology-related citations until the end of 2025) was selected using a fixed seed (seed = 42). This resulted in 30 publications for manual review, which are listed in **Supplementary Table 12**.

Classification dimensions

Each publication was classified across four dimensions:

1. Targeted Organisms
2. Technical Targets
3. Methods
4. Topics

Each dimension consists of a predefined keyword universe.

Because publications may contain multiple keywords within a dimension, evaluation was conducted at the keyword level, not at the binary category level.

Evaluation Procedure

For each publication and each dimension:

- Let A be the set of automated keywords.
- Let M be the set of manually assigned keywords.
- Let U be the predefined keyword universe for that dimension. These keywords are predefined for every dimension/category, shown in details in **Supplementary Figure 5**.

The following were computed:

- True Positive (TP) = $|A \cap M|$, keywords present in both automated and manual annotations
- False Positive (FP) = $|A - M|$, keywords assigned automatically but not confirmed manually
- False Negative (FN) = $|M - A|$, keywords present in manual annotation but missed by the automated classifier
- True Negative (TN) = $|U - (A \cup M)|$, keywords assigned by neither approach

This set-based approach allows partial agreement. For example, if the automated classifier assigns multiple keywords and only some are manually confirmed, both TP and FP are recorded.

Category-level performance was obtained by summing TP, FP, FN, and TN across all publications.

Precision, recall, and F1-scores were computed per category.

Micro- and Macro-Averaging

Two averaging strategies were applied:

- Macro-average: arithmetic mean of per-category metrics (equal weight per dimension).
- Micro-average: metrics computed by pooling TP, FP, and FN across all dimensions (weighted by the total number of keyword decisions).

Macro-averaging reflects balance across classification dimensions, whereas micro-averaging reflects overall system behavior.

Results

The automated classifier achieved:

- Micro-averaged F1-score: 63.4%
- Macro-averaged F1-score: 68.9%

Category-specific performance:

Category	Precision	Recall	F1-score
Topics	87.5%	89.7%	88.6%
Methods	93.1%	77.1%	84.4%
Targeted organisms	75.6%	63.0%	68.7%

Technical target	61.1%	23.4%	33.8%

Performance was strongest for Topics and Methods, moderate for Targeted organisms, and lowest for Technical targets, primarily due to reduced recall in that category.

Reproducibility

All validation procedures were implemented in a Jupyter Notebook available on GitHub.

This notebook:

- Performs reproducible random sampling using fixed seeds,
- Constructs validation subsets,
- Normalises keyword labels,
- Computes TP, FP, FN, TN at the keyword and document level,
- Generates summary statistics and exportable result tables.

Manually validated sampling tables are saved as TSV files to ensure transparency and auditability (https://github.com/usegalaxy-eu/microbiology_galaxy_lab_paper_2026/tree/main/docs/supplementary).

- Binary document-level manual validation of microbiology-related citation classification: **Supplementary Table 11**
- Multi-label keyword-level manual validation of automated microbiology citation classification: **Supplementary Table 12**

Conclusion

The combined validation results demonstrate that:

1. The automated framework reliably distinguishes microbiology-related literature from unrelated citations with high precision and strong overall F1 performance.
2. High-level thematic and methodological classification (Topics and Methods) is robust and consistent.
3. Fine-grained technical target assignments remain more challenging, primarily due to reduced recall.

These results support the use of the automated framework for large-scale trend analysis and ecosystem mapping, while identifying specific areas where further refinement may improve label sensitivity.

Supplementary Note 2: microGalaxy Community Roadmap (2026–2030), outlining the strategic priorities, goals, and planned developments for the Microbiology Galaxy Lab (MGL). The roadmap includes six core axes (consolidation of state-of-the-art resources, expansion of microbial data analysis capabilities, reference data management, FAIR data and reproducibility, scalability and computational efficiency, and training and community engagement) along with specific actions.

microGalaxy Community Roadmap

2026–2030

Core Principles:

- **Consolidate and curate** state-of-the-art tools, workflows, and tutorials.
- **Adapt quickly** to scientific and technological advancements.
- **Prioritize reference data management** as a foundational pillar.
- **Foster community-driven innovation** and open collaboration.

1. Consolidate and Curate State-of-the-Art Resources

Goal: Ensure MGL remains a **leading platform** for microbial bioinformatics by continuously updating, deprecating, and curating tools, workflows, and tutorials.

Actions:

- Regularly review and update tools and workflows to reflect the latest scientific advancements.
- Decommission outdated tools while preserving them for reproducibility.
- Expand and refine tutorials to cover emerging topics (e.g., AI/ML in microbiome analysis, metaproteomics, holo-omics).
- Curate learning pathways to guide users from beginner to advanced analyses.

2. Expand and Diversify Microbial Data Analysis Capabilities

Goal: Broaden MGL's scope to include **viruses, archaea, eukaryotes**, and **multi-omics integration**, while ensuring workflows meet the needs of specialized platforms and emerging research areas and integrating **One Health principles** to support cross-domain analyses (human/animal/environmental microbiomes).

Actions:

- Develop flagship workflows that showcase state-of-the-art capabilities in key domains, such as MAG generation, AMR detection and tracking pathogen spillover, advancing scientific capacity in these areas and supporting cross-domain analyses
- Develop workflows for emerging research areas (e.g., host-microbe interactions, environmental microbiome monitoring).
- Support multi-omics integration (e.g., metagenomics + metaproteomics + metabolomics).
- Develop tailored workflows to support Galaxy-based platforms like BRC-analytics and ABRomics
- Integrate pipelines from MGnify, KBase, and BV-BRC to expand capabilities for viral, fungal, and eukaryotic analysis.

3. Reference Data Management

Goal: Establish **shared, standardized, and accessible reference data** to support all MGL activities.

Actions:

- Collaborate with the Galaxy community to automate the addition of reference data to Galaxy CernVM-FS via Galaxy IDC (https://github.com/galaxyproject/idc).
- Develop Ansible roles for managing reference data installation via Git repositories.
- Integrate reference datasets from AllTheBacteria, Logan, MGnify, and other MAGs databases.
- Work with pipelines from MGnify, KBase, BV-BRC, and others to ensure their reference data is accessible and up-to-date in MGL.
- Curate "reference data packs" for common use cases (e.g., AMR genes, 16S rRNA, viral genomes).

4. Strengthen FAIR Data and Reproducibility

Goal: Maintain **FAIR compliance** and **reproducibility** as the field evolves.

Actions:

- Automate metadata annotation for new tools and workflows using EDAM ontology and bio.tools.
- Expand DOI assignment for workflows and reference datasets via WorkflowHub and Zenodo.
- Promote RO-Crate exports to ensure complete, reproducible analysis environments.
- Integrate with global repositories (e.g., EMBL-EBI, MGnify) for seamless data sharing.

5. Improve Scalability and Computational Efficiency

Goal: Optimize MGL for **large-scale analyses** and **distributed computing**.

Actions:

- Develop and refine AI-driven resource allocation to predict computational needs.
- Facilitate federated analysis across Galaxy instances through standardized availability of tools and reference data on public, private, and local Galaxy deployments.

6. Enhance Training and Community Engagement

Goal: Empower users through **comprehensive training** and **global collaboration**.

Actions:

- Continue to offer tracks at the annual Galaxy Training Academy
- Host annual hackathons to co-create tools, workflows, and reference data standards.
- Partner with organizations like ELIXIR and Australian BioCommons for localized training.
- Encourage community contributions to training materials and workflows.
