# Supplementary material for: Microbiology Galaxy Lab: a community-driven gateway for reproducible and FAIR analysis of microbiological data
Source: Commun Biol. 2026 Oct 29;9:1425. doi: 10.1038/s42003-026-10750-7 (PMC13720100; doi:10.1038/s42003-026-10750-7)
Supplement: Supplementary file 4 [file 42003_2026_10750_MOESM3_ESM.pdf]

Descriptions of Additional Supplementary Files

Supplementary Data 1: Features and capabilities of 45 existing user-friendly microbiological data analysis platforms (non-exhaustive list). The table includes columns detailing general characteristics (e.g., free usage, open source, workflow/pipeline manager), accessibility and availability (e.g., simple end-user modification, user-friendly interface, publicly available web server, automatable API, last update), user support and documentation (e.g., tutorials, documentation, user support), possible methods used to generate input data (e.g., isolate genomics, amplicon/metabarcoding, WGS/metagenomics, metatranscriptomics), and supported analyses (e.g., QC, taxonomy profiling, MAGs, comparative omics, multi-omics integration, interactive visualisations).

Supplementary Data 2: Anonymous results from the microbiology research community survey conducted between March and September 2023. The table includes aggregated and anonymised responses to survey questions, detailing researchers' demographics, tool usage, training needs, and suggestions for future improvements to the Galaxy platform.

Supplementary Data 3: Publications citing the Galaxy Project from Google Scholar. The table includes all publications citing at least one of 8 major Galaxy Project publications, with columns detailing the title and year of publication for each citing work.

Supplementary Data 4: Publications citing the Galaxy Project from Google Scholar classified as microbiology-related citations. The table includes publications from **Supplementary Data 3** that were classified as microbiology-related, following the criteria illustrated in **Supplementary Figure 5**. The table provides detailed classifications for each publication, including topic(s), targeted organism(s), technical target(s), and method(s).

Supplementary Data 5: Overview of the 319 microbiology-related tool suites available in the Microbiology Galaxy Lab (MGL). The table includes detailed metadata for each tool suite, such as suite ID, tool IDs, description, first commit date, homepage, version, conda

package details, version status, and ToolShed categories. It also incorporates EDAM annotations (operations, reduced operations, topics, and reduced topics) and links to external resources like bio.tools (ID, name, and description). Usage statistics from Galaxy's main servers over the last five years, including the number of users and tool runs, are also provided to highlight adoption and activity levels.

Supplementary Data 6: Tool suites corresponding to numbers on Figure 4. The table describes the tool suites that are presented in Figure 4 branches as numbers. The possible tool input and outputs, tools included in the suite, and corresponding RRID and biotools IDs are listed.

Supplementary Data 7: Comprehensive list of the 117 microbiology-related workflows available in the Microbiology Galaxy Lab (MGL). This table documents 117 workflows, detailing their names, sources, unique identifiers, and links to relevant repositories. Additional information includes the creators, associated tags, creation and update timestamps, latest and available versions, number of steps, and tools used. The workflows are categorised by EDAM operations and topics, and information on licensing, DOI assignments, and related projects is provided.

Supplementary Data 8: Summary of use case submissions collected from survey participants and other highlighted use cases. The table includes anonymised details of each use case, such as the research question, experimental methods, data analysis approaches, Galaxy tools and workflows used, and specific benefits of using Galaxy. It also highlights the status of the use cases (e.g., starting, ongoing, completed) and any associated publications. This summary serves as a foundation for the Results use cases subsection and the Discussion section of the manuscript.

Supplementary Data 9: Comprehensive list of tutorials from the Galaxy Training Network (GTN) dedicated to microbiological data analysis. This table documents 39 tutorials tagged with the term "microGalaxy" (https://training.galaxyproject.org/training-material/search2?query=microgalaxy), providing detailed metadata on each tutorial, including its topic, title, link, EDAM topic and operation annotations, creation and last

modification dates, and version history. The table also includes information on available training materials such as slides, videos, workflows, and tool listings. Server support is detailed, distinguishing between precise tool versions and different tool versions. User engagement metrics, including feedback count and mean rating, visitor numbers, page views, visit duration, and video views, highlight the usage and impact of these tutorials.

Supplementary Data 10: Overview of microbiology-related training events conducted between 2019 and 2025. The table provides details for 40 events, including their start and end dates, titles, covered topics, target audiences, and the specific contributions of the microGalaxy community when the event encompassed broader subjects beyond microbiological data analysis. Links to event pages and the resources utilised during the training sessions are also included.

Supplementary Data 11: Manual binary document-level validation of microbiology-related citation classification. The table contains the manually reviewed stratified validation dataset comprising 5% of citations automatically classified as microbiology-related (**Supplementary Data 4**) and 5% of citations not classified as microbiology-related (**Supplementary Data 3**, excluding overlapping titles). For each publication, the automated binary classification and the corresponding manual decision are reported, which form the basis for calculating TP, FP, FN, TN, precision, recall, F1-score, and accuracy.

Supplementary Data 12: Manual multi-label keyword-level validation of automated microbiology citation classification. The table contains the manually annotated 1% random subset of microbiology-related citations (**Supplementary Data 4**). For each publication, automated and manual assignments are reported across the four category dimensions (Targeted Organisms, Technical Targets, Methods, and Topics), enabling keyword-level comparison and computation of TP, FP, FN, TN, and category-level performance metrics.
